# An integrated NMR field probe using rapid non-adiabatic field switch-off for (ultra) low field MRI

**DOI:** 10.1038/s44172-026-00815-0

**Published:** 2026-10-28

**Authors:** Pavel Povolni, Dominique Goerner, Praveen Iyyappan Valsala, Lukas Gebert, Georgiy Solomakha, Nicolas Kempf, Felix Glang, Judith Samlow, Ruben Schnitzler, Ingmar Kallfass, Kai Buckenmaier, Klaus Scheffler

**Affiliations:** 1 High‑Field Magnetic Resonance Center, Max Planck Institute for Biological Cybernetics, Tübingen, Germany; 2 Institute of Robust Power Semiconductor Systems ILH, University of Stuttgart, Stuttgart, Germany; 3 Department for Biomedical Magnetic Resonance, University of Tübingen, Tübingen, Germany; 4 Institute of Biomedical Imaging, Graz University of Technology, Graz, Austria; 5 Hospital Diospi Suyana, Curahuasi, Apurímac Peru

**Keywords:** Magnetic resonance imaging, Electrical and electronic engineering

## Abstract

Access to magnetic resonance imaging (MRI) remains severely limited in low- and middle-income countries, especially in *sub-Saharan Africa*, despite rising rates of non-communicable diseases. Low-field MRI presents an affordable, locally developable diagnostic solution, but its performance is constrained by magnetic field instability. We present a novel NMR field probe designed to overcome these challenges using a rapid non-adiabatic switch-off of a pre-polarization field resulting in precessing spin magnetization. Achieved by first use of high-voltage silicon carbide transistors operating in controlled avalanche breakdown, it measures the Larmor frequency without prior field knowledge, unlike conventional probes. This capability is crucial during magnet development with often unknown fields, allowing early detection of magnet issues, and offering an urgently needed tool for magnet design and image-quality improvement. Validated from 1 mT to 45 mT, up to 1,000 times stronger than similar systems, its low-cost, modular design supports replication, upgrades, and enhanced field control, helping expand global MRI access.

## Abstract (web summary)

Pavel Povolni and colleagues report an integrated NMR field probe for low-field MRI based on rapid non-adiabatic switch-off of a pre-polarization field using controlled avalanche breakdown in SiC transistors. The probe measures Larmor frequencies without prior field knowledge from 1 to 45 mT, covering field strengths relevant to ultra-low- and low-field MRI.

## Introduction

Almost a decade ago, the United Nations (UN) introduced the 17 Sustainable Development Goals (SDGs) to promote sustainable global development in underserved regions including global health and education^1,2^. Monitoring of the SDGs has repeatedly shown that the current progress is off track^3,4^ and highlighted persistent disparities in access to healthcare infrastructure worldwide, directly affecting progress toward SDG 3 (Good Health and Well-being). In particular, limited access to advanced medical imaging technologies remains a major challenge in many low- and middle-income countries (LMICs)^5^. Magnetic resonance imaging (MRI), a key diagnostic modality for diseases such as cancer, cardiovascular disorders, and neurological conditions, is strongly affected by this imbalance.

The disparity is particularly evident in sub-Saharan Africa. For example, Ogbole et al. report that West Africa, with a population exceeding 370 million, has access to only 84 MRI scanners (high- and low-field combined), corresponding to approximately 0.22 scanners per million inhabitants—more than 230 times fewer than Japan, the leader in this field^6^ (consistent with various other studies^7–11^). At the same time, the prevalence of non-communicable diseases is increasing as life expectancy rises in the region^12–15^, further emphasizing the need for accessible diagnostic imaging. One major reason for the limited availability of MRI is the high cost and infrastructure requirements of conventional high-field MRI (HFMRI) systems with main magnetic field strengths ${B}_{0}\ge 1.5{\rm{T}}$. These systems rely on superconducting magnets and cryogenic operation, which lead to high installation, maintenance, and operational costs. Consequently, deploying such systems in resource-limited healthcare environments remains challenging^6–11^.

Low-field MRI (LFMRI) systems provide a promising alternative. These systems typically operate at significantly lower magnetic field strengths and can be realized using permanent magnets or simple electromagnets^16–20^. Compared with HFMRI, LFMRI scanners are smaller, significantly less expensive, and require minimal infrastructure. Recent developments have demonstrated the feasibility of in-vivo imaging with permanent-magnet-based MRI systems operating at fields around ${B}_{0}\approx 50\mathrm{mT}$^17,21–23^. Due to their reduced hardware complexity, such systems can also benefit from open hardware approaches and have been successfully assembled and tested in local workshops, for example, in Uganda^17^. LFMRI systems can be realized using either permanent magnets or resistive electromagnets^16,24^. Permanent magnets enable higher magnetic field strengths and therefore increased Signal-to-Noise Ratio (SNR), but typically suffer from increased field inhomogeneity and temporal drift^19^. Electromagnets provide highly homogeneous and stable fields, but require additional power and cooling infrastructure. Emerging hyperpolarization techniques may further expand the potential of electromagnet-based systems in the future^25–28^.

A key enabling technology for LFMRI is accurate magnetic field measurement, since high-field homogeneity is required during image acquisition to ensure diagnostic quality. Permanent-magnet systems, therefore, require extensive shimming after assembly, whereas electromagnet-based systems depend on accurate coil alignment and continuous monitoring of temporal field drift. Therefore, the widespread deployment of low-field MRI also requires affordable tools for magnetic field characterization.

Several technologies are available for magnetic field measurement, including the well-known Hall effect^21,29,30^, integrated magnetoresistive sensors^31^, SQUIDs^32,33^, optically pumped magnetometers^34^, and NMR-based sensors^35^, to mention a few. Hall and magnetoresistive sensors are widely used because of their low cost, compact size, and robust operation. However, their magnetic field accuracy and long-term stability are generally insufficient for the high-precision field characterization required in LFMRI. At the opposite end of the performance spectrum, SQUIDs and optically pumped magnetometers provide exceptional magnetic field sensitivity. Their practical use in accessible LFMRI systems, however, is limited by the complexity of the required instrumentation and operating conditions. NMR-based sensors uniquely combine high accuracy, long-term stability, and a large dynamic range over the magnetic field range relevant for LFMRI^35^. Commercial NMR teslameters provide highly precise magnetic field measurements^36^, while self-built NMR probes^37,38^ and commercial field cameras^39,40^ have demonstrated dynamic field monitoring capabilities. However, transferring these approaches to LFMRI is fundamentally challenging because the NMR signal U decreases quadratically with the field strength ($U \sim {{B}_{0}}^{2}$). Consequently, compared with 3T-HFMRI, only 280 ppm of the signal remains at 50 mT^21^ and only 5 ppm at 6.5 mT^24^. Additionally, a large dynamic range is required for field monitoring of the LFMRI setup, especially when the exact field strength is not known in advance. As a result, high-precision NMR measurements are currently restricted to completed LFMRI systems^41^, whereas Hall sensors remain the standard tool for magnet construction and initial characterization^17,21^.

Here, we present an integrated NMR-based magnetic field sensor specifically designed for LFMRI based on an excitation method called non-adiabatic switch-off^42^. An integrated strong pre-polarization field perpendicular to ${B}_{0}$ increases the spin polarization significantly above the polarization at ${B}_{0}$. Switching it off abruptly prevents the adiabatic following of the magnetization as the field decays, resulting in a measurable transverse magnetization. The biggest advantage of this technique is that it operates without detailed prior knowledge of the magnetic field, unlike conventional excitation schemes, which may require the exact Larmor frequency to achieve an on-resonant condition for excitation of the spins. Instead, simply switching off the magnetic field quickly enough is sufficient to excite the spins. The sensor employs a non-adiabatic switch-off excitation generated by controlled avalanche operation of high-voltage n-type metal oxide field effect transistors made of silicon carbide (SiC-nFET), enabling compact generation of voltage pulses exceeding 2000 V and strong magnetic field ramps of $\Delta B=-70{\rm{T}}/\mathrm{ms}$. The usability of a non-adiabatic switch-off of a pre-polarization field for NMR excitation is a known concept that follows directly from the solutions of the Bloch equations when the transient magnetization dynamics are properly considered^43^. The approach has previously been employed primarily in very low magnetic fields, for example, in geophysical applications operating near the Earth’s magnetic field^44,45^. Compared with these earlier implementations, our concept achieves ramp-down times that are approximately three orders of magnitude faster, enabling measurements at magnetic fields up to three orders of magnitude higher. Furthermore, to the best of the authors’ knowledge, this work presents the first fully integrated, stand-alone sensor implementing this excitation scheme.

Furthermore, the hardware can also be used with a conventional RF excitation that includes standard adiabatic pre-polarization and has therefore been named *hybrid*.

The sensor is experimentally demonstrated in two representative LFMRI platforms spanning the major application space of modern LFMRI: an electromagnet-based ultra-low-field MRI (ULFMRI, ${B}_{0}=1-5\mathrm{mT}$) and a Halbach permanent-magnet head LFMRI system (${B}_{0}=45\mathrm{mT}$). This work should be regarded as a proof-of-concept demonstrating the feasibility of an integrated NMR field sensor used in LFMRI based on controlled avalanche-assisted non-adiabatic excitation. As a first implementation operating in these previously unexplored operating conditions, the primary objective is to establish the sensing concept and its practical realization while identifying the key engineering challenges for future optimization.

## Results

### Non-adiabatic switching off the pre-polarization field

**Fig. 1:**
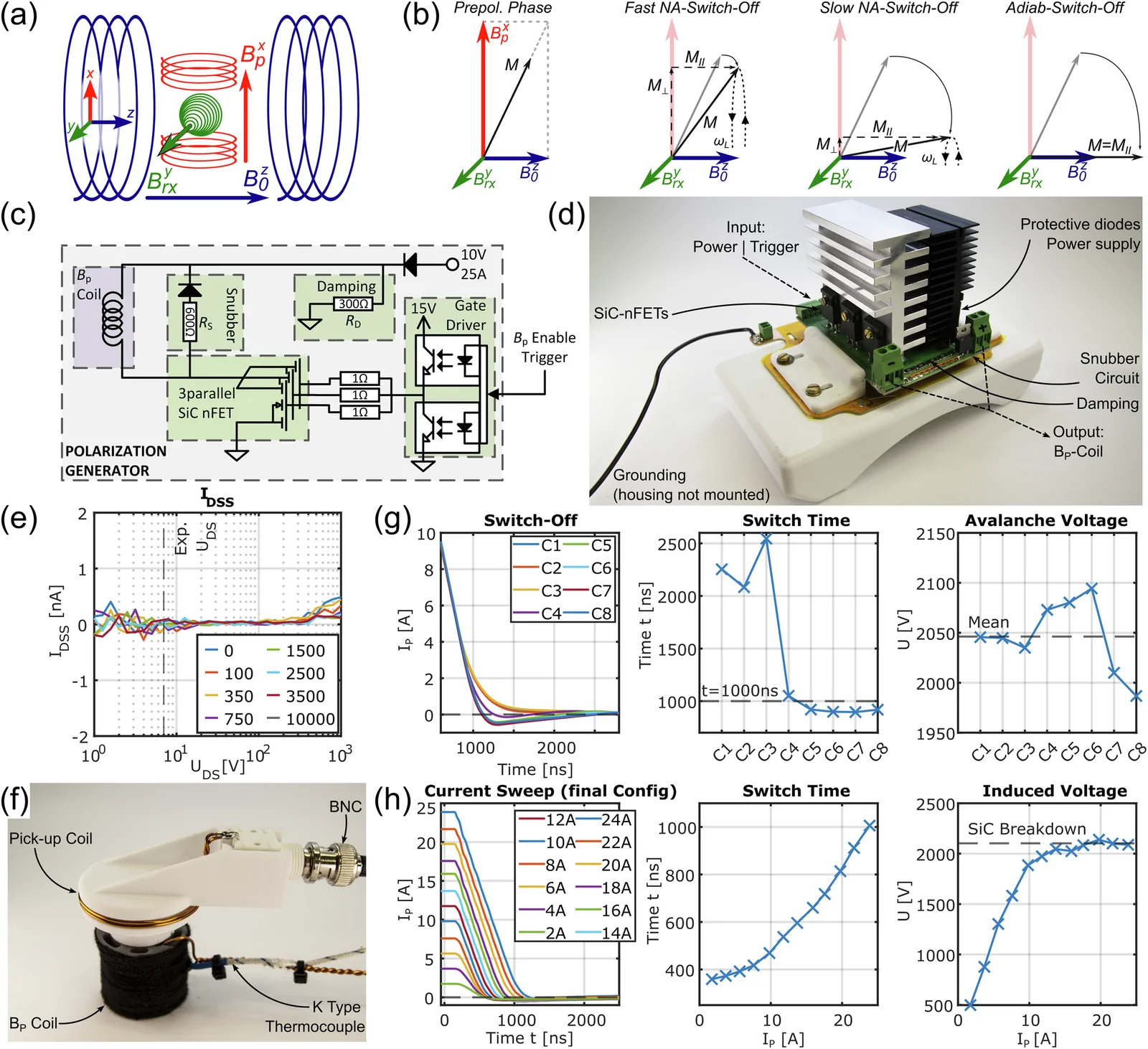
Overview of the non-adiabatic switch-off and the used hardware. **a** The three magnetic fields *B*_*p*_ (along the x-axis) of the pre-polarization coil, *B*_*rx*_ (along the y-axis, represented as an almost spherical winding) of the receiving coil, and *B*_*0*_ (along the z-axis) of the examined magnet are oriented at right angles. It is essential to ensure that *B*_*p*_ and *B*_*rx*_ are geometrically decoupled to protect the Rx electronics. **b** Schematic representation of the transition from the pre-polarization phase to the excited phase (in the case of non-adiabatic (NA) switch-off) or to the initial orientation along *B*_*0*_^*z*^ (in the case of adiabatic switch-off with increased polarization). **c** Block diagram illustrating the pre-polarization circuit, showcasing the three parallel SiC-nFETs, which guarantee full operational mode even if a single SiC-nFET fails (also known as the *n* + *1* principle). **d** Photograph of the assembled pre-polarization generator mounted on a 3D-printed base. The grounded top housing is not shown. **e** Measured reverse current *I*_*DSS*_ at applied drain-source-voltage *U*_*DS*_ after the aging test consisting of 0/100/…/10,000 measurement cycles. **f** Photograph of the designed low-cost pickup coil for characterizing the shutdown process. **g** Investigations of the shutdown process for different configurations of the pre-polarization circuit (set via resistor pair (*R*_*S*_ | *R*_*D*_) in Ω): C1 (300 | 225); C2 (300 | 300); C3 (300|open); C4 (400 | 300); C5 (500 | 225); C6 (500 | 300); C7 (600 | 225); C8 (600 | 300)). **h** Characterization of the final setup for different pre-polarization currents resulting in a dissipated power ramp of *∆P* < 55 kW/µs.

The core of the sensor is the excitation method using non-adiabatic switching-off of a strong pre-polarization field. The pre-polarization field ${B}_{p}^{x}$ (along the x-axis) is oriented perpendicular to the static magnetic field ${B}_{0}^{z}$ (along the z-axis) and to the Faraday Rx coil ${B}_{{Rx}}^{y}$ (along the y-axis, see Fig. 1a). In contrast to an adiabatic pre-polarization pulse, where the spin magnetization is realigned along ${B}_{0}$ after the completed pulse, a rapid non-adiabatic switch-off leaves a residual transverse magnetization ${M}_{\perp }$ if the following adiabatic condition is violated (see Fig. 1b)^42^:

1 $$\gamma \left|\vec{B}(t)\right|\gg \left|\frac{d}{{dt}}\angle \left(\vec{B}\left(t\right),\,\vec{{B}_{0}}\right)\right||\forall t$$

with $\vec{B}\left(t\right)=\vec{{B}_{p}}\left(t\right)+\vec{{B}_{0}}$ and γ being the gyromagnetic ratio. Note that the operator ∠ denotes the angle between $\vec{B}$ and $\vec{{B}_{0}}$ (see the Supplementary Information (SI) Chapter 3 for a more detailed analysis of Eq. (1)). The rotating remaining ${M}_{\perp }$ inductively generates a measurable voltage U in the Rx coil. The resulting ${M}_{\perp }$ after switch-off was numerically simulated using the Bloch equation^43^. In the ideal case of ${B}_{p}^{x}\gg {B}_{0}^{z}$ and a negligible switch-off (ramp) time ${t}_{R}\to 0$, ${M}_{\perp }$ is proportional to ${B}_{p}^{x}$, and since a Faraday detection coil is used *U* becomes $U \sim {B}_{p}^{x}{B}_{0}^{z}$, but is significantly reduced for slower switch-off times (see the detailed analysis in the SI Chapters 1 and 2). Based on simulations, the switch-off duration should be ${t}_{R}\le 1\mu {\mathrm{s}}$ for maximizing the available signal in typical (U)LFMRI devices.

In order to switch off ${B}_{p}^{x}$, a novel pre-polarization generator circuit (pre-polarization current ${I}_{p}\le 25{\rm{A}}$, coil inductance ${L}_{p}\approx 80\mu {\rm{H}}$) was designed to dissipate the energy of the polarization coil as quickly as possible (see Fig. 1c, d). The relationship $d{I}_{p}/{dt}=-{U}_{p}/{L}_{p}$ applies to the current ramp, with the coil voltage ${U}_{p}$. This implies that very high voltages in the kilovolt range (referred to as high-voltage HV) are required for the aforementioned coil inductance and current ${I}_{p}$ (which defines ${B}_{p}^{x}$). The HV for this sensor is provided by the intentionally triggered avalanche breakdown of HV SiC-nFETs with a maximum voltage rating ${U}_{{DS}\max }=1.7\mathrm{kV}$. Following the abrupt pre-polarization current switch-off, the coil’s self-induction causes the voltage across the SiC-nFET to rise until it transitions into a sudden avalanche breakdown (${U}_{{BD}}\approx 2.1\mathrm{kV}$), providing a linear discharge of the polarization coil.

It is a standard practice to test SiC-nFETs for their (repetitive) avalanche capability^46^, which helps to determine the shutdown behavior in the case of a device failure^47^ and to simplify circuits by avoiding extra protection components^48^. To the author’s knowledge, this sensor represents the first application in which a SiC-nFET is specifically operated in avalanche mode as the desired operating point, especially in this power and voltage class. Avalanche-induced crystal damage inside the SiC-nFET, which leads to an increased reverse current ${I}_{{DSS}}$, is particularly critical because even when switched off, ${B}_{p}^{x} > 0$ applies, directly distorting the sensor’s precision. ${I}_{{DSS}}$ was examined in an aging test with up to 10,000 avalanches of the used SiC-nFETs, where no damage was detected (shown in Fig. 1e, more details in SI Chapter 9).

The non-adiabatic switch-off can be precisely tuned using a snubber circuit to ensure rapid switching without oscillation or setting it to adiabatic pre-polarization^45^. The electrical measurement of this highly dynamic shutdown ($d{I}_{p}/{dt}=-26\mathrm{kA}/\mathrm{ms},{du}/{dt}=2.1\mathrm{kV}/150\mathrm{ns}$) typically involves the use of galvanically decoupled differential probes in combination with high-performance oscilloscopes. In order to ensure accessibility even in laboratories with limited resources, an easy to replicate solution has been used using an inductively coupled secondary coil in combination with a conventional oscilloscope (see Fig. 1f). As illustrated in Fig. 1g, the measured switch-off processes for ${I}_{p}=19{\rm{A}}$ are shown for different circuit configurations, resulting in a needed trade-off between the shutdown duration and the current undershoot. Bloch simulations involving the short undershoot showed no measurable influence on ${M}_{ \perp },$ so the configuration with the fastest possible switch-off was chosen (resulting in ${t}_{R}=896\mathrm{ns}$). The avalanche voltage is ${U}_{{BD}} > 2\mathrm{kV}$, which matches the expected voltage^49^. The switching behavior of this configuration is demonstrated at varying currents $1.7{\rm{A}}\le {I}_{p}\le 23.9{\rm{A}}$ in Fig. 1h, resulting in switch-off times from $359\mathrm{ns}\le {t}_{R}\le 1006\mathrm{ns}$. For ${I}_{p} < 12\mathrm{A}$, the stored energy is not sufficient for a SiC breakdown and will be dissipated in the snubber circuit only.

For the case ${B}_{0}^{z}\le 5\mathrm{mT}$ and this set configuration, the adiabatic condition (1) can be significantly violated for all tested ${B}_{p}^{x}\le 70\mathrm{mT}$. For LFMRI with ${B}_{0}^{z}\approx 50\mathrm{mT}$, the adiabatic condition is generally satisfied. In the numerical Bloch simulation, however, a very weak ${M}_{\perp }$ remains visible and is measurable if the sensor concept is designed accordingly (e.g., optimized Rx chain regarding bandwidth, gain, filtering, and optimized excitation dynamics regarding damping network, inductance, stored coil energy). The comparatively high Larmor frequency of approximately ${f}_{0}\approx 2\mathrm{MHz}$ is advantageous in this case, as the induced voltage in the Rx coil is proportional to ${f}_{0}$.

### Design of the sample, polarization coil, and receive coil for maximum sensitivity

**Fig. 2:**
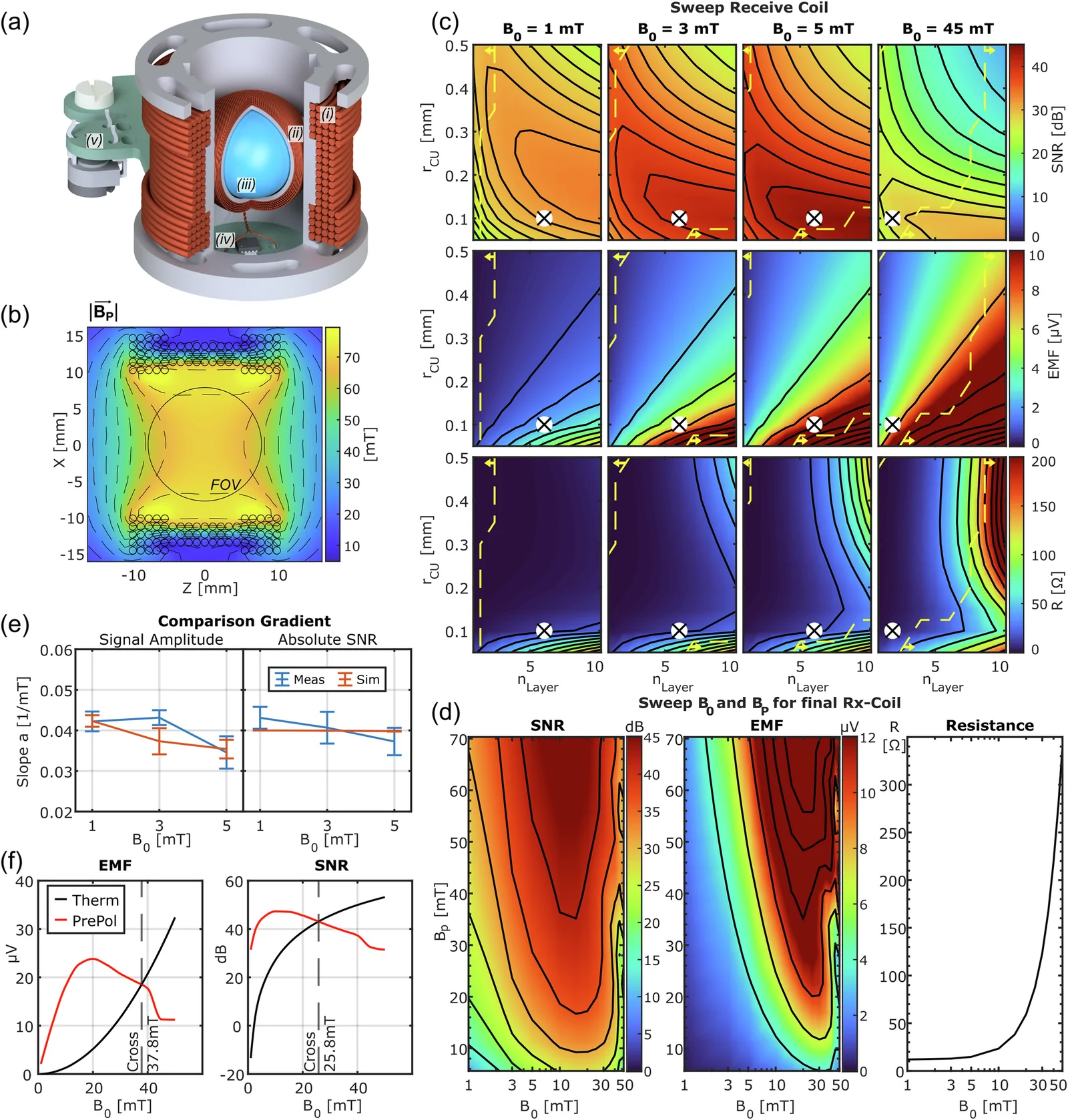
Design of the pre-polarization and Rx coil. **a** CAD rendering of the assembled frontend with (i) the outer pre-polarization coil, (ii) the inner Rx coil, (iii) the water sample located in an impregnated sample container, (iv) a closely positioned 3D Hall sensor, and (v) the frontend printed-circuit board (PCB). **b** Resulting *B*_*p*_-field from FEM simulation of the optimized pre-polarization coil (*I*_*p*_ = 20 A). **c** Optimization of the Rx coil for a simulated NMR signal at *B*_*0*_ = 1/3/5/45 mT (which were also experimentally measured in this work). The copper wire radius was varied in the range 0.1 mm≤*r*_*Cu*_ ≤ 0.5 mm. The number of winding layers was varied in the range 1 ≤ *n*_*Layer*_ ≤ 10. In conjunction with geometrical constraints, both parameters define the total number of axial turns. The calculated *SNR* (top), the calculated *EMF* (middle), and the coil-resistance *R* (bottom) are shown. The yellow boundary (line with arrows) indicates the range in which the Rx coil cannot be feasibly matched to *Z* = 200 Ω (optimum input impedance for LNA). For *B*_*0*_ ≤ 5mT the pre-polarization *B*_*p*_ = 61.9 mT (*t*_*R*_ = 918 ns) and for *B*_*0*_ = 45 mT the pre-polarization *B*_*p*_ = 70.3 mT (*t*_*R*_ = 1074 ns) was used. The white marker indicates the chosen Rx coil geometry. **d** Expected *SNR*, *EMF*, and *R* for the final design at different *B*_*0*_ and *B*_*p*_ combinations, where the measured *t*_*R*_ was used for each set *B*_*p*_. For easier comparison, the ULF configuration of the Rx coil is used for all fields. Note the logarithmic scaling of the x-axis. **e** A qualitative comparison between simulated and measured linear fit of *SNR*/*EMF*(*B*_*p*_) with *B*_*0*_ held constant. *a(B0)* corresponds to the linear gradient in the formulas *EMF*_*norm*_(*B*_*p*_) = *EMF*(*B*_*p*_)/*EMF*_*max*_ = a(*B*_*0*_)*B*_*p*_ + *OS*_*EMF*_, resp. *SNR*_*norm*_(*B*_*p*_) = *SNR*(*B*_*p*_)/*SNR*_*max*_ = a(*B*_*0*_)*B*_*p*_ + *OS*_*SNR*_. *OS*_*EMF*_/*OS*_*SNR*_ indicates the intercept. The whiskers indicate errors in the linear fit (although it is negligible for the simulated absolute SNR). The NMR spectra were normalized to the maximum value due to slight variations in Rx coil geometries between simulation and experiment (see Fig. 6a for measurement details and SI Chapter 6 for details). **f** Comparison of the simulated *EMF* and *SNR* between the excitation methods using non-adiabatic switch-off (*B*_*p*_ = 70mT) and conventional resonant RF excitation with polarization at *B*_0_ (same optimized Rx coil used). The observed anomaly at *B*_*0*_ ≈ 40mT in the switch-off method potentially results from inaccuracies in the measured *T*_2_* values, which are affected by *B*_*0*_ inhomogeneities at higher fields. For polarization at *B*_0_, a constant *T*_2_* was chosen for simplicity.

The NMR signal strength is predictably low at (U)LFMRI. To ensure that the sensor remains functional for the targeted wide range of $\Delta {B}_{0}=1-50\mathrm{mT}$, the Rx coil and pre-polarization coil were optimized using simulation. First, the sample volume was set, with a trade-off made between maximum volume (higher NMR signal due to more polarized spins) and the strongest possible pre-polarization field (limited to $L\le 80{\mu \mathrm{H}}$ and ${I}_{p}\le 25{\rm{A}}$). For ${B}_{0}\le 5\mathrm{mT}$, a volume $V=1.2\mathrm{ml}$, and for ${B}_{0}=45\mathrm{mT}$, a volume $V=1.5\mathrm{ml}$ was selected due to a smaller expected signal. Pure water was used as a sample, achieving the highest possible spin density and therefore the highest possible signal. In this proof of concept, a measurement volume of comparable size was selected to investigate the excitation method at different operating points. This can be reduced in future developments. The pre-polarization coil has been optimized to achieve the strongest possible field while keeping a reasonably high homogeneity under the given boundaries, resulting in a multilayer solenoid coil (see Fig. 2a, b). For an RF excitation with adiabatic pre-polarization (*hybrid mode*), the innermost layer was used as a Tx ${B}_{1}$-coil and the remaining layers for pre-polarization.

The shape of the Rx coil was optimized by solving the Bloch equations numerically and calculating the induced signal for various coil configurations (see the “Methods” section for details). Figure 2c shows the calculated electromotive force (*EMF*, voltage signal amplitude), *SNR*, and resistance *R* of the coil for varied geometries. The expected *EMF* increases significantly across all magnetic fields with more turns (many layers with small wire radius), which, however, results in higher resistances due to the total copper length and therefore limits the achievable *SNR*. The highly frequency-dependent skin-effect increases the resistance and reduces the *SNR* at ${B}_{0}=45\mathrm{mT}$ significantly below the value at ${B}_{0}=1\mathrm{mT}$. Based on these results, optimized ULF (${B}_{0}\le 5\mathrm{mT}$) and LF (${B}_{0}=45\mathrm{mT}$) configurations of the Rx coil were built (see the markers in Fig. 2c).

Using these configurations and the measured pre-polarization ramps, the expected *EMF*, *SNR* and *R* were calculated for $1{\mathrm{mT}}\le {B}_{0}\le 45\mathrm{mT}$ and $5\mathrm{mT}\le {B}_{p}\le 70\mathrm{mT}$ (see Fig. 2d). As with the initial Rx coil optimization, it is evident that pre-polarization is *SNR*-wise especially beneficial at low ${B}_{0}$. Due to impedance boundary constraints, the sensor may use the ULF configuration up to ${B}_{0}\le 10\mathrm{mT}$ and the LF configuration for higher fields. The linear increase of *SNR* and *EMF* over ${B}_{p}$ was compared for ${B}_{0}\le 5\mathrm{mT}$ with the normed measured results (see SI Chapter 6 for calculations). The simulated slope aligns closely with the measured slope, as demonstrated in Fig. 2e, highlighting the accurate physical modeling in the simulation for *SNR* estimation.

A simulated comparison of the *SNR* of this non-adiabatic excitation and a conventional RF excitation with polarization at ${B}_{0}$ reveals that non-adiabatic excitation is clearly advantageous up to a limiting magnetic field of ${B}_{0}=26\mathrm{mT}$ (${B}_{0}=38\mathrm{mT}$ when considering *EMF*), which is shown in Fig. 2f. Above this field, the conventional RF excitation generates higher *SNR*. This demonstrates that non-adiabatic excitation dominates at lower magnetic fields, in agreement with the prediction of Eq. (1). However, this limit applies to the designed hardware with the selected L and the chosen SiC-nFETs and can be increased in alternative configurations.

### Design of the receiver electronics

**Fig. 3:**
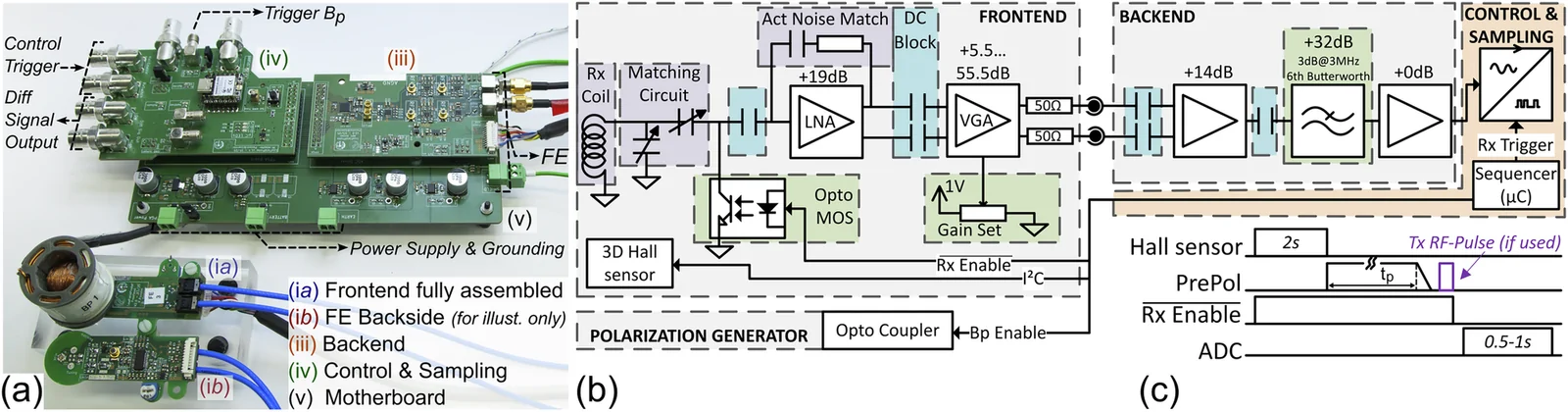
Overview of the receive electronics. **a** Photography of the entire Rx hardware as a modular architecture consisting of four PCBs. Note that the flipped frontend *(ib)* is shown only for illustration purposes and not used in the experiment. **b** Block diagram of the Rx electronics. **c** Measurement sequence used during the shown experiments. The Hall sensor could operate significantly faster than 2 s. Here it was switched on, configured, read out, and switched off during its time block with enough delay time to have it switched off before the strong *B*_*p*_ is switched on. Rx Enable is an active-low signal; therefore, a logical high switches the photocoupler (OptoMOS) on and grounds the LNA RF input.

A modular architecture was chosen to streamline debugging and upgrading if needed (shown in Fig. 3). The frontend, with mounted polarization coil and Rx coil (see Fig. 2a), followed by a low-noise amplifier (LNA), is positioned within the magnetic field. An additional Hall sensor in the center of the coil pair is used for rough Larmor frequency estimation to simplify spectrum analysis, as this estimated frequency determines the range for digital bandpass filtering^30^. The backend with an active anti-aliasing filter remains outside the magnetic field. In the shown experiments, the NMR signal is digitized using available commercial hardware, such as oscilloscopes.

### Measurement environments and examined magnets

**Fig. 4:**
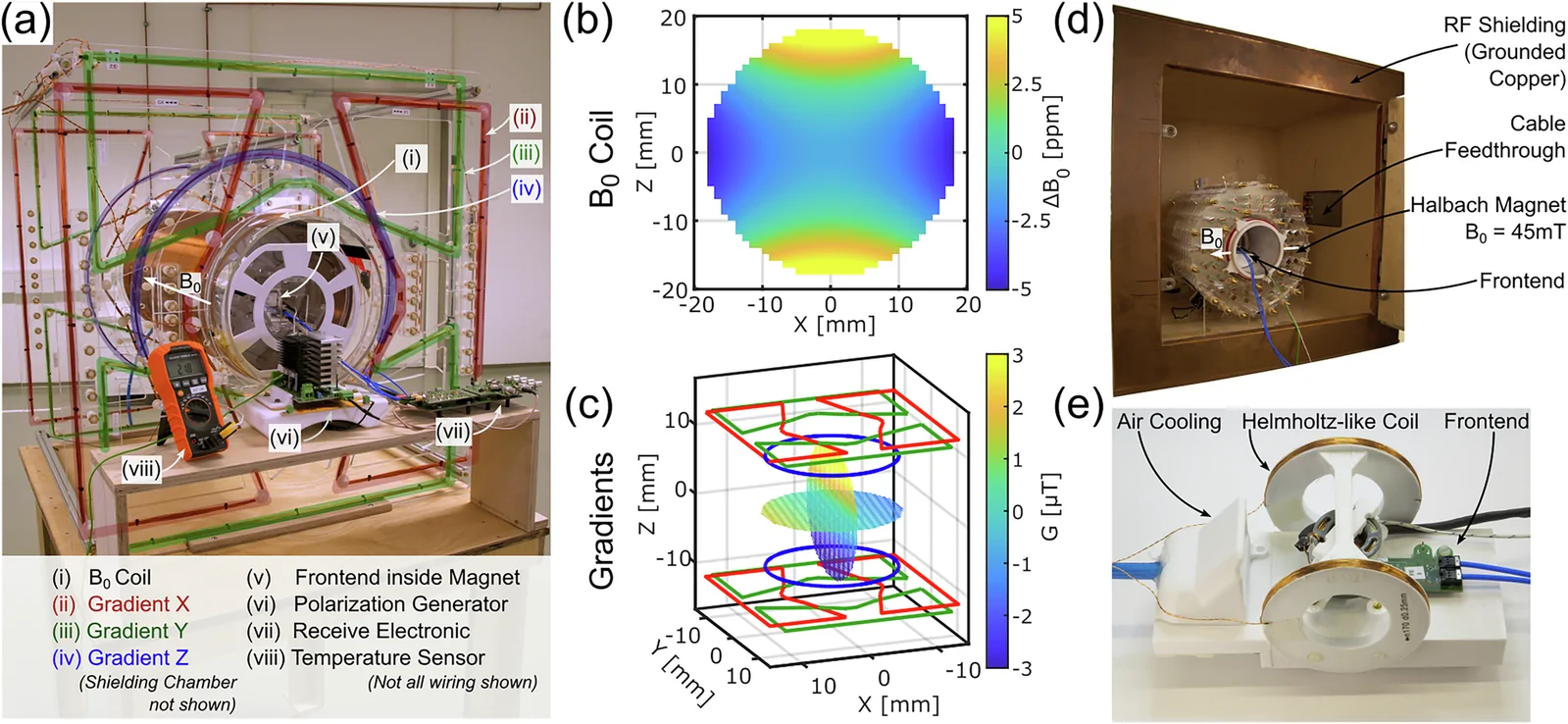
Overview of the magnetic fields investigated. **a** The used ULFMRI based on a homogeneity-optimized electromagnet for *B*_*0*_ and two planar X/Y-gradients and a Maxwell coil as the Z-gradient. An exemplary experimental setup is shown with the frontend positioned on a slider inside the magnet and the backend and pre-polarization generator placed outside the magnet. **b** Simulated *B*_*0*_ homogeneity Δ*B*_*0*_ in parts-per-million (ppm) of the ULFMRI magnet shown in (**a**). **c** Simulation of gradient efficiency at a gradient current of *I*_*G*_ = 1 A. Only the magnetic fields of the X and Z gradients are shown (Y is analogous to X, but rotated by 90°). The gradient coordinates are scaled by a factor of 1/25 for this plot. Note that the plot is rotated by 90° around the x-axis compared to (**a**) for illustrative purposes. **d** Photograph of the Halbach magnet inside the grounded RF shielding chamber. The frontend is positioned inside the magnet. Other electronics are not shown, but are positioned inside the RF shielding chamber during the experiments. The shielding may be designed in another way with, e.g., conductive cloths or copper meshes, since the frequencies in the MHz range can be shielded easily. **e** Photograph of the slider (usually positioned inside the Halbach magnet), accompanied by the Helmholtz-like offset coil for *B*_*0*_ field variation. The frontend is positioned in the isocenter of the offset coil, and the slider is positioned so that the sensor sample is approximately at the magnet isocenter.

The examined magnets are shown in Fig. 4. The ULFMRI based on a solenoid and gradient system was used for ${B}_{0}=1/3/5\mathrm{mT}$^50^. For ${B}_{0}=45\mathrm{mT}$, an unshimmed Halbach permanent magnet with an inhomogeneity of $\Delta B\approx 370\mathrm{ppm}$ (inside the sensor sample) and an integrated ${B}_{0}$ offset coil was used (see Fig. 4e), which, however, significantly decreases ${B}_{0}$ homogeneity^30^. The individual used frontends are listed in Table 1.

**Table 1:** Overview of the used setups

Setup			1 mT	3 mT	5 mT	45 mT	1 mT RF Tx
Rx Coil	*V* (Water)		1.2 ml	1.2 ml	1.2 ml	1.5 ml	1.2 ml
	*L*		811 µH	950 µH	771 µH	64 µH	811 µH
	*Q*		17.5	18.2	18.5	13.2	17.5
Polarization coil	*L*		80 µH	80 µH	81 µH	80 µH	49 µH
	*R*_*DC*_		153 mΩ	150 mΩ	156 mΩ	153 mΩ	122 mΩ
	*k*_*Bp*_		2.812 mT/A	2.812 mT/A	2.812 mT/A	2.812 mT/A	1.832 mT/A
RF Transmit	*B*_*1*_ Coil	*L*	x	x	x	x	6.9 µH
		*R*_*DC*_					46 mΩ
		*k*_*B1*_					0.684 mT/A
Rx Electronics	Gain (Overall)		110 dB_V_*(except* Fig. 5a*)*	110 dB_V_	105 dB_V_	102.5 dB_V_	107.5 dB_V_
	Sampling		500 ms 16 bit	500 ms 16 bit	500 ms 16 bit	1000 ms 12 bit	500 ms 16 bit

The Rx coils were wound manually, resulting in a random winding pattern and slightly different coupling inductances between the individual conductor loops. This leads to different inductances *L* and quality factors *Q* despite the same number of turns. There is a slight deviation when impedance matching, due to the discretized capacities on the frontend PCB and the strong inductive coupling with the polarization coil. For the pre-polarization coil, the inductivity *L*, the DC Resistance *R*_*DC*_, and the calculated pre-polarization efficiency *k*_*Bp*_ used in the relation *B*_*p*_ = *k*_*Bp*_*I*_*p*_ are shown. For the conventional RF excitation, the innermost layer of the pre-polarization coil was used.

### NMR measurements in ultra-low field for *B*_0_ ≤ 5 mT

**Fig. 5:**
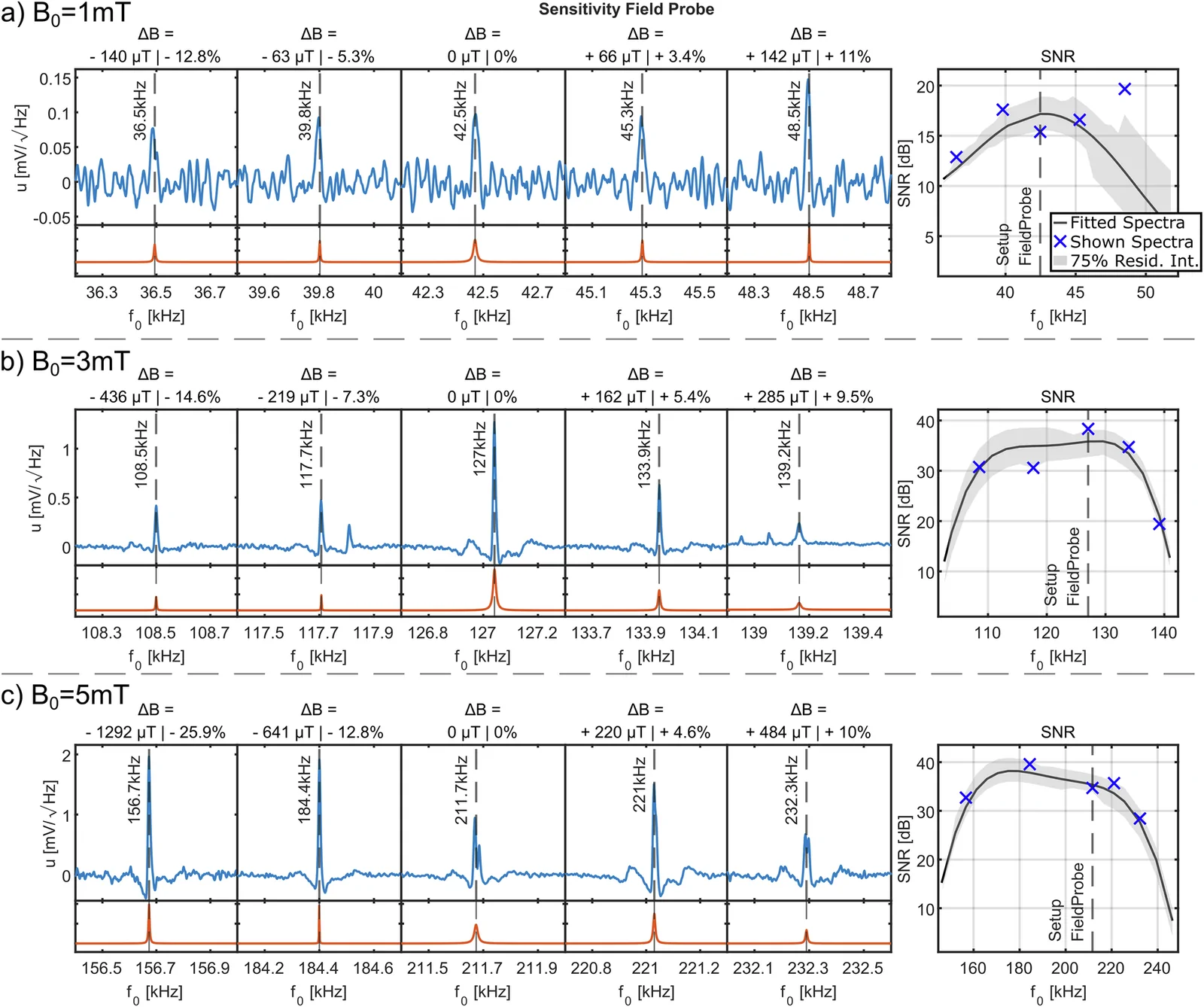
Exemplary phase-corrected NMR spectra (in blue) in the ULF for determining the sensing ranges of the assembled sensors. Beneath this, the fitted spectrum is shown (in orange, details in SI Chapter 5). For every *B*_*0*_ setup, three NMR spectra were measured and averaged. The voltage-related *SNR* is derived from the NMR spectra and shown on the right side of each. The measured SNRs were approximated using a fourth-order polynomial fit, with the shown uncertainty representing the central 75% interval of the residuals calculated using a rolling window of 8 points (75% Resid. Int.). The blue colored markers indicate the exemplary spectra shown on the left side. **a** Measured NMR spectra at *B*_*0*_ = 1 mT (*B*_*p*_ = 62 mT, *t*_*p*_ = 6 s). **b** Measured NMR spectra at *B*_*0*_ = 3mT (*B*_*p*_ = 61 mT, *t*_*p*_ = 6 s). The observable side peaks are not related to the NMR signal and are probably influenced by noise sources in the laboratory. **c** Measured NMR spectra at *B*_*0*_ = 5 mT (*B*_*p*_ = 60 mT, *t*_*p*_ = 6 s).

Figure 5 shows exemplary NMR spectra from the ULF magnet setup with the aim of demonstrating the functionality of the field probe over a wide range away from its theoretical ideal working point (related to the matching frequency). Figure 5a shows the results of averaging three measurements, achieving a balance between characterizing the sensor’s performance and minimizing current source-related ${B}_{0}$ drift (see the data handling in SI Chapter 5 and drift analysis in SI Chapter 11). However, to increase sensitivity, the measured NMR spectra can be fitted very robustly with a Lorentzian peak^51^. For ${B}_{0}=1\mathrm{mT}$, noise matching is imperfect due to the low Larmor frequency (and the associated large capacitances), which causes the LNA’s noise figure to increase by at least $\Delta {NF}=17 \%$ and thus raises the noise level in the measurement compared to ideal matching. Note that the receiver electronics’ set gain was significantly reduced to $G=105\mathrm{dB}$ for ${B}_{0}=1\mathrm{mT}$ due to additional unknown noise sources, resulting in railing of the electronics. This resulted in smaller signal amplitudes and additionally reduced *SNR* (e.g., due to increased quantization noise from sampling). Despite the noisy environment, the Larmor frequency can be detected without any issues using the measured spectra, resp. the fitted spectrum. According to this evaluation, the sensor operates over a comparatively wide working range ${\Delta f}_{{OP}}$ of ${\Delta f}_{{OP}}\in [-15|+50] \%$ for ${B}_{0}=1\mathrm{mT}$, ${\Delta f}_{{OP}}\in [-22|+15] \%$ for ${B}_{0}=1\mathrm{mT}$ and ${\Delta f}_{{OP}}\in [-25|+17] \%$ for ${B}_{0}=5\mathrm{mT}$ from the ideal matching frequency. The FWHM at ${B}_{0}=3/5\mathrm{mT}$ is well below $\Delta f < 10\mathrm{Hz}$ (resp.$\,\Delta f < 30\mathrm{Hz}$ for ${B}_{0}=1\mathrm{mT}$) resulting in an achievable sensitivity of ${f}_{{sens}} < 26\mathrm{ppm}$ (resp. ${f}_{{sens}} < 235\mathrm{ppm}$ for ${B}_{0}=1\mathrm{mT}$, see SI Chapter 12 for details), demonstrating the effectiveness of the build pre-polarization setup with minimizing transient disturbances^52^.

**Fig. 6:**
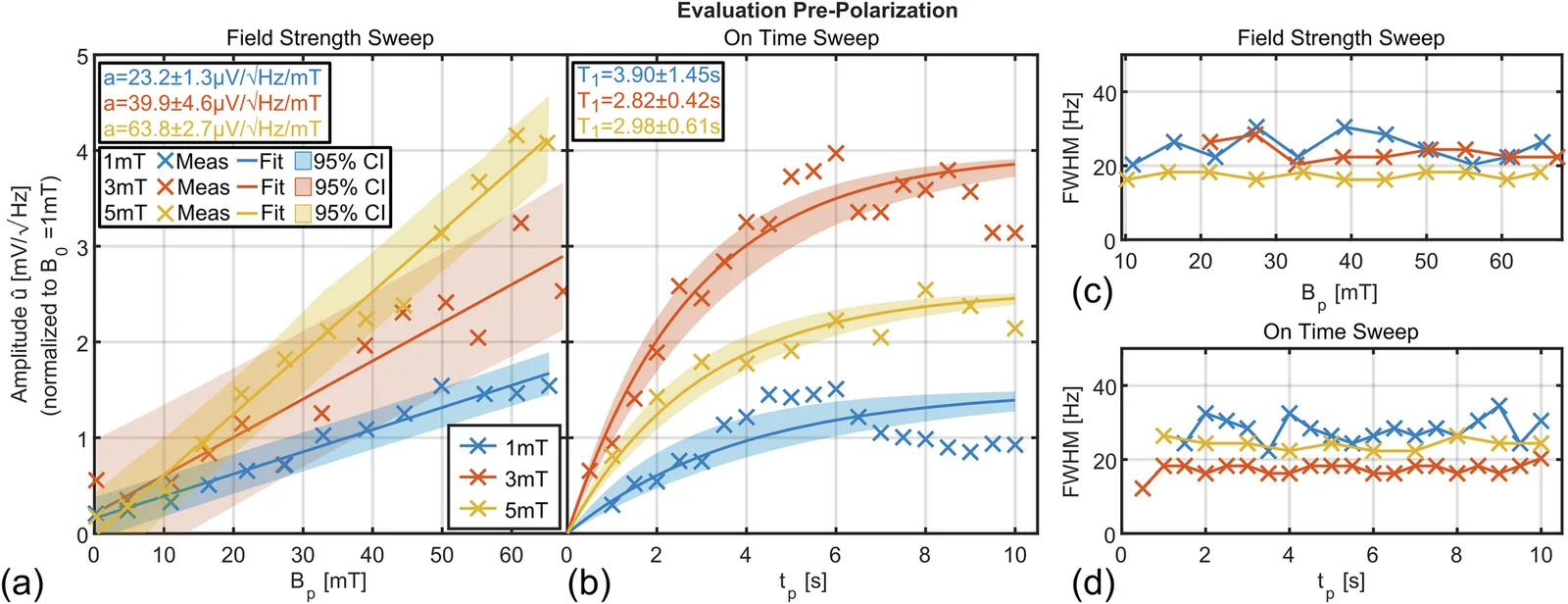
Investigation of the influence of the pre-polarization field strength *B*_*p*_ and duration *t*_*p*_ on the measured signal amplitude. To ensure comparability, all FID data were normalized to the total gain *G* = 110 dB set at *B*_*0*_ = 1 mT. For every configuration, one NMR spectrum was acquired (no averages). **a** Resulting signal amplitude for a swept pre-polarization field strength in the range 0mT<*B*_*p*_ < 70 mT (*t*_*p*_ = 6 s). The signal amplitude is linearly fitted with *û =* *aB*_*p*_ + *OS*_*u*_, resulting in the shown slopes *a*. The shown uncertainty represents the 95% confidence (95% CI) interval of the calculated slope. **b** Resulting signal amplitude for a swept pre-polarization duration in the range 0.5 s<*t*_*p*_ < 10 s (*B*_*p*_ = 61 mT). The signal ampli*t*ude is exponentially fitted with *û*(*t*) = *û*_*max*_(1 − exp(−*t*/*T*_*1*_)), resulting in the shown relaxation times *T*_*1*_. The shown uncertainty represents the 95% confidence interval (95% CI) of the calculated relaxation times *T*_1_. **c** Calculated FWHM for the acquired NMR spectra during the pre-polarization field strength sweep in (**a**). **d** Calculated FWHM for the acquired NMR spectra during the pre-polarization duration sweep in (**b**).

The dependence of the pre-polarization phase on the non-adiabatic switch-off was investigated by varying the field strength from $0\mathrm{mT}\le {B}_{p}\le 67.5\mathrm{mT}$ while keeping a constant pre-polarization time ${t}_{p}=6s$ (results shown in Fig. 6a) and in another experiment by varying the pre-polarization time $1s\le {t}_{p}\le 10s$ while keeping a constant pre-polarization field ${B}_{p}\approx 61\mathrm{mT}$ (results shown in Fig. 6b). In principle, ohmic losses in the polarization coil resulting in heating the water sample needs to be reduced by minimizing ${B}_{p}$ or duration ${t}_{p}$, with ${B}_{p}$ having a stronger influence (energy loss ${E}_{{loss}} \sim {{B}_{p}}^{2}{t}_{p}$). As ${B}_{p}$ increases, the measured amplitude increases linearly (as expected since ${M}_{\perp } \sim {B}_{p}$ for ${B}_{p}\gg {B}_{0}$). Already small pre-polarization fields ${B}_{p}\ge 10\mathrm{mT}$ are sufficient to measure an NMR spectrum. As ${t}_{p}$ increases, the signal amplitude follows an exponential behavior with the time constant being the NMR relaxation time ${T}_{1}$. The measured ${T}_{1}$ times are within the expected range and can be used for future and more precise simulation^27^. As seen, already short pre-polarization durations of ${t}_{p}\ge 2{\rm{s}}$ are sufficient to measure the Larmor frequency. Although the electronic performance of the pre-polarization generator varies significantly in both sweeps due to different working points, the determined FWHM is constant across the sweep range, highlighting the high spectral resolution of the sensor, even with a very efficient pre-polarization phase.

While the overall linear and exponential trends are clearly observed, the measured amplitudes exhibit noticeable scatter around the fitted curves. This variability is most likely caused by small fluctuations of the pre-polarization generator operating point, including minor current variations due to temperature-dependent changes of the excitation circuit and timing uncertainties of the excitation and receive chain (see SI Chapter 10 for a detailed analysis). These effects mainly influence the signal amplitude, whereas the measured FWHM remains nearly constant across the investigated parameter range, confirming the high spectral resolution and reliable NMR detection of the sensor.

The design can be tuned for different applications: Maximum signal intensity and therefore *SNR* (strong ${B}_{p}$, long ${t}_{p}$, long ${T}_{R}$) gives a narrow line width and high ${B}_{0}$ resolution. For higher temporal resolution with a strong signal, time constants should be minimized (strong ${B}_{p}$, short ${t}_{p}$, short ${T}_{R}$). Energy-efficient long-term drift measurements are possible with reduced ${B}_{p}$ (due to the quadratic dependence) while maintaining high signal with long pre-polarization times (weak ${B}_{p}$, long ${t}_{p}$, long ${T}_{R}$). Additionally, the designed sensor is sufficiently sensitive to resolve the weak field variations generated by gradient coils and enables characterization of gradient alignment, field drift, and first-order shimming performance (detailed experimental analysis provided in SI Chapter 7).

### Hybrid measurement with RF excitation in ultra-low field

**Fig. 7:**
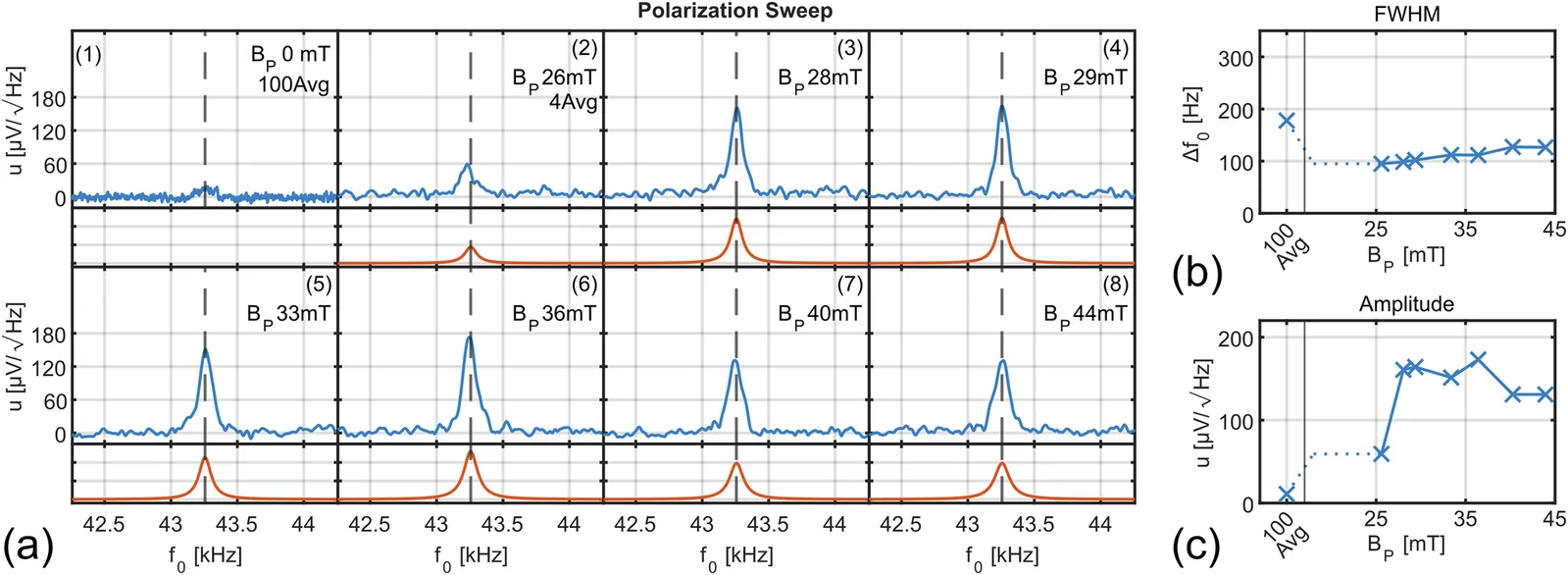
Hybrid excitation using a combination of pre-polarization with adiabatic switch-off and oscillating RF excitation at *B*_*0*_ = 1 mT. **a** Exemplary NMR spectra at different *B*_*p*_ (absolute value of the complex spectrum is shown). For the *B*_*p*_-sweep experiments, four measurements were averaged and filtered using an apodization filter (see SI Chapter 5). For the reference measurement (*B*_p_ = 0 mT), 100 measurements were averaged, and no apodization filter was applied, and no fit was performed. The fitted spectrum is shown in orange. Reliable detection of an NMR peak is difficult for *B*_p_ < 26 mT (and 4 averaged measurements). These measurements are presented in SI Chapter 8. **b** The FWHM derived from (**a**). **c** The amplitude *u* derived from (**a**).

Figure 7a illustrates the hybrid excitation, combining adiabatic pre-polarization followed by a conventional, on-resonant RF excitation at ${B}_{0}=1\mathrm{mT}$, representing a challenging test due to low Larmor frequency and small NMR signals. Increasing the pre-polarization field enhances the signal amplitude, with reliably detectable spectra obtained for ${B}_{p}\ge 26\mathrm{mT}$ using four averages. A slight increase in the FWHM with increasing pre-polarization field is observed (Fig. 7b). This broadening may originate from small additional dephasing effects resulting from transient field variations or residual inhomogeneities during the adiabatic switch-off of the pre-polarization field. However, this measurement shows that there is an optimal value for ${B}_{p}$. Since this contradicts expectations (since $u \sim {B}_{0}{B}_{p}$), it remains to be investigated whether, for example, switching off higher ${I}_{p}$ causes high-frequency interference signals to couple into the Tx coil (which, due to its design, is well inductively coupled), thereby varying the resonant Tx pulse. But the successful operation demonstrates the applicability of the sensor principle also at higher magnetic fields, where the polarization is already intrinsically higher.

### NMR measurements in low field at *B*_0_ = 45 mT

**Fig. 8:**
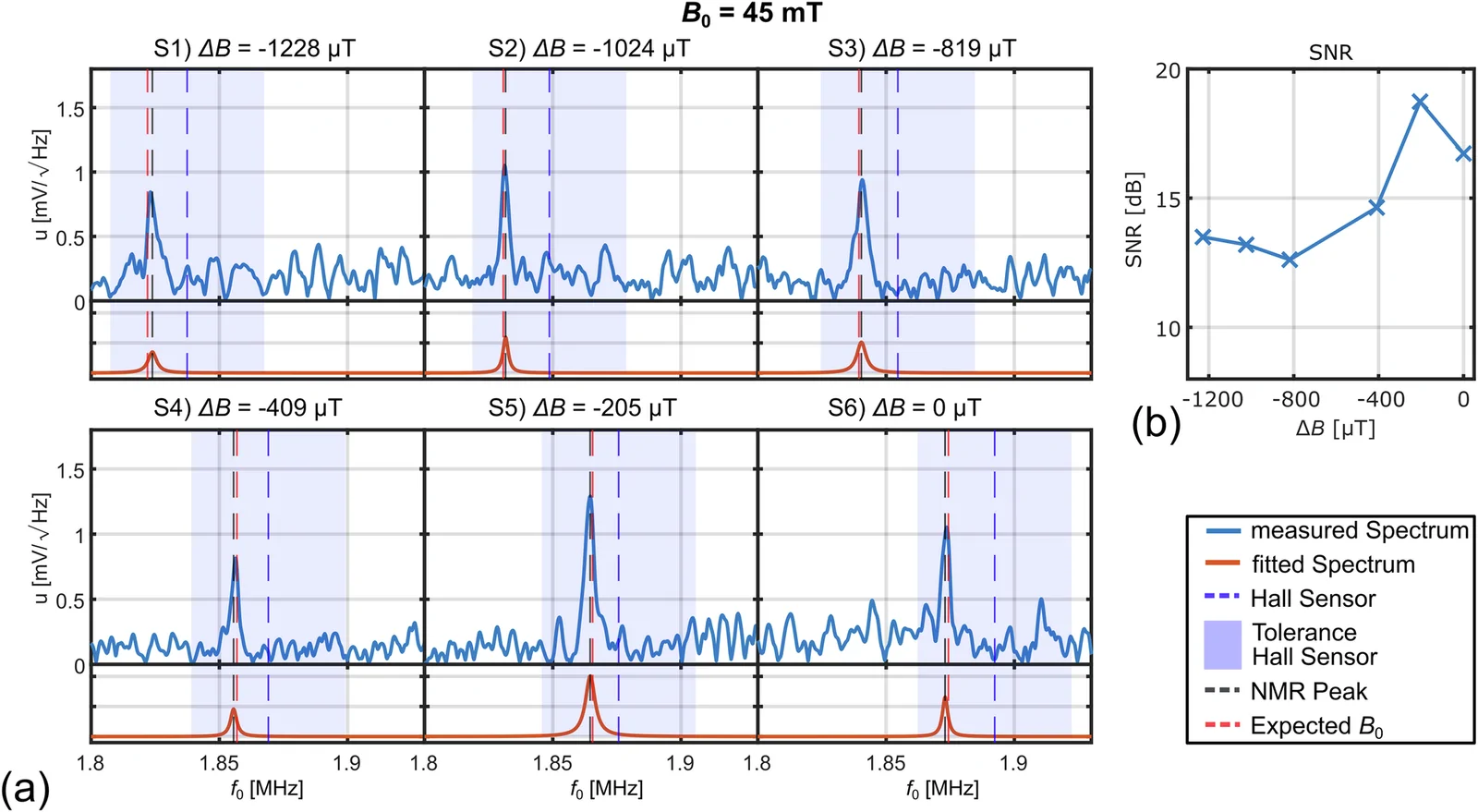
Measured NMR spectra for an unshimmed Halbach magnet at *B*_*0*_ = 45 mT. The field probe was tuned to *f* = 1.852 MHz and pre-polarization was set to *B*_*p*_ = 70.3 mT for *t*_*p*_ = 6 s. **a** Exemplary selection of acquired NMR spectra (absolute value of the complex spectra is shown) in a varying offset field of −1.228 mT < ∆*B*_0_ < 0 mT in six steps. The absolute value of the complex spectra is shown in blue. The fitted spectra are shown in orange (see SI Chapter 5 for details). Data were not averaged due to a slight dynamic magnetic field drift. The vertical lines correspond to the measured value from the embedded Hall sensor (taking into account the tolerances of the used sensor as well as the field offset due to a shifted position), to the detected Larmor frequency, and to the expected Larmor frequency due to the set of the additional offset coil (see Fig. 4e). **b** SNR of the measurements derived from (**a**).

In Fig. 8, the measured NMR spectrum with the highest *SNR* at each operating point inside an unshimmed Halbach magnet at ${B}_{0}\approx 45\mathrm{mT}$ is shown. Three measurements were taken for each operating point covering a range from $\Delta {B}_{0}=-1228{\mu \mathrm{T}}$ to $\Delta {B}_{0}=0\mu {\rm{T}}$. The complete set of acquired spectra is provided in SI Chapter 10. The expected ${f}_{0}$ is estimated from the known offset coil constant. For each operating point, at least one successful measurement was obtained, exhibiting a mean relaxation time of ${T}_{2}^{* }\approx 0.12\mathrm{ms}$ (averaged $FWHM=4028\rm{Hz}$) within the investigated volume. This is consistent with the estimated magnet inhomogeneity from a previous Hall sensor-based mapping, including the additional inhomogeneity introduced by the offset coil^30^.

The results from consecutive measurements at the same operational point demonstrate a considerable drift in the detected magnetic field, reaching a frequency variation of up to hundreds of Hertz. Due to the significant frequency drift, averaging the spectra from repeated measurements is not recommended as it broadens the measured peak and consequently reduces *SNR*. Furthermore, averaging is not necessary, since the *SNR* of individual spectra is already sufficient for peak detection, and robust spectral fitting from single acquisitions is possible. Due to the very short ${T}_{2}^{* }$, the accuracy of the ${B}_{0}$ mapping is limited in this unshimmed case, but is still sufficient for an initial shimming and significantly more precise than Hall sensors (here ${\Delta }_{{NMR}}\approx 0.1 \%$ compared to the build in Hall sensor $\left|{\Delta }_{{Hall}}\right|\le 2.5 \%$, see SI Chapter 12 for details). Significantly longer ${T}_{2}^{* }$ are expected for shimmed magnets. Since the measured NMR peak frequency corresponds exactly to the set offset field, it can be assumed that the influence of the switch-off mode is negligible on the sensor accuracy.

Some measurements did not yield a detectable NMR spectrum (see measured spectra in SI Chapter 10). This behavior originates most likely from the highly dynamic excitation process rather than from the sensor concept itself. The pre-polarization pulse is terminated by intentionally operating three parallel SiC-nFETs in avalanche mode to rapidly discharge the excitation coil. Although this approach enables the required sub-microsecond current decay, avalanche switching is influenced by stochastic device behavior as well as by parasitic circuit elements^53–55^. Consequently, the exact temporal evolution of the discharge waveform varies slightly between individual excitation cycles. While these electrical variations are pretty small, the magnetization dynamics become exceptionally sensitive once the excitation approaches the adiabatic limit (resp. when Eq. (1) is almost fulfilled, see SI Chapter 3). Under these conditions, nanosecond-scale changes in the avalanche termination, the resulting current waveform, or the interaction with the snubber and damping networks modify the magnetization trajectory during the final phase of the excitation. Extended Bloch simulations based on experimentally measured current waveforms demonstrate that even minor variations of the avalanche timing can reduce the generated transverse magnetization significantly and, in extreme cases, almost completely suppress it. In these cases, no measurable FID is generated despite otherwise identical experimental conditions. A detailed analysis of the avalanche dynamics and the corresponding Bloch simulations is provided in SI Chapter 10

A second contribution arises from the finite delay between the end of the excitation and the start of signal acquisition, which was measured to vary between 2 µs and 26 µs (mean 10.37 µs). Because the generated FID is very short in highly inhomogeneous magnetic fields, even small timing variations further reduce the detectable signal amplitude. Consequently, measurements that already produce only weak transverse magnetization due to avalanche-induced waveform variations may fall below the detection threshold (more details can be found in SI Chapter 10).

## Discussion

In this work, we have developed a functional, cost-effective, low-field probe capable of rapidly and autonomously examining the magnetic field of an unknown (U)LFMRI-magnet in the range $1\le {B}_{0}\le 50\mathrm{mT}$. The central component is the newly designed pre-polarization generator, which enables rapid non-adiabatic switch-off of the pre-polarization field, leaving a transverse magnetization ${M}_{\perp }$ without any additional on-resonant excitation. The key novelty here was the first use of a high-voltage SiC-nFET at a previously unused dedicated operating point, where avalanche breakdown was purposefully triggered at ${U}_{{BD}}\approx 2.1\mathrm{kV}$, resulting in very strong current ramps of $\Delta i=-26.4\mathrm{kA}/\mathrm{ms}$ ($\Delta B=-70{\rm{T}}/\mathrm{ms}$). Non-adiabatic switch-off has been proven to be an elegant solution for use in magnetic field sensors and was already successfully investigated in earth magnetic field-based NMR^44,45^, or close to zero-field for physical experiments^56^. Pre-polarization is beneficial in (U)LF NMR because it increases spin polarization and thus the signal level, relaxing requirements on the receive electronics. In MRI systems with ${B}_{0}\ge 40\mathrm{mT}$, however, this advantage becomes less pronounced. In these fields, the presented approach retains an important benefit with the frequency-independent excitation. Conventional NMR field probes determine the Larmor frequency by sweeping the transmit frequency until resonance is reached, using either FID experiments^37,38^ or continuous-wave schemes^36^, and typically requiring repeated measurements and matching-network adjustments. This procedure can be time-consuming, particularly for weak signals that require averaging. The non-adiabatic switch-off concept removes this requirement, since the signal scales additionally with ${B}_{p}$. However, satisfying the non-adiabatic condition becomes increasingly difficult at higher fields. Resonant excitation enables smaller hardware design and sample volumes (e.g., $V\le 0.1\mathrm{ml}$ at ${B}_{0}=77\mathrm{mT}$ in ref. ^38^, or at ${B}_{0}=290\mathrm{mT}$ in ref. ^57^, resp. $V\,\ll\, 1{\mu \mathrm{l}}$ at ${B}_{0}=14{\rm{T}}$ in ref. ^58^) and faster measurements since only short transmit pulses are required. However, this approach requires additional RF hardware such as tunable oscillators or signal generators. Non-adiabatic excitation simplifies system implementation but requires careful sensor head design and windowing of the initial FID samples to suppress switch-off transients, reducing the effective *SNR*.

Overall, in the range $1\mathrm{mT}\le {B}_{0}\le 50\mathrm{mT}$, non-adiabatic excitation is advantageous at lower fields due to its frequency-independent operation and higher polarization levels, whereas resonant excitation becomes increasingly favorable at higher fields because of larger signals and simpler frequency determination^36,37^. As demonstrated by simulations, the crossover ${B}_{0}$ at which resonant excitation should yield a stronger signal than non-adiabatic excitation occurs around ${B}_{0}=26\mathrm{mT}$. The achievable update rate in both systems reflects the relation between pre-polarization duration and the averaging required for resonant excitation to achieve similar SNR: resonant methods may require ${n}_{A}$ averages (since ${SNR} \sim \sqrt{{n}_{A}}$), whereas a single non-adiabatic excitation can achieve comparable sensitivity in shorter times (if ${B}_{p}\gg {B}_{0}$).

Heating of the NMR sample by the pre-polarization coil can slightly reduce sensitivity by lowering spin polarization and increasing thermal noise. Although frequency shifts due to chemical shifts are negligible at low fields, ${T}_{2}$ decreases with increasing temperature, reducing the accuracy of Larmor frequency determination^59^. In the present measurements, the maximum temperature increase was $\Delta T\le 4{\rm{K}}$, and no additional sensor-related frequency drift beyond the intrinsic magnetic field drift was observed.

In principle, any high-voltage laboratory supply could generate the required pre-polarization current ramps. However, such systems require extensive safety measures (e.g., insulation monitoring and interlocks), have to be implemented by trained high-voltage personnel, and are costly. In contrast, the proposed approach provides a simple, low-cost solution by generating the high voltage locally within the transistor package, requiring only basic touch protection. The stored coil energy (${W}_{P} < 25\mathrm{mJ}$) is non-hazardous, being comparable to a typical human-body electrostatic discharge^60^. Furthermore, the pre-polarization generator can be characterized using galvanically isolated pickup loops and inexpensive oscilloscopes. With the implemented fail-safe design, which was achieved through parallelization, the selection of appropriate SiC-nFETs, and the aging tests, it is safe to assume that more than 10,000 measurement cycles per transistor are easily possible. Possible damage is detectable in further developments by implementing a reverse current measurement. Even faster switch-offs would be possible by integrating novel developed 3.3 kV-SiC-nFETs in the build PCB, which enables even higher ${B}_{0}$ to be measured.

Accurate matching with a high Q factor of the Rx coil is not required by design, as it is only necessary for rough noise matching with the first LNA. Currently, the frontends are not exchangeable, and the frontend must be rematched or replaced for different field strengths. To minimize expenses, the frontend was designed to use as few components as possible so multiple ones can be built. Additionally, the RF path was designed to allow rematching even when fully assembled. Future developments could automate the matching adjustments with electronically adjustable capacitors, enabling a single frontend to cover a broader field range.

For HFMRI, NMR field probes are used for improving image quality by measuring the actual time-varying magnetic field (influenced by imperfect gradients, eddy currents, etc.) during the MRI examination^61^. The field probe presented here extends this function to (U)LFMRI: The drift of Halbach magnets, for example, is a known problem that was also recorded by our experiments^62^. In our unshimmed magnet, the drift was exceptionally high, which likely prohibits any MR imaging and may be ascribed to temperature fluctuations resulting from ohmic losses^63^. With this field probe, however, it can be solved by active shimming using the sensor output as a controller input (similar setup to that in ref. ^38^). The same is possible for the well-known current drift in actively driven electromagnets^24,26^. Additional insights into the magnetic field characteristics are possible by automatically moving the sensor within the magnet^30,64^.

Water was used to maximize the signal, and the engineered impregnation of the sample container successfully prevented drying. Future developments should reduce the long repetition times by using CuSO*₄*-doped water or solid samples with shorter ${T}_{1}$^37^. Alternatively, fluorinated samples could be employed, as they provide a similar gyromagnetic ratio while remaining invisible in proton MRI. The achieved *SNR* also permits further reduction of the sample volume. In the present configuration, the frequency resolution is fundamentally limited by the sample ${T}_{2}$ to the Hz range. Higher resolution would require alternative NMR-based approaches, such as RASER^65^.

In summary, the presented results, especially in the field of ${B}_{0}=45\mathrm{mT}$, should be regarded as a proof-of-principle demonstration of controlled avalanche operation in novel high-voltage SiC transistors for NMR excitation. This approach enabled non-adiabatic excitation fields exceeding the previous state of the art by about three orders of magnitude compared with excitation in the Earth’s magnetic field^44,45^. Furthermore, the results demonstrate that the commonly applied adiabaticity criterion described by Tannús and Garwood is not an absolute limitation, but can be substantially exceeded under controlled conditions^42^. To the best of our knowledge, this represents the first experimental demonstration of such a regime, as well as the first application of the controlled avalanche breakdown in sensing in general. The sensor concept was evaluated in two application-relevant LFMRI regimes, namely electromagnet-based ULF systems and permanent-magnet LF systems. While intermediate fields would further confirm the simulated sensor performance, they were not investigated as they currently have limited relevance for MRI applications. At the same time, precise control of the avalanche dynamics is essential. The circuit architecture presented here provides an initial level of control, while improved avalanche characterization and more sophisticated switching concepts may be investigated in future work. The observed variable measurement performance at higher fields primarily reflects limitations of the pre-polarization generator rather than the sensing concept itself. Notably, despite the extreme voltage transients and associated electromagnetic stress on the low-voltage electronics, reliable operation was maintained, demonstrating the effectiveness of the implemented stabilization measures. Further applications of the presented system may include characterizing various NMR magnets, such as benchtop systems^66^, or calibrating other integrated sensors, such as Hall or magnetoresistive sensors^67^. Particular emphasis during design was placed on ensuring that this hardware is as easy to replicate and upgradable as possible – even with constrained resources^5,68^, resulting in a modular electronic architecture and the avoidance of any special tools, parts, or expertise. All hardware and software are available as open hardware, enabling direct improvements to all LFMRI scanners already built and installed, as well as the development of new hardware and sequences.

## Methods

### Simulation and mechanical design of the pre-polarization coil

The pre-polarization field strength and possible switch-off times result in a trade-off of the winding pattern since a high number of windings creates a strong field, but also high inductance. With the design limitations of the pre-polarization generator and especially with the limited and non-adjustable avalanche voltage, the inductance was chosen to be of higher importance. One important additional geometrical constraint is the replaceability of the inner Rx coil, which facilitates reliable sample handling and allows the sample to be refilled or replaced, particularly after extended periods of non-operation.

Different variations of the polarization coil have been calculated using FEM (implemented in the open-source software FEMM^69^), and the best option was chosen. The final polarization coil is a multilayer solenoid coil with an efficiency of ${k}_{{Bp}}=2.812\mathrm{mT/A}$, $L=80\mu {\rm{H}}$, using an enameled copper wire with a radius of ${r}_{{Cu}}=0.5\mathrm{mm}$. The simulated inhomogeneity inside the sample is $\Delta {B}_{p}=18.9 \%$. In the axial direction, the coil has 19 windings and 3 layers, with the outer 5 windings on the inside and outside each having one more winding (Fig. 2a). By following a prescribed winding specification, it is possible to wind the coil uniformly and avoid gaps between the layers. The connecting wires are approximately 30 cm long twisted wire. The coil former is 3D-printed using Stereolithography (SLA), and due to the thermosetting plastic, it is particularly temperature-stable. Another printing technology that uses a suitable temperature-resistant material can also be used. To ensure the stability of the windings during the winding process, they were affixed with superglue.

The pre-polarization coil was modified for the hybrid mode with adiabatic pre-polarization and RF Tx pulses. The bottom copper layer is separated as a ${B}_{1}$-coil with efficiency ${k}_{{TX}}=0.684\mathrm{mT/A}$ and matched to $Z\approx 16\Omega$ at $f=43.26\mathrm{kHz}$ using a single-sided matching network. Due to the low frequency, ideal 50Ω matching is not necessary. The remaining outer layers collectively constitute the pre-polarization coil with efficiency ${k}_{{BP}}=1.832\mathrm{mT}/{\rm{A}}$. For both sub-coils, enameled copper wire with ${r}_{{Cu}}=0.5\mathrm{mm}$ was chosen.

### Simulation and mechanical design of the receive coil

In order to optimize the receive coil design, the Bloch equation was solved numerically in a MATLAB script to calculate the transversal magnetization of the sample^43^. A steady-state magnetization before the switch-off was assumed (quasi-infinite pre-polarization). Using the well-proven reciprocity principle, the induced voltage can be calculated, if the *B*_*1*_-characteristics of the Rx coil are known for a unit current prior^70^ (see SI Chapter 2 for details). The *B*_*1*_-characteristics of the Rx coil were calculated using Biot-Savart’s Law^71^. The basic geometry consists of a multi-layered spherical-like coil into which the spherical sample container is embedded. This optimization was recalculated for all configurations of *B*_*0*_, sample volume V, ${B}_{p}$, switching time ${t}_{p}$, and all Rx coil parameters (number of layers ${n}_{{Layer}}$, number of windings n and wire radius ${r}_{{Cu}}$).

The resistance was calculated using the DC resistance of the overall copper length and by considering the frequency-dependent skin^72^ and proximity effects^73^. This overall resistance was used to calculate the Johnson-Nyquist noise (using the real bandwidth of the Rx electronics). Further noise sources of the Rx electronics and environmental noise were neglected for simplicity (see SI Chapter 4 for details). The achievable *SNR* was estimated using this noise level and the calculated induced voltage signal and is therefore overestimated significantly (since noise will be higher in reality). An additional boundary condition involves the possibility to match the Rx coil to an impedance of Z=200Ω (optimal input impedance with minimum noise figure of the used LNA). Based on the simulation results, the following parameter combinations were selected for the Rx coils: For ${B}_{0}\le 5\mathrm{mT}$ with ${r}_{{Cu}}=0.1\mathrm{mm}$ and ${n}_{{Layer}}=6$ (ULF configuration) and for ${B}_{0}=45\mathrm{mT}$ ${r}_{{Cu}}=0.1\mathrm{mm}$ and ${n}_{{Layer}}=2$ (LF configuration).

Manufacturing the Rx coil is relatively complex, as the turns should be positioned as close as possible to the spherical sample. The winding process was done carefully, with each turn stabilized using activated superglue. A self-developed electronic counter was used to track the number of achieved turns. Due to the rough surface of the first layer of the printed container and the resulting valleys between the subsequent layers’ turns, as well as the superglue fixation, a fairly regular winding pattern emerges. However, the wires do not fit together perfectly, and glue also builds up, resulting in fewer turns being achieved. For instance, the ULF configuration required 570 turns, but only 360 could be practically achieved. According to the simulation (see Fig. 1c), this should only have a minor impact on the achievable SNR. For noise matching, a balanced matching circuit consisting of two matching and one tuning capacitor was used (each consisting of parallel fixed SMD and adjustable capacitors). A VNA (${Z}_{{in}}=50\Omega$, ZNLE3, Rohde&Schwarz, Munich, Germany) can be connected via a switchable coaxial connector (Micro-Switch, Amphenol, Danbury, USA), while simultaneously disconnecting the LNA. This allowed the matching network to be adjusted even on fully assembled PCBs. The matching does not need to be particularly good as the LNA has a relatively constant noise figure around its optimum (for example, at ${B}_{0}=3\mathrm{mT}$, the set impedance was $Z\approx 136\Omega$ at Larmor frequency).

### Sample container design

The spherical sample container was 3D-printed using a FDM printer from PETG with a nozzle diameter of ${d}_{N}=0.1\mathrm{mm}$ and was double-sealed with a polymer impregnation (DIAMANT Polymer GmbH, Moenchengladbach, Germany) in an immersion bath. The wall thickness is ${d}_{w}=0.6\mathrm{mm}$. The internal volume is a sphere with a volume of $V=1.2\mathrm{ml}$ (ULF configuration) or $V=1.5\mathrm{ml}$ (LF configuration). The container is filled with water as a sample using a syringe. Afterward, the hole was sealed by melting it using a soldering iron. The impregnation was tested for three days in a vacuum chamber at a pressure $p=250\mathrm{mbar}$ (water boiling point $T=70^\circ C$, room temperature $T=25^\circ {\rm{C}}$). This experiment shows no measurable water loss.

### Receive electronics

According to the Friis formula, the noise characteristics of the first amplifier are crucial. The single-ended LNA AD8331 (Analog Devices, Norwood, USA), typically used in ultrasound imaging, was used with a gain of $G=19\mathrm{dB}$ and ${NF}=1\mathrm{dB}$ (${Z}_{{in}}=200\Omega$). A subsequent variable gain amplifier (VGA, same package) allows manual fine adjustment of the overall Rx gain by feeding an adjustable voltage (manually set by a voltage divider with a potentiometer, see Fig. 3). The VGA output is differential, enabling a more noise-resistant signal transport outside the magnet. An active sixth-order Butterworth anti-aliasing filter with a cut-off frequency of ${f}_{3\mathrm{dB}}=3\mathrm{MHz}$ is placed on the backend PCB, which is positioned outside the magnetic field. The filter is designed single-ended for easier manufacturing (fewer tolerance-affected components). A differential-to-single-ended converter is positioned in front of the filter. The voltage-related total amplification of the input voltage signal ranges from $G=72\mathrm{dB}$ (gain 3980) to $G=120\mathrm{dB}$ (gain 1,000,000), depending on the VGA setting. The VGA gain is optimized to prevent noise-induced railing in subsequent amplifiers. A photocoupler (TLP3431, Toshiba Electronics, Tokyo, Japan) short-circuits the Rx coil during the pre-polarization phase. The circuit on the frontend was evaluated using the software Advanced Design System (Keysight Technologies, Santa Rosa, USA) to ensure circuit stability in noisy environments. The filter was simulated using the software LTSpice (Analog Devices). The output of the VGA was stabilized with a series resistor R=50Ω, and the output of the filter with $R=1{\rm{k}}{\Omega }$ against capacitive loads (like coaxial wires). For the measurements in this work, ${B}_{0}\le 5\mathrm{mT}$ was sampled with a PXI-6363 card (${t}_{s}=0.5{\rm{s}}$, $Q=16\mathrm{bit}$, ${f}_{s}=2\mathrm{MSps}$, National Instruments, Austin, USA) and ${B}_{0}=45\mathrm{mT}$ was sampled with a HDO6104-MS oscilloscope (${t}_{s}=1\mathrm{s}$, $Q=12\mathrm{bit}$, ${f}_{s}=10\mathrm{MSps}$, Teledyne LeCroy, Thousand Oaks, USA). Any other oscilloscope with a resolution of $Q\ge 12\mathrm{bit}$, a sampling rate ${f}_{S}\ge 5\mathrm{MSps}$ and a memory $m\ge 2.5\mathrm{MS}$ is sufficient. The signal is then evaluated in post-processing on a PC using a MATLAB (The MathWorks Inc., Natick, USA) script. An alternative option is available in the design files, which includes an integrated analog-to-digital converter (ADC, LTC2225, Analog Devices, $Q=12\mathrm{bit}$, ${f}_{s}=10\mathrm{MSps}$) and evaluation with an FPGA (iCE40UP5K, Lattice Semiconductor, Portland, USA). Please note that this option requires some experience in embedded coding. An additional 3D Hall sensor ALS31300 (Allegro Microsystems, Manchester, USA) with a measuring range of ${B}_{X/Y/Z}=\pm 50\mathrm{mT}$ has been installed in the center of the pre-polarization coil and is used in particular for rough Larmor frequency estimation to simplify spectrum analysis. The Hall sensor is only powered during its readout and switched off otherwise. The sequence control and readout of the Hall sensor is managed by a microcontroller (ESP32C3, Espressif Systems, Shanghai, China). The power supply for all sub-boards is consistently noise-optimized, with digital and analog components having their own galvanically separate battery-powered power supply. The individual potential is only brought to the same potential at a single neutral point (star grounding of all PCBs).

### Pre-polarization electronics

The pre-polarization generator is based on the SiC-nFET G3R160MT17D (GeneSiC Semiconductor, Torrance, USA) with a maximum voltage of ${U}_{{DS}\max }=1.7\mathrm{kV}$ (breakdown voltage is approx. ${U}_{{BD}}\approx 1.3{U}_{{DS}\max }$)^49^ an on-resistance ${R}_{{DSon}}=160{\rm{m}}{\Omega }$ and can handle a non-repetitive avalanche energy of ${E}_{{AS}}=158\mathrm{mJ}$. Three identical SiC-nFETs from the same batch are connected in parallel (cost ~10€ per piece), thereby reducing ohmic losses during the pre-polarization phase and ensuring reliability of the sensor. The SiC-nFETs are operated with the gate driver ACPL-P343 (${I}_{{pk}}=4{\rm{A}}$, ${U}_{G}=0{\rm{V}}/+15{\rm{V}}$, Broadcom Inc., San José, USA).

The pre-polarization duration can be freely adjusted and is limited solely by the ohmic losses of the polarization coil (temperature measurement with a type K sensor). In our measurements, the coil was cooled using active air cooling with compressed air. The semiconductors are passively cooled with heat sinks and exhibit negligible heating. A laboratory power supply with $U\le 20{\rm{V}}$ and $I\le 45{\rm{A}}$ (PE1643, Philips, Amsterdam, Netherlands) is used as a high-current source. In order to trigger the avalanche breakdown, no free-wheeling diodes were used for protection against the voltage peaks due to self-induction of the pre-polarization coil^47,74^. The SiC-nFET was subjected to 10,000 cycles in an additional aging test, and its characteristic curve was examined using a Curve Tracer B1505 (Keysight Technologies) with a high-current and high-voltage extension (further details in SI Chapter 9).

In the developed circuit, the shutdown process can be precisely adjusted to the load inductance via a snubber circuit consisting of a series resistor *R*_*S*_ and a pn-diode (${U}_{{BD}}=2.2\mathrm{kV}$, DNA30E2200PA, IXYS Corporation, Milpitas, USA)^45^. A damping resistor (*R*_*D*_) prevents oscillations by dissipating remaining coil energy after the stopped avalanche (see Fig. 1c). This network enables the adaptation of the circuit for an adiabatic switch-off (${R}_{S}=0\Omega$) and a non-adiabatic switch-off (${R}_{S} > 300\Omega$). For high ${R}_{S}$, the current undershoots during the switch-off. To achieve fastest possible switch-off, ${R}_{S}=600\Omega$ and ${R}_{D}=300\Omega$ were selected. For in-situ characterization, an inductively coupled pickup coil was placed in close proximity to the polarization coil (distance between the centers of the coils $l\approx 27.6\mathrm{mm}$). The pickup coil has two turns of enameled copper wire (${r}_{{Cu}}=0.5\mathrm{mm}$), a coil diameter of ${d}_{{PU}}=38\mathrm{mm}$, negligible DC resistance, and is terminated in series with R=50Ω. An oscilloscope (${BW}=70\mathrm{MHz}$, ${f}_{s}=2\mathrm{GSps}$) without any special galvanic protection measured the voltage of the pickup coil and therefore the current ramp. The coupling inductance to the primary coil has been measured to ${L}_{{mut}}\approx 450\mathrm{nH}$. The largely linear behaving current ramp indicates a constant coil voltage, which can be calculated directly by the relationship ${U}_{{BD}}=L\left|\Delta i/\Delta t\right|$ using the measured slope.

The duration of the avalanche discharge is reproducible within the measurement uncertainty. However, the exact transient waveform is not deterministic because the initiation and, in particular, the termination of the avalanche process depend on stochastic device behavior and the surrounding circuit. Consequently, both the delay between the trigger and the completed switch-off, as well as the exact current ramp, exhibit small shot-to-shot variations. For the optimized measurement sequence at ${B}_{0}=45\mathrm{mT}$, the measured trigger-to-switch-off delay varied between $2\mu {\rm{s}} < {t}_{d} < 26{\rm\mu }{\rm{s}}$. As discussed in SI Chapter 10, Bloch simulations show that even nanosecond-scale variations of the avalanche transient can significantly influence the residual transverse magnetization under these operating conditions.

### Hybrid excitation (classical NMR approach)

For a conventional NMR experiment with RF excitation, the polarization coil at the frontend is replaced by the hybrid *B*_*1*_ polarization coil (specified in Table 1), and the pre-polarization generator is set to adiabatic switch-off mode. The maximum pre-polarization field ${B}_{p}=46\mathrm{mT}$ (at ${B}_{p}=46\mathrm{mT}$) switches off in $t\approx 48\mu {\rm{s}}$. A waveform generator 33500B (Agilent Technologies) with a variable output voltage and pulse length $t=5\mathrm{ms}$ is used as the RF source. A set voltage ${U}_{{RMS}}=28.5{\mathrm{mV}}_{{RMS}}$ corresponds to a flip angle $\alpha =90^\circ$. The waveform generator is triggered at the corresponding timepoint by the built-in sequencer.

For the hybrid excitation experiments shown in Fig. 7, a flip angle of $\alpha \approx 82^\circ$ (${B}_{1}=1.07{\mu\mathrm{T}}$ for ${t}_{Tx}=5\rm{ms}$) was set. The pre-polarization field and duration was set to ${B}_{p}\le 44\mathrm{mT}$ and ${t}_{p}=5984\mathrm{ms}$ respectively. The frequency of the Tx pulse is set to ${f}_{{Tx}}=43.257\mathrm{kHz}$.

### Sequences used

For non-adiabatic shutdown (all *B*_*0*_): $2{\rm{s}}$ Hall sensor measurement, ${t}_{p}=6{\rm{s}}$ (generally, if not stated otherwise), $0.1\mathrm{ms}$ pause, $500\mathrm{ms}$ sampling.

For hybrid excitation at *B*_0_ = 1 mT: $2{\rm{s}}$ Hall sensor measurement, ${t}_{p}=5984\mathrm{ms}$, $10\mathrm{ms}$ pause, $5\mathrm{ms}$ RF excitation, $1\mathrm{ms}$ pause, $500\mathrm{ms}$ sampling.

### Signal filtering and processing

To keep the sensor’s integrated computational power low and thus the hardware inexpensive, complex filtering and fitting of the signal was avoided. Though in this work, the sampled voltage data were processed on a standard computer using MATLAB scripts. Simple algorithms were used to stay compatible with an embedded microcontroller for compatibility. The data was filtered in time domain using a normalized apodization filter by multiplying the measurement data with the following weighting function^75^:

2 $$a(t)=\frac{1}{\mathop{\max }\limits_{\forall t}a(t)}\sin \left(\pi \frac{t}{T}\right)\exp \left(-k\frac{t}{T}\right)$$

with the parameters k=15 and $T=500\mathrm{ms}$ for ${B}_{0}\le 5\mathrm{mT}$, and $T=2\mathrm{ms}$ for ${B}_{0}=45\mathrm{mT}$.

Due to the non-ideal switch-off with varying delay, the first ADC samples during $t\le 7.5\mathrm{ms}$ were not evaluated for ${B}_{0}\le 5\mathrm{mT}$ (all possible interferences have decayed). At ${B}_{0}=45\mathrm{mT}$, this offset is dynamically adjusted in post-processing to follow the actual trigger edge at the gate of the SiC-nFET (also sampled) due to the really short ${T}_{2}^{* }$ relaxation time (details shown in SI Chapter 10). The resulting truncated data, was digitally filtered using a zero-phase bandpass infinite impulse response (IIR) filter (Butterworth, fourth order, bandwidth ${BW}=4\mathrm{kHz}$ for ${B}_{0}\le 5{mT}$ and $BW=500\mathrm{kHz}$ for ${B}_{0}=45\mathrm{mT}$)^76^. The center frequency was calculated based on the magnetic field measured by the Hall sensor. The time-domain signal is then fast Fourier transformed to obtain the frequency spectrum.

For the characterization of the presented hardware and the shown NMR spectra in this work, a semi-automated phase correction was performed, and the signal was cubic interpolated in the frequency domain to $\Delta f=0.1\mathrm{Hz}$. The real part of the spectrum is usually evaluated for all measurements, except for the hybrid excitation measurement and the LFMRI Halbach measurements, due to low *SNR* (and therefore no phase correction was performed). Further first-order phase correction and spectral fitting are described in the SI Chapter 5. For stand-alone sensor operation, these extra processing steps, such as phase correction and interpolation, are unnecessary. Analyzing the absolute spectra is sufficient, as only the peak frequency has to be calculated.

### Ultra-low field magnet and MRI system

The central electromagnet is a solenoid coil with diameter $d=270\mathrm{mm}$, length $l=470\mathrm{mm}$ and containing 470 turns of enameled copper wire (${r}_{{Cu}}=0.5\mathrm{mm}$, more details in SI Chapter 13). To optimize homogeneity, a second layer of 30 turns was added to both ends. The coil efficiency at the isocenter is $k=2.24\mathrm{mT}/{\rm{A}}$ with a calculated homogeneity of $\Delta {B}_{0}=12\mathrm{ppm}$ over a diameter of spherical volume (DSV) of $d=20\mathrm{mm}$ (corresponding $\Delta f=2.5\mathrm{Hz}$ at ${B}_{0}=5\mathrm{mT}$)^50^. The current source BCS3/12 (12 V/3 A, HighFinesse GmbH, Tübingen, Germany) was used for ${B}_{0}=1\mathrm{mT}$ and the current source BCS5/75 (75 V/5 A, HighFinesse GmbH, Tübingen, Germany) was used for ${B}_{0}=3|5\mathrm{mT}$^26^. Previous experiments have shown that these current sources, and thus the resulting ${B}_{0}$, drift slightly, with the more powerful source performing worse. Using another ULFMRI system, the current drift of this source was determined to be $\Delta I\approx -0.47\mathrm{ppm}/{\rm{s}}$. This means that a drift of $\Delta f=42.775\mathrm{mHz}/{\rm{A}}/{\rm{s}}$ should be expected for this magnet. However, this value may differ considerably depending on the source performance. For a typical sensing time of ${T}_{R}=10{\rm{s}}$ in this work, this results in a possible drift of $\Delta f=0.2\mathrm{Hz}$ (1 mT), $\Delta f=0.6\mathrm{Hz}$ (3 mT) and $\Delta f=1\mathrm{Hz}$ (5 mT) per measurement (see SI Chapter 11 for details).

The built-in gradients also serve as first-order shimming. The Z-gradient consists of a 32-turn (${r}_{{Cu}}=0.2\mathrm{mm}$ copper wire) Maxwell coil with a diameter of $d=462\mathrm{mm}$ and a spacing $h=400\mathrm{mm}$, which is identical to the system in ref. ^26^. The simulated magnetic efficiency is ${G}_{Z}=483\mu {\rm{T}}/{\rm{m}}/{\rm{A}}$. The X- and Y-gradients consist of planar coils with 32 turns of ${r}_{{Cu}}=0.4\mathrm{mm}$ wire. They measure $A=612\times 697{\mathrm{mm}}^{2}$ and are mounted $h=595\mathrm{mm}$ apart. The design corresponds to the system in ref. ^26^, but has been scaled up by 20%, resulting in a simulated magnetic efficiency of ${G}_{X}={G}_{Y}=159\mu {\rm{T}}/{\rm{m}}/{\rm{A}}$. The exact gradient accuracy and efficiency are not known. Self-developed, battery-powered current sources with an output of ${I}_{{out}}=\pm \! 250\mathrm{mA}$ were used^26^. The experiment was positioned in a three-layer magnetic shielding chamber (Vacuumschmelze GmbH, Hanau, Germany), with a residual magnetic field $B < 10\mathrm{nT}$ at its center.

### Halbach permanent magnet

The Halbach magnet consists of 536 NdFeB N48 magnets (12 × 12 × 12 mm³, ${B}_{R}=1.395\mathrm{T}\pm 25\mathrm{mT}$) with a bore size of $d=160\mathrm{mm}$ and a length $l=160\mathrm{mm}$. In a previous experiment, the magnet was mapped using a Hall sensor-based mapping, resulting in an inhomogeneity of $\Delta B=8351\mathrm{ppm}$ in a DSV of $d=40\mathrm{mm}$^30^. Scaled to the sensor’s volume, this corresponds to $\Delta B\approx 370\mathrm{ppm}$ ($\Delta f\approx 720\mathrm{Hz}$). The inhomogeneity could be much higher in our setup because of the uncertainty in positioning the sensor.

To vary ${B}_{0}$ and prove the sensor performance, an air-cooled Helmholtz-like coil with diameter $d=72\mathrm{mm}$, distance $h=76\mathrm{mm}$ and n=170 turns of copper wire (${r}_{{Cu}}=0.15\mathrm{mm}$) was integrated into the Halbach magnet (illustrated in Fig. 4e). The isocenters of both magnets were aligned. The frontend was positioned accordingly, so that the sensor sample was positioned at the isocenter of the combined magnet. Compared to an ideal Helmholtz coil, the coil distance was doubled here due to geometrical constraints, resulting in lower magnetic efficiency. FEM simulation shows an efficiency of ${k}_{{BOS}}=2.047\mu {\rm{T}}/\mathrm{mA}$ at the center by adding a significantly high inhomogeneity of $\Delta f=5.349\mathrm{Hz}/\mathrm{mA}$ inside the sensor sample. The overall resistance is R=29.4Ω. The same current source from the ULFMRI was used (BCS3/12, 12 V/3 A). The offset coil was only activated shortly before the NMR measurement (long enough for a steady state of the sample magnetization) and was otherwise switched off to reduce the heating of the Halbach permanent magnets. The entire magnet was positioned in a grounded RF shielding chamber. For active cooling purposes, the polarization and offset coils were exposed to an active airflow using compressed air.

## Supplementary information

Transparent Peer Review file

Supplementary Information

## Data Availability

All data supporting the findings of this study are available in the GitHub repository https://github.com/ppolGTMonster/Hybrid_LowField_NMRProbe. The repository contains representative experimental datasets sufficient to reproduce the data analysis and evaluation presented in this work. Additional experimental datasets are available from the corresponding author upon reasonable request.

## Code availability

All software required to reproduce the data processing and analysis is available without any restriction in the GitHub repository https://github.com/ppolGTMonster/Hybrid_LowField_NMRProbe. This includes the embedded C software for the sensor electronics and MATLAB scripts for data handling, filtering, and analysis. Hardware Design Availability. The hardware design files required to reproduce or extend the sensor are available in the GitHub repository https://github.com/ppolGTMonster/Hybrid_LowField_NMRProbe. The repository includes PCB design files and mechanical CAD files.
