# Supplementary material for: An integrated NMR field probe using rapid non-adiabatic field switch-off for (ultra) low field MRI
Source: Commun Eng. 2026 Oct 28;5:183. doi: 10.1038/s44172-026-00815-0 (PMC13720019; doi:10.1038/s44172-026-00815-0)
Supplement: Supplementary file 1 [file 44172_2026_815_MOESM1_ESM.pdf]

nature portfolio

Peer Review File

An Integrated NMR Field Probe Using Rapid Non-Adiabatic Field Switch-Off for (Ultra) Low Field MRI

Corresponding Author: Mr Pavel Povolni

This file contains all reviewer reports in order by version, followed by all author rebuttals in order by version.

Attachments originally included by the reviewers as part of their assessment can be found at the end of this file.

Version 0:

Reviewer comments:

Reviewer #1

(Remarks to the Author)
This manuscript is set against the background of magnetic field stability issues in ultra-low-field magnetic resonance imaging systems and proposes and implements a sensor scheme for monitoring magnetic field variations. The sensor is based on the well-established physical mechanism in which a rapid non-adiabatic magnetic field switch-off induces transverse magnetization, and it enables measurement of the Larmor frequency under low-field conditions through an engineering implementation. The authors present a prototype of the sensor and validate its performance experimentally under different magnetic field strengths. Based on this work, the main evaluation comments are summarized as follows:

1.The manuscript discusses multiple application directions, including magnet development, shimming, drift monitoring, and gradient evaluation. However, the depth of analysis for each direction is limited. It is recommended that the authors focus more clearly on a single, most compelling core application scenario; otherwise, the work may give the impression of being more technology-demonstration-driven than problem-driven. Although MRI accessibility is used as a motivating background, the probe does not directly demonstrate improvements in image quality or support for specific imaging sequences. The current experiments remain largely at the level of magnetic field measurement and are insufficient to establish irreplaceable value for the MRI imaging workflow itself. It is suggested that the narrative place greater emphasis on magnetic field measurement as the primary contribution, with low-field MRI serving mainly as an application context.

2.The authors repeatedly emphasize that the proposed method operates “without any prior knowledge of the magnetic field.” In practice, however, the system remains significantly constrained by the frequency response of the receive coil, frontend bandwidth, sampling rate, and digital filtering window. In actual operation, a Hall sensor is still required to provide a coarse estimate of the magnetic field range. It is recommended that the authors reconsider this wording and clearly distinguish between “no need for an accurate resonance frequency” and “no need for any prior frequency or magnetic field range information,” as the current formulation may be conceptually misleading.

3.Induction of transverse magnetization by non-adiabatic magnetic field changes is not a new concept and has been extensively discussed in geomagnetic NMR as well as zero- and near-zero-field NMR. The substantive contribution of this work appears to lie more in the engineering realization and the extension of the usable magnetic field range, rather than in the underlying physical mechanism itself. The current manuscript conflates known physical phenomena with new engineering implementations in its novelty argument. It is recommended that the authors de-emphasize claims of a new physical mechanism and instead focus on engineering feasibility and system-level advances.

4.Due to the relatively long pre-polarization time, the repetition time (TR) and temporal resolution of the method are fundamentally limited, which prevents its application to dynamic magnetic field variation monitoring. Although this limitation is mentioned in the discussion, no quantitative analysis is provided. The authors are encouraged to specify the minimum achievable TR and maximum update rate and to compare these values with existing field-monitoring approaches so that readers can better assess the practical value of the method in dynamic scenarios.

5.The system is described as “easy to replicate” and “suitable for low-resource environments,” yet its core operation relies on

kilovolt-level transient high voltage, avalanche-mode SiC devices, and precise control of power-electronics behavior. Even if the component cost is relatively low, such a design still requires specialized power-electronics expertise and strict safety measures. A more cautious discussion of replication difficulty and safety barriers is recommended to avoid over-stating the accessibility of the approach.

6.Although avalanche endurance tests are reported for the SiC devices, the manuscript does not provide a systematic evaluation of frequency reproducibility and drift of the complete probe system over repeated measurements, extended operation times, or varying environmental conditions. For a system intended for magnetic field characterization and stability monitoring, this omission is particularly significant.

7.The proposed method is mainly compared with Hall sensors, which are not the only or necessarily the most relevant competing solutions in low-field MRI. The lack of quantitative comparison or discussion with other low-field NMR probes or RF-based excitation approaches weakens the conclusion regarding performance advantages.

Reviewer #2

(Remarks to the Author)
see attached document.

Reviewer #3

(Remarks to the Author)
I co-reviewed this manuscript with one of the reviewers who provided the listed reports. This is part of the Communications Engineering initiative to facilitate training in peer review and to provide appropriate recognition for Early Career Researchers who co-review manuscripts.

Version 1:

Reviewer comments:

Reviewer #1

(Remarks to the Author)
Thank you for the detailed and constructive responses. I appreciate that the authors have substantially revised the introduction, added comparisons with other magnetic field measurement approaches, and clarified several technical limitations. These revisions improve the manuscript.

From a reviewer's perspective, I would still encourage the authors to make the central contribution more explicit and focused. The strongest aspect of the work appears to be the engineering realization of an integrated NMR-based magnetic field sensor for low-field MRI-relevant conditions, rather than a broad demonstration across multiple MRI-related applications. A clearer emphasis on magnetic field measurement, system integration, and practical sensing performance would make the manuscript more convincing.

Reviewer #2

(Remarks to the Author)
see attached pdf

Reviewer #3

(Remarks to the Author)
I co-reviewed this manuscript with one of the reviewers who provided the listed reports. This is part of the Communications Engineering initiative to facilitate training in peer review and to provide appropriate recognition for Early Career Researchers who co-review manuscripts.

Open Access This Peer Review File is licensed under a Creative Commons Attribution 4.0 International License, which permits use, sharing, adaptation, distribution and reproduction in any medium or format, as long as you give appropriate credit to the original author(s) and the source, provide a link to the Creative Commons license, and indicate if changes were made.
In cases where reviewers are anonymous, credit should be given to 'Anonymous Referee' and the source.
The images or other third party material in this Peer Review File are included in the article's Creative Commons license, unless indicated otherwise in a credit line to the material. If material is not included in the article's Creative Commons license and your intended use is not permitted by statutory regulation or exceeds the permitted use, you will need to obtain permission directly from the copyright holder.
To view a copy of this license, visit https://creativecommons.org/licenses/by/4.0/

Reviewer 1

We would like to express our sincere gratitude for the insightful comments and the examples you provided. Your feedback has been incredibly helpful, and we truly appreciate the time and effort you put into reviewing our work.

Please see below, for a point-by-point response to your comments and concerns. All changes are commented in the revised manuscript with tracked changes.

Major Comments

1. The manuscript discusses multiple application directions, including magnet development, shimming, drift monitoring, and gradient evaluation. However, the depth of analysis for each direction is limited. It is recommended that the authors focus more clearly on a single, most compelling core application scenario; otherwise, the work may give the impression of being more technology-demonstration-driven than problem-driven. Although MRI accessibility is used as a motivating background, the probe does not directly demonstrate improvements in image quality or support for specific imaging sequences. The current experiments remain largely at the level of magnetic field measurement and are insufficient to establish irreplaceable value for the MRI imaging workflow itself. It is suggested that the narrative place greater emphasis on magnetic field measurement as the primary contribution, with low-field MRI serving mainly as an application context.

Thank you for this valuable comment. As also noted in our response to Reviewer 2 (Major Point 1), we would like to clarify the context of the original introduction.

The manuscript was initially prepared for a Nature Communications special issue on "Health in Africa," linked to the United Nations Sustainable Development Goals. Given that the sensor was developed for our low-field MRI systems targeting accessible diagnostics in underserved regions, this long introduction was intentional. Following editorial advice, the manuscript was subsequently transferred to Nature Communications Engineering without further modifications.

We recognize that the scope of this journal is more engineering-focused. Nevertheless, the broader motivation remains important to us, particularly in the context of global health challenges and equitable scientific visibility.

We have significantly shortened and focused the introduction while trying to retain all essential references.

Given the stringent requirements of MRI on magnetic field stability and homogeneity, it serves as an ideal benchmark for validating our sensor performance. Beyond this, the sensor is applicable to a broader range of use cases.

What we have changed:

1. We rewrote most of the introduction, including the explanation of the SDGs and the use of low-field MRI.
2. We added a paragraph to the introduction referencing other technical solutions for magnetic field measurement.
3. In the Outlook section, we referenced additional applications for our sensor system.

2. The authors repeatedly emphasize that the proposed method operates “without any prior knowledge of the magnetic field.” In practice, however, the system remains significantly constrained by the frequency response of the receive coil, frontend bandwidth, sampling rate, and digital filtering window. In actual operation, a Hall sensor is still required to provide a coarse estimate of the magnetic field range. It is recommended that the authors reconsider this wording and clearly distinguish between “no need for an accurate resonance frequency” and “no need for any prior frequency or magnetic field range information,” as the current formulation may be conceptually misleading.

Thank you for this perspective. Below, we provide some additional explanations:

Rx Coil: It is true that the coil must be roughly tuned to the subsequent LNA to minimize the LNA’s noise figure through proper impedance matching. Based on the measurements in Fig. 5, in which the magnetic field was swept but the coil settings were not changed, B_0 can deviate by an average of $\pm 24\%$ from the matching frequency of the Rx-coil. This operational range is significantly greater than the error of the Hall sensors and was definitely sufficient in our measurements. In a future development of the front end, the coil may be adjusted roughly to the magnetic field measured by the Hall sensor using switchable capacitors (or capacitor ICs) since a great precision is not necessary.

Hall Sensor: Our system is referred to as an "integrated sensor," through which we aim to combine the advantages of combined magnetic measurement methods. Hall sensors enable excellent, fast, and robust magnetic field measurements and can be purchased as integrated chips for very little money (~1€ in our case). So, the data from the Hall sensor is utilized only in retrospective processing to simplify the digital NMR spectrum analysis, which improves the robustness of the spectral analysis and fitting in the very low SNR regimes. Otherwise, the NMR signal and subsequently the field can be analyzed and fitted accurately with the NMR sensor data alone.

To the best of our knowledge, this is the typical concept of an integrated NMR based sensor front-end. In this work, only the Rx coil matching was changed for all measurements. Nothing else in the hardware was changed (e.g., anti-aliasing filters, DC-block capacitors,…), except for a rough adjustment of the overall gain to prevent railing in different environments. Thus, the same hardware setup can measure the entire range between 1 mT and 50 mT, and the bandwidth is sufficient for this purpose.

A priori knowledge: the new Figure 9 with shown Hall sensor tolerances shows how the integrated Hall sensor significantly simplifies spectrum analysis. Previous characterizations of the magnet provided only a rough idea of the magnet's magnetic field (especially at the sensors location), so the sensor was only roughly adjusted to an approximate frequency. However, it was possible to determine the spectrum using the provided Hall sensor data without any further interactions. This means that no detailed knowledge is necessary to transmit a resonant Tx pulse, for example.

However, we have revised the manuscript in several places and addressed your comments.

3. Induction of transverse magnetization by non-adiabatic magnetic field changes is not a new concept and has been extensively discussed in geomagnetic NMR as well as zero- and near-zero-field NMR. The substantive contribution of this work appears to lie more in the engineering realization and the extension of the usable magnetic field range, rather than in the underlying physical mechanism itself. The current manuscript conflates known physical phenomena with new engineering implementations in its novelty argument. It is recommended that the authors de-emphasize claims of a new physical mechanism and instead focus on engineering feasibility and system-level advances.

We were inspired to use this known excitation method by earlier experiments in our separate ULF system ($B_0 \ll 1mT$). To address the usability, we referred to previous systems in the fields of geological NMR and ultra-low-field NMR in the introduction and discussion.

However, to our knowledge, previous systems are limited to extremely low fields, which play hardly any role in conventional low-field MRI. Due to our new ability to generate very high current ramps compactly (thereby accelerating the collapse of strong pre-polarization fields), this physical effect extends to magnetic fields relevant for low-field MRI.

Although this physical effect is directly derivable from the Bloch equations, we have found that it is almost unknown in the low-field MRI community. Partially because it is difficult to achieve a non-adiabatic condition. For this reason, we briefly explained this concept in the "Results/Non-Adiabatic Switch Off the Pre-Polarization Field" section and used to empathic language to draw the community's attention to this interesting possibility.

To improve understanding, we rewrote the manuscript in the introduction and discussion sections. Additionally, we added a new chapter 3 to the Supplementary Information that focuses more on this topic.

4. Due to the relatively long pre-polarization time, the repetition time (TR) and temporal resolution of the method are fundamentally limited, which prevents its application to dynamic magnetic field variation monitoring. Although this limitation is mentioned in the discussion, no quantitative analysis is provided. The authors are encouraged to specify the minimum achievable TR and maximum update rate and to compare these values with existing field-monitoring approaches so that readers can better assess the practical value of the method in dynamic scenarios.

This is a very important point that determines, or rather limits, the possible application of the developed sensor. Due to word count limitations, we were not been able to address it in detail.

Our intended purpose of the Results chapter and Fig. 6 was to address this issue. Depending on the desired measurement concept and the realized frontend setup, the repetition rate can vary significantly. For example, a possible readout time with the presented sensor is, if the fastest possible measurement is desired and the Hall sensor readout can be omitted:

- pre-polarization of $t_p \geq 1s$ can be chosen (in the case of water, depending on B_0)
- reduce sampling to $T_S \leq 200ms$ due to short T_2^*.
- this would result in an update rate of approx. 1.5 seconds.

This is an important consideration, particularly in comparison to conventional NMR samples based on resonant excitation (see Reviewer 2, Major Point 2).

We have revised the manuscript, including a comparison with conventional NMR samples in the Discussion section.

5. The system is described as “easy to replicate” and “suitable for low-resource environments,” yet its core operation relies on kilovolt-level transient high voltage, avalanche-mode SiC devices, and precise control of power-electronics behavior. Even if the component cost is relatively low, such a design still requires specialized power-electronics expertise and strict safety measures. A more cautious discussion of replication difficulty and safety barriers is recommended to avoid over-stating the accessibility of the approach.

We understand that our system may appear complex, and replicating it may seem challenging.

However, our primary goal throughout the development process was to keep the system as simple as possible. Therefore, we are still convinced, that is valid to see the developed system as easy-to-replicate.

During the discussion of the manuscript, we analyzed the safety aspect. In principle, any voltage source exceeding 2 kV can be used for the sensor. However, there may be a risk of electric shock if the used source is connected to ground potential, especially if a significant current can flow. This can be dangerous, so the described safety measures must be taken in such cases.

In our case, however, the energy is low ($W < 25mJ$), and the voltage source (the SiC transistor in avalanche-mode) cannot supply any relevant current. Additionally, there is no connection to ground potential if the pre-polarization generator low voltage power supply ($U < 20V, I < 25A$) is galvanically isolated using a transformer, as in our case.

Therefore, the worst-case scenario upon direct contact with both poles is an electric shock equivalent to an electrostatic discharge from the human body (e.g., in winter with dry air). Thus, simple contact protection (e.g., a few millimeters of air) is sufficient. Using the galvanically isolated pickup coil from Fig. 1f to adjust the circuit settings also eliminates the need to connect complex measurement technology on the board, which poses potential risks to the user. Another hazard stems from the transistors, which may be destroyed after many breakdowns. Our tests, which show an expected lifespan of way over 10,000 cycles, show that no danger is to be expected here either. A rising reverse current and a rising R_{DSon} would indicate that the transistor has reached the end of its life before damage occurs.

We recognize that a basic understanding of electrical engineering is necessary for replication. Nevertheless, in our opinion that this is not sufficient reason to restrict the ease of replication, particularly in light of the sharing of all design files.

We have rewritten the relevant section of the discussion to make it clearer.

6. Although avalanche endurance tests are reported for the SiC devices, the manuscript does not provide a systematic evaluation of frequency reproducibility and drift of the complete probe system over repeated measurements, extended operation times, or varying environmental conditions. For a system intended for magnetic field characterization and stability monitoring, this omission is particularly significant.

That's right. That is indeed an important parameter for sensor systems. Due to word count limitations, we did not address this point in the manuscript until now.

We added a new paragraph to the Discussion section describing the potential drift and stability of the sensor.

7. The proposed method is mainly compared with Hall sensors, which are not the only or necessarily the most relevant competing solutions in low-field MRI. The lack of quantitative comparison or discussion with other low-field NMR probes or RF-based excitation approaches weakens the conclusion regarding performance advantages.

Thank you for this suggestion. Because to word limits, this aspect could not be included in the original manuscript. See also our answer to the Major point 4 of Review 2.

In the revised version, we have incorporated a comparison in the introduction between NMR excitation and other common magnetic field sensing approaches. Additionally, the discussion now includes a comparison with conventional NMR field probes, addressing aspects such as technology, applicability, sensing volume, and update rate.

Reviewer 2

We appreciate your comments, examples, and feedback. Your input has been very helpful. Please see below for a point-by-point response to your comments. All changes are commented in the revised manuscript with tracked changes.

Major Comments

1. a) Writing and Structure: The Introduction is overly long and written and the content is not totally appropriate for a technical instrumentation paper. Extended discussion of global development themes and SDGs, while well intentioned, distracts from the core engineering contribution. This section should be condensed and refocused on the technical gap in low-field NMR field sensing and how this work addresses it.

Thank you very much for this comment. As with our response to Reviewer 1's Major point 1, we would like to briefly explain why we wrote this introduction in the original manuscript.

Explanation:

Originally this manuscript was submitted to Nature Communications for the special issue on “Health in Africa,” which addressed the United Nations Sustainable Development Goals specifically. Since we developed the sensor for our self-developed low-field MRI systems (aiming to enable high-performance diagnostic imaging in remote areas) this call seemed as a perfect fit for the developed sensor. However, the editor suggested that we submit the manuscript to Nature Communications Engineering unchanged, as it might be a better fit there. We then did that.

We understand that the focus of this journal is different and significantly engineering related. At the same time, addressing this inequality is a strong motivation for us as scientists, and we want to provide a platform for this really relevant topic as well. This aligns with the Nature portfolio’s goal of raising awareness of the SDGs, promoting their implementation, and advocating for "citation justice" to highlight excellent research by scientists from currently underrepresented regions.

We have significantly shortened and focused the introduction while trying to retain all essential references.

Since MRI places extremely high demands on the stability and homogeneity of the magnetic field in general, this application is an excellent benchmark for verifying our magnetic field sensors. Of course, there are many other applications, where this sensor may be utilized.

What we have changed:

4. We rewrote most of the introduction, including the explanation of the SDGs and the use of low-field MRI.
5. We added a paragraph to the introduction referencing other technical solutions for magnetic field measurement.
6. In the Outlook section, we referenced additional applications for our sensor system.

b) The manuscript would benefit from editing to make concepts more clear and concise. There are also numerous single-sentence paragraphs that disrupt the narrative flow.

Thank you very much. We were constrained by the word limit, so we rewrote sentences without losing any information.

We revised the manuscript and rewrote many unpolished sentences.

2. Receive coil description: the receive coil design and geometry and tuning/matching procedure are not clearly explained, despite being central to probe sensitivity and bandwidth. Greater detail (descriptive and perhaps a figure) would be necessary for reproducibility.

We added a new section to the methods chapter 4 "Methods/II: Simulation and Mechanical Design of the Receive Coil" and provided a more detailed description of the rx coil manufacturing.

Additionally, the complete design files and circuit boards are available in our github. However, we are planning to extend and provide more detail in the repo upon publication.

We hope the additional paragraph clarifies the coil fabrication process. Unfortunately, due to figure limitations, we are unable to include an additional figure.

3. Data Quality and Convincingness of Conclusions: A major concern is the quality and cleanliness of the experimental data. Several datasets appear noisy or low in SNR, which weakens the strength of the conclusions. In some cases, noise is attributed to laboratory interference, yet the manuscript also states that RF shielding at these frequencies is relatively easy to implement. Many results rely on single acquisitions without averaging or uncertainty analysis. The authors argue that averaging is difficult due to magnetic field drift. While drift is clearly present, averaging nonetheless seems necessary to convincingly demonstrate sensitivity and repeatability. If thermal effects from the prepolarization or offset coils are driving drift, this could potentially be mitigated (or at least discussed) for example, by operating at a thermal steady state, increasing duty cycle stability, or implementing drift correction.

We thank the reviewer for their detailed feedback. We believe the relevant aspects were described in the original manuscript, but we acknowledge that further clarification is needed.

First, we will briefly address the points you raised, and then we will summarize the made revisions:

1. datasets appear noisy or low in SNR

We assume this primarily refers to the shown spectral data in Figures 5a, 8a, and 9a since the measurements in Figure 1 are extremely robust and reproducible.

As predicted by the simulations in Fig. 2d, the spectra shown in Fig. 5a, 8a, and 9a demonstrate the limit of the presented sensor. At low fields of 1 mT (in Figures 5a and 8a), the induction in the Rx coil is small due to the low Larmor frequency (since $u{\sim}B_0$). The Rx front-end's noise performance is significantly reduced due to worse matching, leading to a large drop in SNR.

As predicted by the simulation and the adiabatic condition in equation (2.1), at $B_0 = 45mT$, the proposed excitation method operates at its absolute limit, leaving only a small transverse magnetization. Here, we demonstrate that a measurement is possible even when the adiabatic condition appears to be satisfied. The sharp increase in noise level (approximately a factor of 10 compared to 1 mT) and the short FID due to magnetic field inhomogeneity result in a low SNR in the spectrum.

Changing the magnetic field to the optimum of the designed sensor increases the achievable SNR in the presented measurements by approximately +20dB.

Our goal of this sensor concept is stand-alone operation, which is why the frontend was developed to be as compact as possible. Therefore, complex digital filters in post-processing were intentionally omitted (so the postprocessing done on the computer could be translated to an embedded microcontroller, or FPGA).

Nevertheless, all recorded spectra can be robustly and easily fitted. These less error prone fitted data could be used for further data processing steps. However, these curves were not shown in the original manuscript. We have revised the manuscript at this point.

2. noise is attributed to laboratory interference

It is correct, that due to the highly sensitive receiver design (including an Rx coil with many windings and an Rx path with very high gain), even small environmental disturbances or interference can cause the electronics to rail.

However, we consider stochastic white (thermal) noise, such as Johnson-Nyquist noise from the Rx coil and the additional noise added by the Rx-path, to be the dominant source of noise. This was covered in Chapter 4 of the Supplementary Information in the original manuscript and is illustrated in Fig. 2d, which shows that the resistance of the Rx coil rises significantly with rising frequency (due to the skin-effect).

Additional noise sources, such as quantization noise from the ADC at reduced signal gain (see Fig. 5a) and thermal sample noise, also degrade the theoretically possible SNR. Since SNR was considered in the bandpass filtered spectrum laboratory interferences (causing single frequency peaks) should not degrade the SNR to much (if railing and gain reduction is avoided).

3. RF shielding

In our measurements we have two distinct settings:

The ULF measurements were performed in an electromagnetic shielded chamber. Shielding against the Earth's magnetic field (and other residual magnetic fields) was particularly important to us so that we could set the exact wanted B_0 at the sensor center, since the efficiency of the electromagnet was known and highly precise current sources were used. This allowed us to characterize the precision of the NMR sensor we developed as well as its operating range at single setting. The goal was not to minimize noise caused by interfering RF signals.

The LF measurements were performed in an small, RF shielded chamber in our electrical lab (see Fig. 4d). Due to the small signal, the goal here was indeed to minimize random coupling and interferences at the desired Larmor frequency. For instance, we discovered that the switching power supplies of equipment in the lab (in this case, the power supply of a differential probe) emit interference close to the expected Larmor frequency, which superimposed on our signal. The simple RF chamber effectively suppressed this interference in the MHz range.

We did not install additional RF shielding on the sensor head itself to prevent transient eddy currents induced by the strong current ramps of the pre-polarization coil. However, a mesh-like shield could certainly be installed directly on the sensor head in a future development.

4. single acquisitions / averaging / uncertainty analysis

Here, we want distinguish between the two measurement settings again to answer this raised question:

1) ULF Range: For the spectra shown in Fig. 5, three sequential measurements were averaged in the frequency domain. For the spectra shown in Fig. 8, at least four sequential measurements were averaged in the frequency domain (or 100 averages in the first tile of the plot).

From previous setups, we know that the current sources used are very precise and have an extremely accurate analog control of the output current (since originally developed for LASER experiments).

However, due to thermal heating, the resistance of the B_0 coil changes, forcing the source to slowly readjust its output. Additionally, inductive couplings between the pre-polarization coil and the B_0 coil can be considered a disturbance of the control. The B_0 drift caused by the current source was analyzed in an additional experiment (see Supplementary Information 10). Therefore, especially in long measurement series, it must be assumed that the magnetic field drifts slowly and corrupts the measurement series. When examining the gradients in Fig. 7, the frequency drift (a few Hz difference per step) was determined for gradients applied at different currents. Therefore, no averaging was performed here to fasten the measurement sequence.

2) LF Range: During the measurement series in Fig. 9, the actual goal was to average the measurements to increase the intrinsically low SNR. However, during the analysis, we realized that this was not possible due to the magnetic drift. Therefore, averaging is not possible here.

In the future, it would certainly be interesting to characterize the sensor in a stable and homogeneous magnetic field based on a (superconducting) electromagnet at this field strength and determine the maximum achievable SNR of the excitation method.

5. drift correction

The ULF system would require an additional (commercial) NMR sensor system whose output is used as a reference signal for the current source control. However, in our case, the used current sources do not offer an additional sensing input, since they operate autonomously.

For the LF system, we measured an unshielded permanent magnet with high inhomogeneity as a proof of concept for this sensor type. Due to the very short T_2^* and the unknown delay of the avalanche process of the pre-polarization generator, the signal is already very small in principle (see SI Chapter 9). Significant improvements are expected once the magnetic field is shimmed (potentially using electric coils). We consider the shown measurement to be proof of principle and a demonstration to the quality of the measurement principle itself. Even in a highly inhomogeneous, drifting magnetic field, where only a small amount of transverse magnetization remains after excitation, the presented system works and provides detailed insights into the magnetic field.

Changes we made to the revised manuscript:

1. In the section "Results/v. NMR Measurements in Ultra-Low Field for B0 <5 mT," we described our data workflow and processing and the potential reasons for higher noise at $B_0 = 1mT$.
2. We added additional fitted curves to the spectra in Fig. 5. For the SNR curves, we fitted the measured points and plotted them with an uncertainty margin for greater clarity. The figure caption has been revised accordingly.
3. We revised the caption in Fig. 6 and described the error/uncertainty.
4. In Fig. 7, we modified the figure, adjusted the caption, and included the uncertainty of the fit.
5. In Figure 8, we included the fitted spectra and described them in the caption.
6. We completely revised Fig. 9 (along with the specific comments 24, 25, and 28).
7. The Methods section, "VIII. Signal Filtering and Processing," references a new chapter 5 in the Supplementary Information.

8. Chapter 5 of the Supplementary Information has been added and now describes our complete data flow, from sampling to the final spectrum fit. Exemplary measurement data are shown for each step.

4. Comparison to Standard Methods: Finally, the comparative advantage over conventional NMR probes should be more critically examined. While the “no prior field knowledge” excitation is valuable, the receive coil still requires tuning/matching. In many systems, the same coil infrastructure could support broadband or frequency-swept transmit excitation, which partially offsets the claimed operational advantage. A more balanced comparison to standard probes would strengthen the manuscript’s positioning. Overall, I believe the data should be made cleaner through improved acquisition strategy, averaging, and/or post-processing. Although the method is scientifically very interesting, it is not convincing that there is a clear advantage over basic NMR field-probe based on the data quality presented.

Thank you very much for this suggestion (see also our answer to the point 7 of Review 1, Major).

In the revised manuscript, we addressed this comparison in the introduction by comparing NMR excitation with other typical magnetic field measurement methods. In the discussion section, we added a paragraph comparing our system with other conventional NMR field probes in terms of technology, applicability, sensing volume, update rate, etc.

Specific Comments

Introduction:

1. Sections discussing SDGs (lines ~43–54) are not well worded and extend beyond what is necessary to motivate the work. Consider condensing (if kept).

The relevant sections have been shortened considerably, while retaining most of the key citations.

2. The hydrocephalus example (lines ~60–62) needs clarification and supporting citation (if kept)

The relevant sections of the introduction have been shortened, and this specific example is not mentioned anymore. However, interested readers can find further details in the references (e.g., Ref. 1 and Ref. 12).

3. Discussion of “brain drain” and local job markets (lines ~74–85) feels out of scope for a hardware instrumentation paper.

This particular section was included in the initial submission of this manuscript because it was a point of focus for the targeted issue. This was addressed in this subsequent revision now and adapted to the focus of the journal (further information in answer to Review 1 Major point 1 and Review 2 Major point 1).

4. When discussing signal scaling (e.g., line ~109), clearer quantitative formatting (percentages or scientific notation) is recommended.

We have changed the numbers in the text from “1/3600” to 280 ppm and “1/210,000” to 5 ppm

5. (111-113) Expand comparison to existing field probes/gaussmeters (e.g. Metrolabs, Lakeshore, etc): What dynamic ranges do they cover? Why are they insufficient during magnet construction?

Additional details and references have been added (see also answer to Reviewer 1, Major, Point 7, and Reviewer 2, Major, Point 5).

Results:

6. Equation (2.1) is key but difficult to interpret and not well explained in the text.

This formula is examined in detail in a new Chapter 3 of the Supplementary Information. The new Figure S2 included there now investigates the adiabatic condition in the time domain and for various settings of B_0 and B_p.

Please note that due to these new chapters in the Supplementary Information, all SI references in the manuscript have been updated

7. Clarify why the adiabatic condition is easier to violate at lower fields.

This analysis is presented in the new Chapter 3 of the Supplementary Information.

The left-hand side of Equation 2.1 (Larmor frequency) becomes slower as B_0 decreases, so that, with the right-hand side remaining almost constant (rotation of the effective magnetic field $B = B_0 + B_p$ around B_0), the adiabatic condition is more easily violated as B_0 decreases.

8. Clarify notation: tp is used for both switch-off time (ns) and polarization duration (s).

Thank you for your feedback! We have updated the manuscript so that the switch-off time (ns) is now referred to as t_R (for the duration of the current ramp). The total pre-polarization (s) duration remains t_p.

9. Fig. 1b appears truncated (precession circle cut off).

The "broken" circle representing the rotation of $M_{\perp}$ was intentionally shown only partially to keep Fig1 compact. We have adjusted Fig. 1b to improve clarity.

10. Fig. 1e is difficult to interpret, but reverse-current across avalanche cycles appear quite variable.

The reverse current was shown in pA in the figure, so the tolerance of the measuring instrument used was already the dominant factor resulting in the variable differences. We have adjusted the scale in Fig. 1e. Our primary goal was to demonstrate that there is no change in the reverse current even after 10,000 avalanche cycles. Further details regarding the measurement can be found in the Supplementary Information Chapter 8.

11. The intentional avalanche operation of SiC-nFETs is a strong novelty. This should be emphasized quantitatively (ramp rates vs prior methods).

Thank you for your feedback. We are pleased that this new method is being so well received. Due to word count limitations, further details could not be included here. Some more information and comparisons are given in the discussion (paragraph 2), where our method is compared with existing methods in geological NMR.

12. Fig. 2a suggests a spherical winding encasing the sample, whereas earlier figure implies perpendicular solenoid – please clarify this.

The Rx coil features a spherical winding, as illustrated in the cross-sectional CAD rendering shown in Figure 1a. Figure 2a has been updated to reflect these changes.

13. Provide contour scales and inhomogeneity metrics in Fig. 2b.

In the previous plot, the contours were drawn in white, but were barely visible after exporting the document. In Fig. 2b, we have now changed the contour lines to black.

14. Fig. 2e plot and caption are generally confusing. For example, why do the simulations have error bars?

We have included additional information in the figure caption and referred the reader to the relevant calculation in Chapter 7 of the Supplementary Information now.

This plot compares the measured and simulated signal amplitudes as a function of B_p. This fit is performed separately for the three measured magnetic fields. From these fits the calculated slope is shown. The whiskers in the graph correspond to the error of the linear fit. Due to the attempt to represent physical processes (such as T_2) as precisely as possible in the simulation, regression errors also arise when fitting the simulated signal amplitudes.

When considering the SNR, these errors are negligible in the simulation, most probably since the total noise is not perfectly represented.

However, a comparison of the simulation and the measurement, as well as the overlapping error regions, demonstrates that the simulation is, in principle, consistent with the measurement and can therefore be used for the system design.

This topic is described bit more detailed in Chapter 2ii now.

15. (349-351) "The implemented design offers the possibility of being easily adjusted for high B0 resolution (strong Bp, long tp, long TR), for high temporal resolution (strong Bp, short tp, short TR), or for energy efficient long-term drift measurements (weak Bp, long tp, long TR)". This is an interesting statement – please elaborate, particularly on the "high resolution" part – what resolution are you referring to and why does long tp and TR help – is this just related to the T1?

We have rewritten this section to clarify the proposed settings.

In general, it is important for the applications described here to determine the Larmor frequency as accurately as possible, which requires a small FWHM (see Supplementary Information, Chapter 11). This is achieved by maximizing the spectral signal amplitudes. For various applications, we have explained here how the pre-polarization phase can be adjusted to achieve the highest possible signal amplitudes under the given constraints.

16. The simulated crossover (~26 mT) where RF excitation surpasses non-adiabatic SNR is an important result—highlight more explicitly.

We have adjusted the text and clarified the result and highlighted it in the discussion.

17. Figure 6: Linear and exponential fits are not convincing. Only single spectra were acquired, can averaging be done? Also, error bars are recommended.

For this measurement, no averaging was performed in order to avoid introducing additional drift from the main magnetic field to our measured results (for details, see Chapter 10 of the Supplementary Information). We have added some information in the figure captions explaining what the error bars represent (95th percentile).

18. Figure 7: This figure is confusing. Please clarify the goal and procedure of the gradient evaluation. Are you trying to show gradient strength or linearity, frequency drift, or estimate position of the probe? At first glance, one would assume that the x-axis is showing the probe position, but it seems that the gradient strength is being varied? If so, it would be more intuitive to show results as a function of current instead of G. Why are the results so different for different B0s? The data is also not clean and the fit it is not good. Error bars should be added.

In this particular measurement process, the sensor was installed in close proximity to the isocenter of the main magnet. However, while the strength of the gradient systems was known prior to these experiments (from simulations), the positional offset between them is unknown.

In the shown measurement, the sensor was positioned in the center once and the gradient current was swept, causing the measured Larmor frequency to vary slightly. This variation was fitted using the applied gradient strength (resp. the set current). With the linear fit, the absolute position of the front end relative to the isocenter of the respective gradient can be determined. The calculation is provided in Chapter 7 of the Supplementary Information. Specifically, this measurement can be used, for example, to determine the mechanical positioning accuracy of freshly installed gradient systems.

We have modified the figure accordingly and added further details to the figure caption and the main text.

19. Section vi: "Testing at this low-field strength is considered a critical challenge to the sensor concept, since matching of the Rx and Tx coils is difficult" ← isn't the matching of the Rx coil equally challenging the NA method?

Yes, this is the same issue with both methods.

Achieving an optimal match at low frequencies can pose a great challenge. This is primarily due to the lack of available large adjustable capacitors. As a result, fixed capacitors with inherent tolerances must be iteratively resoldered. In addition, the measurement process on the VNA itself is time-consuming due to needed set narrow bandwidth and the number of required data points. Consequently, the signal boost due to resonance of the Rx coil (for example, with resonant excitation) is poor, or the noise matching with the subsequent amplifier stages is poor (resulting in an increased noise figure in the presented system).

For this reason, we included this measurement in the manuscript, as it can be considered a critical test for a sensor (if the working principle is shown at very low field strengths, it is evident that it will also work at higher field strengths).

20. Section vi: Why is the term "thermal polarization" used? Isn't the polarization physics the same for the "normal" B0 polarization and the prepolarization?

Nice catch! That is indeed a naming error. We've corrected it.

21. Figure 8: Averaging and/or denoising is needed here. The fidelity of these measurements is not good. There is not a compelling trend in b and c.

As mentioned in point 19, we consider the measurement at $B_0 = 1mT$ to be a critical limit case and wanted to demonstrate that a valid measurement can be performed even at these low field strengths and small amplitudes. Due to the small sample size and the low field strength, the spin polarization is small, and consequently, so is the signal.

In this measurement, at least 4 measurements were averaged (during the B_p sweep), and in the first measurement, 100 measurements were averaged to make a signal visible at all.

Despite the low SNR, the spectrum can be fitted without difficulty and further analyzed based on the measured data or on the fitted spectrum (see Supplementary Information, Chapter 5). We have included these additional fits in the figure and wrote about it in the figure caption.

22. Section vii: "resulting in an averaged frequency drift of Δf=835Hz". A drift of 835Hz every 29s seems extreme. Is there significant heating from the polarization coil that is causing this?

In fact, the measured drift is very high. The temperature increase caused by significant ohmic power loss leads to substantial drift (a consequence of the magnet's small size). In addition, the magnet is not shimmed, resulting in a short T2* time and thus limited measurement resolution. Consequently, the performance of the presented MRI scanner is below that of typical brain-imaging MRI systems and should be regarded as an upper-bound condition. Therefore, we shortened the sentence and simplified Figure 9.

Additionally, we have now included the well-known temperature-dependent variation of the magnetic field in the discussion and added citations to References 59 and 60.

23. Section vii: "Due to the very short *T2**, the resolution of the *B0* mapping is limited in this unshimmed case". Specify what is meant by "resolution" here.

We were referring to the accuracy of the frequency measurement (see details in Chapter 11 of the Supplementary Information). We have revised this sentence to clarify this point.

24. Section vii: There is a comparison to Hall sensors here. I assumed different sensors have different dynamic ranges and resolutions. For example you could compare to some commercial off-the-shelf options (e.g. from Metrolabs).

This is not intended to be a comparison of our system to Hall sensors. As shown in Figure 3, the Hall sensor is integrated into our sensor frontend. Its measurement is necessary to simplify the spectral analysis (see the Supplementary Information, Chapter 5). This Hall sensor result is shown in Fig 9a.

To address this concern, we have updated Figure 9a and its caption to provide further clarification. In the introduction, we have outlined additional measurement methods.

We have therefore updated Figure 9a and its caption to clarify this point. Additionally, we have now described additional measurement methods in the introduction.

25. Figure 9a: This data is not robust and it is displayed in a way that is hard to read/intepret. It seems odd that the x-axis is in kHz instead of MHz. There is no way to tell see the drift in these plots because the x-axes are very compressed. Perhaps overlaying the measurements in 3 plots would be more compelling? In general, I think higher fidelity measurements could be made a basic NMR experiment using a broadband Tx coil and frequency swepted pulses.

We have reorganized and edited the figure for better clarity. In addition, the fitted spectra are shown.

Since our measurement principle requires violating the adiabatic condition (see Supplementary Information, Chapter 3), the measurement at $B_0 = 50mT$ is very difficult, as only very small transverse magnetizations remain for the measurement. Consequently, the achievable SNR is also rather low for this initial proof-of-principle setup. With a front end optimized for this range (essentially a smaller sample and a smaller B_p coil for a significantly stronger B_p at the same current), the achieved SNR would most probably better.

26. Figure 9b: unclear what you are trying to show here.

We wanted to show the frequency drift observed in the individual measured repetitions in Fig 9a. However, in accordance with review point p22, we simplified the figure and removed the subplot Fig 9b.

27. Figure 9 caption: "'*Expect'* shows the expected Larmor frequency **due to the set of the additional coil**." What does this mean?

This refers to the additional Helmholtz-like coil that was installed in the Halbach coil to shift the magnetic field by a known offset. The goal was to verify if the determined peaks in the spectrum corresponds to the NMR effect (and not e.g. to noise). This coil is shown in Fig. 4e. We have updated the figure caption accordingly.

28. Figure 9: Comment on the Hall sensor oiset. It seems consistently large. Is this a calibration issue?

There are several errors leading to this deviation:

- The Hall sensor is not calibrated, so according to the datasheet, an error of up to $|\Delta_{Hall}| \leq 2.5\%$ is possible in extreme cases (while $|\Delta_{Hall}| \approx 0.7\%$ is typically achieved).
- The Hall sensor is located approximately 15 mm below the center of the NMR sample and experiences a different field in this highly inhomogeneous field.
- Additionally, the offset coil causes a variable magnetic field that differs at the Hall sensor compared to the NMR sample itself (since the coil diameter is relatively small).

We recalculated the possible deviation of the measured magnetic field at the Hall sensor's location relative to the center of the NMR sample. This calculation is based on known magnetic field measurements from previous experiments and simulations of the additional offset coil. We updated Fig. 9a accordingly and plotted the remaining measurement tolerance additionally.

Discussion:

29. "Substantial cooling system" ← what is this referring to?

We wanted to reference to our section and design of the very large heat sinks. We have now deleted this section to stay within the word limit.

30. "an accurate matching of the Rx coil is not required, as it is only necessary for noise matching with the first LNA (not for noise free signal amplification by tuning)." ← unclear.

We have rephrased the sentence and made it clearer.

31. "the drift was exceptionally high, which likely prohibits any MR-imaging." ← how is it different from other Halbach-based scanners that have demonstrated imaging?

The recorded magnetic field drift is significantly higher than that of other low field MRI systems. This is due, in part, to the fact that our system has not been shimmed or thermally stabilized. Additionally, temperature differences have been introduced. We have now added references for comparison (new references 59 and 60).

32. No mention of future method for fieldmapping. Can the probe be moved around with a fieldmapping robot?

Yes, the sensor can be moved automatically. We have included a corresponding section with references.

33. "However, this would be possible with the identical sensor hardware using its hybrid mode" ← doesn't the hybrid mode still require an equally long polarization time?

Yes, that's right. We were referring to the called hybrid mode, but without pre-polarization. We've revised the sentence.

34. "the coils were mechanically designed so that the sample can be removed from the polarization coil and refilled. " ← The receive coil appears to be wrapped around the sample in a spherical shape. How is the sample removed from that configuration?

The entire inner assembly (sample container and the Rx coil wound around it) can be removed as a single unit. This can be seen in the rendering in Fig. 2a or the photo in Fig. 3a (vertical guide rails for the inner assembly are visible in the pre-polarization coil). Once this unit is removed, the sample container can be filled through the side using a syringe.

35. "the same order of magnitude as a voxel in LFMRI." ← what is the diameter of the sample? I'm not sure this is true. I believe a standard voxel size for low field scanner would be <0.1 ml of water.

We had originally referred to the DWI method described in the cited paper. Compared to this method, our sample size is about eight times larger. In fact, modern LFMRI devices using different sequences achieve significantly higher resolutions. We have now removed that sentence.

Methods:

36. Rx coil simulation workflow is difficult to follow—clarify base geometries.

We have revised the section on the Rx coil, in part in response to the comment Reviewer 2, Major, point2.

Reviewer 3

1. I co-reviewed this manuscript with one of the reviewers who provided the listed reports. This is part of the Communications Engineering initiative to facilitate training in peer review and to provide appropriate recognition for Early Career Researchers who co-review manuscripts.

Thank you for taking the time to review our work and provide your detailed comments in the other reviews. Also, we wish you success in your future career journey!

Editor

Dear Mr Povolni,

Thank you again for submitting your manuscript "Overcoming the Limitations of NMR Field Probes: A Novel Integrated Sensor Utilizing Pre-Polarization for (Ultra) Low Field MRI" to Communications Engineering. We have now received reports from 3 reviewers and, based on their comments, we have decided to invite a revision of your work again. You will find the reviewers' reports below. While they find your work of interest, they have raised important points which must be addressed in a revised manuscript.
To allow us to move forward with your work, we also ask that you edit your manuscript according to the table linked below. **Please read this document carefully as we will be unable to further assess your revised paper until these important points are addressed.**
* ′′ *
We hope to receive your revised paper within six weeks, but we understand that the revisions may take longer. Please let us know if you find that the revision process will take substantially more time.

We are committed to providing a fair and constructive peer-review process. Please do not hesitate to contact me if you have any questions or would like to discuss these revisions further. We look forward to seeing the revised manuscript and thank you for the opportunity to review your work.

Reviewer 1

Thank you for the detailed and constructive responses. I appreciate that the authors have substantially revised the introduction, added comparisons with other magnetic field measurement approaches, and clarified several technical limitations. These revisions improve the manuscript. From a reviewer's perspective, I would still encourage the authors to make the **central contribution more explicit and focused**. The strongest aspect of the work appears to be the engineering realization of an integrated NMR-based magnetic field sensor for low-field MRI-relevant conditions, rather than a broad demonstration across multiple MRI-related applications. A clearer emphasis on magnetic field measurement, system integration, and practical sensing performance would make the manuscript more convincing.

Thank you for the review of our novel submission and the feedback.

We agree that the central contribution of this work is the engineering realization of an integrated NMR-based magnetic field sensor for low-field MRI. The manuscript has therefore been revised to place greater emphasis on this aspect throughout.

In particular, the Introduction and Discussion were substantially rewritten to better highlight the motivation, the sensor concept, and its intended application as a high-precision magnetic field sensor. We further emphasize that the main advantage of the presented non-adiabatic excitation is that it enables NMR-based field measurements without requiring prior knowledge of the exact Larmor frequency or retuning of the RF hardware (e.g. the retuning of the Rx and Tx coils) when the magnetic field changes.

In addition, Section VII of the Results and the Supplementary Information (Section 10) were extensively expanded. These additions provide a more detailed analysis of the practical operating

limits of the current proof-of-concept implementation and are supported by additional simulations explaining the observed behavior at higher magnetic fields.

Reviewer 2

REVISED MANUSCRIPT: Overcoming the limitations of NMR Field Probes: A Novel Integrated Sensor Utilizing Pre-Polarization for (Ultra) Low Field MRI

Summary

This manuscript presents the development and validation of a new NMR field probe for measuring magnetic fields at low field strengths. The main technical innovation of this work is the use of SiC-nFETs in intentional avalanche mode, enabling high voltages and rapid current ramps for a non-adiabatic prepolarization switch off.

In the revision and rebuttal documents, the authors address our revision comments earnestly. The authors improved the structure of the manuscript, making significant improvements to the introduction section by removing extensive reference to the UN's SDGs, and instead addressing limitations of currently available (Hall-Effect sensors, NMR probes at high-field, etc.) field measurement systems.

Further, the author's make significant improvements to the presentation of data, including the inclusion of fitted spectra (Fig. 5, Fig. 9), confidence bands, and in-depth discussion of the statistical analysis and fitting run on data in figure captions. However, significant limitations remain in the manuscript. Though the author's do address comments regarding data quality and reliability of measurements, we find that their response is insufficient to convincingly support their conclusions. For example, Figure 9, detailing data collected with the NMR field probe at 45 mT, includes many 'missing' spectra which are not adequately explained, and which call into question the reliability and repeatability of field measurements acquired at the upper end of the proposed dynamic range of the field probe. This is additionally reflected in Figure 8, which details RF excitation at 1 mT, and Figures 6 & 7, which describe the gradient offset analysis.

Given these unresolved issues, the current dataset does not convincingly demonstrate the performance claimed across the full dynamic range of the field probe. Additional data acquisition, or a substantial re-evaluation of the claims and supporting analysis, is necessary before the conclusions can be considered adequately supported.

Thank you very for your thorough review of our revised manuscript. In the following, we will address your comments.

Major Issues Remaining:

1. **Writing and Structure:**

   Though the authors removed reference to SDGs to instead present current approaches to measuring magnetic fields at low field strengths, the motivation for their approach is still underdeveloped. The structure by which they break current measurement methods into HFMRI and LFMRI categories is confusing, and explicit limitations (i.e. dynamic range, cost, accuracy, precision) are not mentioned for existing approaches, which weakens the motivation for their novel approach.

We agree that the motivation for the presented sensor concept needed to be made more explicit and tried to implement it during the first revision. The main goal of this work is the development of a precise and robust NMR-based magnetic field sensor for low-field MRI applications, addressing limitations of existing approaches regarding accuracy, long-term stability, integration, and accessibility.

The revised Introduction has been reorganized to better motivate this technological gap. In particular, we have clarified the limitations of existing magnetic field measurement approaches and reduced the separation between high-field and low-field MRI systems. Conventional NMR-based sensors provide excellent accuracy but are often limited by hardware complexity, the need for resonant RF excitation, and limited integration possibilities. The presented excitation approach addresses these challenges by enabling NMR-based field measurements without requiring precise prior knowledge of the magnetic field.

The Introduction and Discussion have been extensively revised to emphasize the central contribution of the work, namely the realization of an integrated NMR-based field sensor for low-field MRI-relevant conditions (see also the answer to the Major point 1 of Reviewer 1).

2. **Data Quality and Convincingness of Conclusions:**

Given the level of detail in the raised point, we would like to address each paragraph separately below. However, for the sake of clarity, the revisions in the manuscript are referred to as "Rev2, Major, p2." for all 4 subpoints.

> Though the authors address our concerns in their rebuttal document, we find that their revisions were insufficient to assuage our concerns. The authors did a good job explaining why repeated measurements are difficult to achieve in their systems due to significant field drift, and in turn included confidence intervals and fits to demonstrate the ability of their probe to detect resonant peaks, which strengthens the presentation of their data. However, the quality of their data is unconvincing. Specifically, data presented in Figures 6, 7, 8, and 9 are noisy, inconsistent, and don't strongly follow fitted trends.

We thank you for recognizing our efforts to further explain the measured spectra.

We agree that several datasets exhibit noticeable noise, which is mainly related to the challenging operating conditions of the presented proof-of-concept implementation. However, the intended functionality of the (magnetic) sensor is the determination of the local magnetic field via the Larmor frequency, rather than achieving maximum possible SNR under all conditions.

During the sensor development, the primary objective was therefore to obtain sufficient signal quality for reliable frequency extraction, while accepting that some excitation-related disturbances and technical limitations remain in the current implementation. The observed variations are not caused by a fundamental limitation of the sensing principle, but originate from practical aspects of the excitation and measurement hardware, which are now discussed in more detail in the revised manuscript and Supplementary Information.

To further address these concerns, we have improved the data processing, revised the presentation of the figures, and added additional analysis and simulations explaining the observed deviations from ideal trends. These additions clarify the current limitations while demonstrating that the sensor concept is functional across the investigated magnetic field range.

> Figure 6, while greatly improved by the inclusion of confidence intervals, still demonstrates weak adherence to linear and exponential trends without sufficient explanation. Figure 7, which details the gradient offset analysis, is generally confusing. The use of the NMR probe for measurement of gradient fields is not strongly motivated in the manuscript, and the authors choice to move the field probe for different field strength measurements is confusing, as the reliability of the system (i.e., does the probe correctly localize gradient offset across different field strengths?) isn't demonstrated.

Thanks for these helpful comments.

Regarding Figure 6:

We agree that the measured amplitudes exhibit noticeable scatter around the fitted linear and exponential trends. We have therefore expanded the description on the result in the manuscript to explain that these deviations most likely originate from small fluctuations of the pre-polarization generator operating point and timing uncertainties in the excitation and receive chain, while the FWHM remains nearly constant over the investigated parameter range. Since these effects are analyzed in detail later in the manuscript regarding the (former) figure 9 (now figure 8) and the measurements at $B_0 = 45mT$, we refer the reader to the corresponding discussion and the expanded SI (SI chapter 10).

Regarding Figure 7:

We appreciate that the original presentation could be interpreted as a characterization of the gradient system. This was not the intention. The purpose of this experiment was to demonstrate that the developed NMR field sensor can reliably resolve very small magnetic field and frequency variations, which could be expected during MRI operation, rather than to characterize the gradients themselves. The gradient-induced frequency shifts were used merely as a convenient and well-defined test magnetic field, and the estimated sensor position served only as an illustrative example of a potential application. To avoid this confusion and to reduce the manuscript length, we substantially shortened this section in the main text and moved the detailed analysis to the SI chapter 7, where it is available for interested readers.

> Figure 8, which investigates use of RF excitation at 1 mT to motivate the operating range of the scanner, ultimately includes very messy data, in which spectra cannot be extracted at many prepolarization field strengths. Further, the FWHM and Amplitude graphs to do not demonstrate convincing trends. While we appreciate that the authors include this data to represent NMR probe operation at the 'worse-case', the data is ultimately unconvincing and weakens their assertion that their probe can operate within the dynamic range from 1 mT – 45 mT.

We thank the reviewer for pointing out the unclear presentation of the 1 mT RF excitation data. We identified an error in the preprocessing pipeline used for these measurements. As described in SI chapter 5 (Figure S3) and in the method section, an apodization filter is directly applied during the import of the raw time domain data. However, for the 1 mT measurements with an conventional resonant RF excitation, a typographical error in the processing script resulted in incorrect filter limits, effectively disabling the intended apodization. After correcting the preprocessing parameters, the spectra exhibit substantially reduced noise and the extracted signal characteristics become more reliable.

We have updated the corresponding data and figures accordingly.

It should be noted that for pre-polarization fields below Bp < 26 mT, no reliable NMR signal is obtained with the four averages used in this experiment, which is consistent with the reduced polarization level. The complete dataset, including the updated spectra, is now provided in SI Chapter 8.

We appreciate the reviewer's comment, as it helped us identify this processing error and improve the clarity and reliability of the presented results.

> This is further reflected in Figure 9, which includes generally very noisy data. Though figure presentation is greatly improved, the data quality is still unconvincing, particularly when 'missing spectra' appear. A significant amount of this data has no detectable spectral peak, and other data sets are fit so austerely that its unconvincing. Ultimately, an expanded analysis which examines the performance of their probe at more intermediate fields would benefit the authors. The inclusion of data that is primarily acquired at the limits of the proposed dynamic

range should be accompanied by data on intermediate fields that may better demonstrate the SNR, reliability, and accuracy of their measurements.

We thank the reviewer for this important comment. We agree that the original presentation of the $B_0 = 45mT$ measurements contained spectra with insufficient signal quality, particularly for cases where no detectable peak was obtained. The main reason for the observed variability is the small transverse magnetization remaining after the non-adiabatic excitation process. This is expected for the chosen operating point, as the adiabaticity condition described by Tannús and Garwood is almost completely fulfilled for the developed sensor at $B_0 = 45mT$. As previously discussed in SI Chapter 3, this leads to a highly dynamic magnetization trajectory during the switch off where only a small residual transverse magnetization is remained after its finish.

Furthermore, the signal amplitude is affected by timing variations in the complete excitation and receive chain. Small timing uncertainties originating from the low-voltage electronics, including the gate driver, trigger filtering stages, and opto-isolating protection of the LNA, become relevant due to the short T_2^* caused by the unshimmed permanent magnet. Consequently, parts of the FID can already decay before signal acquisition begins.

Motivated by the reviewer's comment, we performed an extended analysis of the excitation process in this area and investigated the origin of the signal variations in more detail. The Bloch simulations were extended by including the experimentally observed avalanche voltage evolution rather than assuming an ideal rectangular avalanche pulse (and therefore perfect linear current ramp due to $U = -Ldi/dt$). This results in a non-perfectly linear current ramp and significantly modifies the magnetization trajectory due to the high dynamics of the rotating and accelerated magnetization vector (e.g. period of the larmor oscilation is about $t \approx 540ns$, current ramp $t_{pR} > 1\mu s$). The simulations demonstrate that really even small variations of the avalanche transient response, particularly during avalanche termination, can strongly influence the resulting transverse magnetization. For example, variations of only $50ns$ in the avalanche termination timing (called corner time in the analysis) can change the simulated signal amplitude over the full range from negative to positive values. Since avalanche breakdown is intrinsically stochastic and depends on internal semiconductor processes, these variations provide a plausible explanation for the observed signal fluctuations and occasional missing spectra.

Nevertheless, the sensor itself remains fully functional, as at least one valid NMR spectrum was obtained for every investigated operating condition. To better reflect this behavior, we revised the presentation of the main manuscript by focusing Figure 9 (now Figure 8 in the new manuscript) on representative successful FID measurements and moved the complete dataset, including spectra without detectable peaks, to SI Chapter 10.

A direct electric measurement of the avalanche voltage during the switch off would provide further insight into the switching dynamics. However, as discussed in the expanded SI Chapter 10, this requires specialized high-voltage differential probes with very high bandwidth and minimal parasitic loading, as the measurement itself may perturb the avalanche process. Therefore, we retained the previously established galvanically isolated pickup-coil measurement approach and complemented it with the extended simulation analysis.

In addition to the revised Results section, we expanded the corresponding Discussion and SI Chapter 10 to include the detailed analysis of avalanche-induced signal variations.

We appreciate the reviewer's suggestion, which significantly improved the interpretation and presentation of these measurements.

Specific Comments:

1. Introduction

1.1. Don't need to include "consistent with various studies, including more recent publications", as this implies that the findings in 6 are outdated.

Our intention was not to imply that the findings reported in Ref. 6 are outdated. Rather, we intended to emphasize that the reported disparity in MRI availability is consistently supported by subsequent studies. To avoid this uncertainty, we have revised the wording accordingly.

1.2. Be careful with the idea that low-field scanners are "significantly less expensive", as considerable attention has been paid to the cost of commercially available low-field systems. Consider changing to "can be significantly less expensive".

This is a valid point regarding the cost of current commercial low-field MRI systems. However, we refer here to the fundamental cost advantages resulting from reduced hardware complexity and infrastructure requirements compared with conventional high-field MRI systems. Even though commercial low-field systems may be more expensive than initially anticipated, they remain orders of magnitude more cost efficient and less demanding in terms of infrastructure, particularly due to the absence of superconducting magnets and cryogenic operation. Therefore, we decided to retain the original wording.

1.3. The wording here is awkward: "require minimal infrastructure because, e.g., cryogenic cooling is not necessary". I understand that you are trying to provide an example of why operational cost is reduced, but the use of e.g. is clunky without saving that much space.

The wording has been revised to improve readability.

1.4. Change to "recent developments have demonstrated the feasibility of in-vivo imaging with permanent-magnet-based MRI systems…."

The wording has been revised.

1.5. The discussion of permanent magnet-based designs vs. electromagnets seems out of place. As this paper focuses on implementation of the field probe, the discussion of trade-offs between electromagnet and permanent magnet systems seems unnecessary. Further, perhaps the most significant limitation of electromagnets has to do with system power and cooling requirements, which are more problematic in low-resource settings.

The section has been revised.

1.6. You state "a fundamental problem for the entire LFMRI research is the lack of highly precise magnetic field sensors:…", but following the colon you go onto describe why sensors are needed in general, not the lack of highly precise sensors.

The wording has been revised (see also answer to Rev1, Major, p1 and Rev2, Major, p1).

1.7. The comparison between HFMRI and LFMRI field mapping seems like an unnecessary distinction. Further, reference 31 is using a self-built array of NMR sensors for a LFMRI system, not a HFMRI system. Additionally, the idea that access to commercial systems is limited due to "enormous high cost" that "may be more than the cost of LFMRI alone" is not well supported/referenced. Additionally, you did not address the limitations of the self-built systems (30,31) referenced, so this conclusion (inaccessible due to cost) is not applicable to all HFMRI field measurement systems.

The section has been revised, also according to other points raised.

1.8. In general, it seems like the most apt comparison for your system are commercially available 3-axis Hall Effect gaussmeters. I would limit the scope of your discussion to these devices to improve clarity.

It is a valid point that 3-axis Hall-effect teslameters are commonly used for characterization of permanent-magnet-based LFMRI systems. However, their accuracy is typically insufficient for high-precision field characterization. In our experience, the practical resolution is rarely better than 0.1%, corresponding to approximately 1000 ppm, and even excellent Hall sensors require a valid calibration. In contrast, NMR-based sensors provide substantially higher precision through direct frequency-based field determination and do not require external calibration. Therefore, we decided to retain the broader comparison to emphasize this fundamental advantage of NMR-based field sensors. Also, in compliance with the points raised by Reviewer 1.

1.9. Overall, the claim that “a fundamental problem for the entire LFMRI research is the lack of highly precise magnetic field sensors” is a substantial claim that isn’t well supported or motivated by the additional background you have included. Though you state that NMR probes are the most accurate, you haven’t necessarily motivated that Hall effect sensors are not “highly precise” or that their precision is completely unsuitable for use in measuring LFMRI magnets, and the idea that other systems are extremely expensive isn’t well motivated either.

The reviewer raises a valid point regarding the broad wording of this statement. We agree that Hall sensors are widely used for initial characterization and shimming of permanent-magnet-based LFMRI systems. However, their accuracy, long-term stability, temperature vulnerability, and calibration requirements become limiting factors when precise field monitoring or reproducible measurements are required.

NMR-based sensors provide superior accuracy and intrinsic frequency-based field determination, which is why they are widely used for high-precision magnetic field characterization and are considered as the backbone of ultra-precise robust magnetic field measurement. A detailed comparison of all available magnetic field sensing technologies is beyond the scope of this introduction; comprehensive reviews are available in the literature (e.g. the referenced overview by Bottura and Henrichsen, 2004). We have adapted the wording to more specifically emphasize the need for integrated, high-precision field sensors for LFMRI applications.

1.10. You state that a large challenge is the large dynamic range needed for measurement of the field during construction, but you don’t compare the dynamic range of existing field measurement sensors. For example, Hall effect gaussmeters often have very high dynamic range, so a priori knowledge of the field isn’t required for these sensors either, and they are commonly used during construction to catch errors (i.e., mapping of individual rings during construction).

Our intention was not to imply that Hall sensor based systems cannot be used for magnet mapping or construction quality control. Or that they could be replaced by the developed sensor.

Instead, the challenge addressed here is the precise monitoring of small temporal and spatial field variations during operation, where long-term stability, calibration, and accuracy become critical. For example, temperature-dependent drifts of permanent magnets can introduce frequency shifts that significantly affect MRI operation and may prevent the use of more advanced sequences. However, they are almost not measurable with conventional hall sensors.

We have revised the text to clarify that the required capability extends beyond initial field mapping toward precise, dynamic field monitoring during LFMRI operation.

1.11. Ppm is typically used in the context of describing field variation, not relative/remaining field strength. The idea that only 280 ppm of the signal remains at 50 mT is non-standard and confusing.

It is true that ppm is typically used in MRI to describe the inhomogeneity of the magnetic field. However, it is also a unit of measurement used to describe very small amounts of a substance within a larger whole. In this context, it makes sense to use it here to illustrate the significantly lower polarization in low-field conditions (and thus the challenge posed by low signal intensity).

1.12. Good inclusion of reference to geophysical applications and the specific innovation of your sensor (3x faster ramp).

Thank you.

2. Results:

2.1. Non-Adiabatic Switch Off the Pre-Polarization Field:

2.1.1. Again, the inclusion of Eq. 2.1, though you state that a more detailed analysis is provided in the supplementary information, is confusing and unnecessary. This equation does not convincingly and clearly demonstrate the point that signal (U) is maximized with a large Bp and a short ramp time. Perhaps a different equation, or a more qualitative discussion, can better highlight the need for faster ramp times to maintain the NMR signal, and thus motivate your use of the SiC-nFET in intentional avalanche breakdown.

The purpose of Eq. 2.1 is not to directly describe the signal amplitude dependence on B_p and ramp time, but to introduce the adiabaticity condition described by Tannús and Garwood. This condition determines whether the magnetization follows the effective field adiabatically and returns to equilibrium after the excitation process. We agree that the equation is not commonly used and may appear complex without further explanation. Therefore, we have added a short clarification in the SI, while referring to the original literature for a detailed derivation.

We decided to retain Eq. 2.1 in the introduction because exceeding this commonly accepted adiabatic limit is a central aspect of our approach. The measurements at $B_0 = 45mT$ demonstrate that this boundary can be surpassed using the developed avalanche-based excitation scheme. A qualitative discussion alone would not sufficiently highlight the physical regime that motivates the use of SiC-nFET avalanche switching and the resulting high field ramps.

2.1.2. Nice, clear discussion of the novelty of using the SiC-nFET in intentional avalanche break down. Also, nice mention of the aging tests.

Thank you!

2.1.3. I would still like additional clarification about the operating range of this sensor. Under 5 mT, you state that the adiabatic condition is significantly violated for tested Bp under 70 mT. However, most low-field systems used in-vivo are above 5 mT, closer to 50 mT and above. Although you state that a weak transverse magnetization remains visible in simulation at 50 mT if the “sensor concept is designed accordingly”, I don’t find this particularly convincing when trying to demonstrate the feasibility/appropriateness of this NMR sensor over a traditional hall-effect sensor at low-field. In your rebuttal, you mention that you have tested the systems extremes, but again, an operating point above 50 mT isn’t really an extreme, and is rather the norm, for most in-vivo low-field imaging systems.

We have clarified throughout the manuscript that the presented sensor is not intended to replace established Hall-effect-based characterization methods, but rather to provide an additional high-precision NMR-based field measurement capability. Hall sensors remain highly valuable for initial magnet characterization and are therefore also included in our hardware setup to simplify spectral

analysis. The motivation for this work is to demonstrate, for the first time, that controlled non-adiabatic excitation using SiC avalanche switching can generate measurable NMR signals at low-field MRI-relevant conditions with the intrinsic accuracy of NMR-based sensing.

We agree that the presented hardware is not yet optimized for all future in-vivo low-field MRI applications, particularly around 50 mT and above. This work should therefore be considered a proof-of-principle demonstration of the excitation and sensing concept. The experimental results confirm operation from 1 mT to 45 mT, and simulations show that operation at higher fields is feasible when the sensor parameters are adapted accordingly, for example by optimizing the pre-polarization coil. The required hardware adaptations and further optimization of the avalanche circuit represent important directions for future developments. We have revised the manuscript to better emphasize these aspects and clarify the intended scope of the demonstrated operating range.

2.2. Design of the sample, polarization coil, and receive coil for maximum sensitivity:

2.2.1. Although you have removed the sentence “on the same order of magnitude as a voxel in LFMRI”, the volume of the sample (1.2-1.5 mL) still seems very large and inherently limiting for the spatial resolution of this sensor. If you want high spatial resolution in your field map (which you generally do), then a volume of 1.5 ml represents a 15x15x15 mm voxel, which seems too large. Please additionally clarify or justify this.

A clarifying sentence was added

2.2.2. You still use the term “thermal polarization”

Nice catch! It was changed

2.2.3. I would still like a more explicit explanation of the different operating modes and their use cases for this sensor. The novelty of this sensor is the rapid NA switch-off facilitated by the intentional avalanche breakdown of the SiC-nFET, but the NA switch-off is only advantageous up to 26 mT. Beyond that, conventional RF excitation generates higher SNR, and you need to use a different Rx coil set up. Although you mention in your rebuttal that the receive coil still operates well +/- 24% B0 variation, this doesn’t really address the fact that the proposed sensor only seems the most advantageous for ULFMRI, which isn’t particularly common for in-vivo imaging in low-resource settings.

An important point regarding the different operating modes of the presented sensor is raised. The non-adiabatic switch-off mode indeed provides the largest advantage when the adiabatic condition is violated, where it enables excitation without prior knowledge of the exact Larmor frequency and therefore avoids the need for precise RF matching and pulse calibration. However, the presented sensor concept is not limited to this excitation mode. The hybrid design also supports conventional RF excitation with adiabatic pre-polarization, which provides higher signal amplitudes at higher magnetic fields.

The stated $B_0 = 26mT$ limit refers to the achievable EMF after the non-adiabatic excitation for the current hardware configuration and is not a fundamental physical limit. Furthermore, for a field sensor, the absolute signal amplitude is not the only relevant parameter, as long as the spectral linewidth enables the required frequency accuracy. The main advantage of the non-adiabatic approach is the rapid and robust excitation without requiring precise prior field knowledge (as described in the introduction and discussion).

In the presented proof-of-concept, the hardware was not further optimized for higher-field operation (which e.g. also needs adaption on the Rx chain and the sampling to handle higher frequencies); future developments may adapt the pre-polarization coil, receive chain, and avalanche circuit for specific MRI field regimes.

We have clarified the different operating modes and their intended applications in the revised manuscript.

2.3. NMR Measurements in Ultra low field for B0< 5 mT:

2.3.1. Figure 6: Even though the authors responded and clarified why they have chosen to perform only a single measurement due to the drift in the main B0 field, without averages the fits look relatively unconvincing still. Its hard to appreciate the stability of the system from this data.

The detailed reasoning is addressed in the response to Major Comment 2. The revised manuscript now provides additional discussion and analyzed data regarding the observed variations and the corresponding limitations of the measurement approach.

2.3.2. I am still confused by this analysis. The clarification from the author in the rebuttal helped to demonstrate the purpose of the experiment (to determine the positional offsets of the gradients relative to each other), but I don't think this is well communicated in the revised manuscript itself. Further, I am confused about the evaluation over 3 different field strengths. Did you deliberately move the probe each time (1,3,5 mT), and for what purpose? It would be useful to also explicitly NOT move the probe and calculate/fit the offsets at different strengths to show the accuracy of the sensor across this range of field strengths.

The detailed motivation and limitations of this measurement are addressed in the response to Major point 2.

To clarify, the frontend was not moved during each individual gradient measurement. For a fixed B_0 setup , the three gradients were measured sequentially with the same sensor position. Afterwards, the frontend was exchanged and the measurement was repeated at the next field strength.

The purpose of this experiment was not to characterize the gradient system itself, but to demonstrate the ability of the sensor to resolve small magnetic field variations and frequency shifts. Since the current frontend design is not optimized for operation over the complete field range with identical performance, a more systematic investigation of gradient offsets and sensor accuracy across different field strengths is beyond the scope of this work and will be addressed in future studies. Due to the limited relevance for the main message of the manuscript, the detailed gradient analysis has been moved to the SI chapter 7.

2.4. Hybrid measurement with RF excitation in Ultra-low field:

2.4.1. Figure 8: I appreciate what the author's included in their rebuttal about wanting to show the acceptable performance of the hybrid excitation even at 1 mT B0. However, I am still confused by the lack of trend in (b) and (c). If the amplitude, for example, doesn't increase monotonically with increasing Bp, then is this performance suitable? Ultimately, I am confused as to why the authors are demonstrating the adiabatic hybrid performance at 1 mT when the performance of the NA switch off is far superior.

The detailed response regarding Figure 8 is provided in the corresponding major point 2. During the revision, we identified an error in the preprocessing routine for these data, where the apodization filter was incorrectly applied due to a parameter mismatch. After correcting the filter parameters, the spectra exhibit substantially improved quality and the extracted parameters show a more consistent behavior.

The hybrid excitation measurement was included to demonstrate that the sensor concept is not limited to non-adiabatic excitation, but can also operate with conventional RF excitation. The revised data show the expected signal increase with increasing pre-polarization field, while the FWHM exhibits a slight increase at higher pre-polarization fields, likely due to small dephasing effects during the adiabatic switch-off. Additional discussion has been added to clarify these observations. The purpose of this experiment is therefore not to outperform the non-adiabatic excitation mode, but to demonstrate the flexibility of the hybrid sensor concept.

2.5. NMR Measurements in low-field at B0 = 45 mT:

2.5.1. *"Due to the significant frequency drift, averaging the spectra is not recommended as it broadens the measured peak and consequently reduce SNR. Moreover, averaging is not necessary, as SNR is sufficient for peak detection and a robust spectral fit in a single measurement already."*

1) Please clarify what is meant by "frequency drift" in this context. Figure 9 includes an intentionally applied frequency offset; is this offset intended to represent the drift referred to here?

The term "frequency drift" in this context does not refer to the intentionally applied frequency offset shown in Figure 9 (now Figure 8). The purpose of this measurement was to demonstrate that the sensor can detect temporal frequency variations of a permanent-magnet-based system. Therefore, three individual FIDs were acquired sequentially using identical settings, and the resulting frequency differences were evaluated as field drift. Due to this drift the individual measurements were not averaged.

The intentionally applied frequency offset was generated by the additional coil and served only as a controlled frequency shift for evaluation of the sensor response.

2) The statement that the SNR is sufficient for robust fitting from a single measurement does not appear to be supported by the data shown in Figure 9, where many spectra appear to have low SNR and poorly distinguishable peaks.

The detailed analysis and clarification regarding Figure 9 (now figure 8) are provided in the response to Major point 2. In the revised manuscript, we have expanded the discussion of the reduced signal quality and added additional simulations to investigate the origin of the observed variations. The missing or weak spectra are primarily related to the highly dynamic and stochastic avalanche-based excitation process and the resulting variations in the residual transverse magnetization.

2.5.2.Figure 9:

1) Without averaging, the data at 45 mT remain unconvincing. In many instances, the peak corresponding to the B0 field appears to be at or near the noise floor. Furthermore, fitted spectra are not shown for many measurements. The authors suggest that the missing spectra could be due to switching of the prepolarization generator; however, given the large number of measurements in which no peak is detectable, this raises concerns regarding the reliability and repeatability of the system at the upper end of its proposed operating range.

The detailed analysis and clarification are provided in the response to Major point 2. The revised manuscript now focuses on the successful spectra, while the full dataset including missing spectra is provided in the SI.

2) Several displayed fits appear difficult to justify based on the presented data (for example, S4 of repetition 2). Please clarify the fitting procedure, including any fitting constraints and criteria used to identify valid peaks.

The fitting procedure is described in detail in SI Chapter 5. The spectra are fitted in the integral domain. The only constraint applied during fitting is the predefined frequency range in which the resonance peak is expected. For spectra with very low SNR, the fit quality naturally decreases and can fail if the peak is hard to be distinguishable from the noise level. No additional constraints were introduced to artificially force peak detection. The chosen simple fitting approach was intentionally selected to remain suitable for future implementation on embedded hardware, such as a microcontroller, where computational simplicity is important.

3) The tolerance band of the Hall sensor also appears quite large. Please provide additional explanation regarding the origin of this tolerance band and how it should be interpreted relative to the NMR-based measurements.

As answered in the previous revision there are several errors leading to the deviation of the hall sensor value:

- The Hall sensor is not calibrated, so according to the datasheet, an error of up to $|\Delta_{Hall}| \leq 2.5\%$ is possible in extreme cases (while $|\Delta_{Hall}| \approx 0.7\%$ is typically achieved).
- The Hall sensor is located approximately 15 mm below the center of the NMR sample and experiences a different field in this highly inhomogeneous field.
- Additionally, the offset coil causes a variable magnetic field that differs at the Hall sensor compared to the NMR sample itself (since the coil diameter is relatively small).

We recalculated the possible deviation of the measured magnetic field at the Hall sensor's location relative to the center of the NMR sample. This calculation is based on known magnetic field measurements from previous experiments and simulations of the additional offset coil.

To consider these errors (esp. to the different position), a relatively large error was considered. In future developments, the Hall sensor can be moved to the same position as the NMR sensor using a positioning robot. However, this is not necessary, since the Hall sensor is used only as a reference to simplify the spectral analysis.

4) Overall, the 45 mT results, which the authors describe as representing the upper limit of operation, do not convincingly demonstrate reliable probe performance at this field strength. Given that the manuscript motivates the work in part through applications in low-field MRI, where operating fields are often on the order of 45 mT or higher, this limitation should either be addressed with additional evidence or more clearly reflected in the claims.

The detailed explanation regarding the measurements is provided in the response to Major point 2. We have revised the manuscript to better reflect the current status of the sensor concept as a proof-of-principle demonstration. These measurements confirm that the presented excitation and detection approach can generate measurable NMR signals in this field regime, while the observed variations originate from the current avalanche-based excitation implementation and not from a fundamental limitation of the sensing principle. Additional simulations and discussion have been added to clarify these effects and to define future optimization steps required for improved robustness at higher fields.

2.5.3. Figure 9 caption states: "The vertical lines correspond to the measured value from the embedded Hall sensor (with the tolerances of the used sensor added), to the mean Larmor frequency of the three measurements, and to the expected Larmor frequency due to the additional offset coil (see Figure 4e)." However, three distinct vertical lines are not visible in all subplots. In addition, the legend indicates dotted lines, whereas the caption refers to vertical lines without clearly identifying their styles. It is also unclear whether the legend applies to both panels or only to Figure 9b, where dotted lines are present.

The figure has been simplified and revised to improve clarity. The three reference lines are now consistently shown and their styles are explicitly defined in the caption. The lines are dashed (not dotted), and the legend has been updated to clarify that it applies to both panels.

3. Discussion:

3.1. While the authors have improved the discussion, I remain unconvinced regarding the utility of the proposed system across the full claimed operating range of 1–50 mT. At the low end

of this range (1 mT), the hybrid approach does not appear particularly robust, while at the high end (45–50 mT), the performance of the NA prepolarization approach appears limited (see comments regarding Figure 9). Ultimately, the challenges observed at the limits of the proposed dynamic range should be discussed in the context of existing field measurement technologies, such as Hall-effect sensors, rather than solely in comparison with other NMR-based sensors that employ adiabatic switch-off at higher field strengths.

The detailed discussions regarding the different operating regimes and limitations are provided in the responses to the corresponding major comments. We have revised the manuscript to better clarify that the presented system is a proof-of-principle demonstration of an integrated NMR-based field sensor rather than a fully optimized solution for every magnetic field strength.

The purpose of the presented operating range from 1–50 mT is to demonstrate the flexibility of the sensor concept across the relevant regimes of modern low-field MRI, including ultra-low-field electromagnet systems and permanent-magnet-based head MRI. At 1 mT, the hybrid excitation demonstrates that the sensor can operate with conventional RF excitation. At 45 mT, the non-adiabatic excitation demonstrates the feasibility of controlled SiC avalanche-based excitation in a field regime where the adiabatic condition is generally fulfilled, although further optimization of the excitation circuit is required.

We have additionally clarified the comparison with existing field measurement technologies. The proposed sensor is not intended to replace Hall-effect sensors for initial magnet characterization, but to provide an additional high-precision, NMR-based measurement capability where frequency-based field determination, long-term stability, and precise monitoring of small field variations are required. Future hardware optimization will be needed to adapt the sensor to specific MRI field regimes and applications.

Reviewer 3

I co-reviewed this manuscript with one of the reviewers who provided the listed reports. This is part of the Communications Engineering initiative to facilitate training in peer review and to provide appropriate recognition for Early Career Researchers who co-review manuscripts.

Thank you for taking the time to review our work and provide your detailed comments in the other reviews. We also wish you great success in your future academic career!

Review: "Overcoming the limitations of NMR Field Probes: A Novel Integrated Sensor 4 Utilizing Pre-Polarization for (Ultra) Low Field MRI"

Summary:

This manuscript presents the development and validation of a new NMR field probe designed for low-field MRI applications. It uses a fast non-adiabatic switch-off of a pre-polarization field to generate transverse magnetization without requiring prior knowledge of the B0 field. The main innovation is in the design of the pre-polarization system which uses a high-voltage silicon carbide (SiC) device in controlled avalanche breakdown to generate a high voltage across the coil and enable a fast current ramp down.

It is appreciated that the system is demonstrated across a wide field range (1 mT–45 mT) in an electromagnet ULFMRI and a Halbach LFMRI. The work emphasizes low cost, modularity, and open-hardware dissemination to support MRI development in resource-constrained settings.

The technical innovation is strong and relevant, but substantial improvements in writing, hardware description, data quality, and comparative validation are required for the work to fully support its claims and achieve its potential impact.

Major issues:

1. Writing and Structure:
    a. The Introduction is overly long and written and the content is not totally appropriate for a technical instrumentation paper. Extended discussion of global development themes and SDGs, while well intentioned, distracts from the core engineering contribution. This section should be condensed and refocused on the technical gap in low-field NMR field sensing and how this work addresses it.
    b. The manuscript would benefit from editing to make concepts more clear and concise. There are also numerous single-sentence paragraphs that disrupt the narrative flow.
2. Receive coil description: the receive coil design and geometry and tuning/matching procedure are not clearly explained, despite being central to probe sensitivity and bandwidth. Greater detail (descriptive and perhaps a figure) would be necessary for reproducibility.
3. Data Quality and Convincingness of Conclusions: A major concern is the quality and cleanliness of the experimental data. Several datasets appear noisy or low in SNR, which weakens the strength of the conclusions. In some cases, noise is attributed to laboratory interference, yet the manuscript also states that RF

shielding at these frequencies is relatively easy to implement. Many results rely on single acquisitions without averaging or uncertainty analysis.

The authors argue that averaging is difficult due to magnetic field drift. While drift is clearly present, averaging nonetheless seems necessary to convincingly demonstrate sensitivity and repeatability. If thermal effects from the pre-polarization or offset coils are driving drift, this could potentially be mitigated (or at least discussed) for example, by operating at a thermal steady state, increasing duty cycle stability, or implementing drift correction.

4. Comparison to Standard Methods: Finally, the comparative advantage over conventional NMR probes should be more critically examined. While the “no prior field knowledge” excitation is valuable, the receive coil still requires tuning/matching. In many systems, the same coil infrastructure could support broadband or frequency-swept transmit excitation, which partially offsets the claimed operational advantage. A more balanced comparison to standard probes would strengthen the manuscript’s positioning.

   Overall, I believe the data should be made cleaner through improved acquisition strategy, averaging, and/or post-processing. Although the method is scientifically very interesting, it is not convincing that there is a clear advantage over basic NMR field-probe based on the data quality presented.

Specific Comments

Introduction:

1. Sections discussing SDGs (lines ~43–54) are not well worded and extend beyond what is necessary to motivate the work. Consider condensing (if kept).
2. The hydrocephalus example (lines ~60–62) needs clarification and supporting citation (if kept).
3. Discussion of “brain drain” and local job markets (lines ~74–85) feels out of scope for a hardware instrumentation paper.
4. When discussing signal scaling (e.g., line ~109), clearer quantitative formatting (percentages or scientific notation) is recommended.
5. (111-113) Expand comparison to existing field probes/gaussmeters (e.g. Metrolabs, Lakeshore, etc): What dynamic ranges do they cover? Why are they insufficient during magnet construction?

Results:

6. Equation (2.1) is key but difficult to interpret and not well explained in the text.

7. Clarify why the adiabatic condition is easier to violate at lower fields.
8. Clarify notation: tp is used for both switch-off time (ns) and polarization duration (s).
9. Fig. 1b appears truncated (precession circle cut off).
10. Fig. 1e is difficult to interpret, but reverse-current across avalanche cycles appear quite variable.
11. The intentional avalanche operation of SiC-nFETs is a strong novelty. This should be emphasized quantitatively (ramp rates vs prior methods).
12. Fig. 2a suggests a spherical winding encasing the sample, whereas earlier figure implies perpendicular solenoid – please clarify this.
13. Provide contour scales and inhomogeneity metrics in Fig. 2b.
14. Fig. 2e plot and caption are generally confusing. For example, why do the simulations have error bars?
15. (349-351) "The implemented design offers the possibility of being easily adjusted for high B0 resolution (strong Bp, long tp, long TR), for high temporal resolution (strong Bp, short tp, short TR), or for energy efficient long-term drift measurements (weak Bp, long tp, long TR)". This is an interesting statement – please elaborate, particularly on the "high resolution" part – what resolution are you referring to and why does long tp and TR help – is this just related to the T1?
16. The simulated crossover (~26 mT) where RF excitation surpasses non-adiabatic SNR is an important result—highlight more explicitly.
17. Figure 6: Linear and exponential fits are not convincing. Only single spectra were acquired, can averaging be done? Also, error bars are recommended.
18. Figure 7: This figure is confusing. Please clarify the goal and procedure of the gradient evaluation. Are you trying to show gradient strength or linearity, frequency drift, or estimate position of the probe? At first glance, one would assume that the x-axis is showing the probe position, but it seems that the gradient strength is being varied? If so, it would be more intuitive to show results as a function of current instead of G. Why are the results so different for different B0s? The data is also not clean and the fit it is not good. Error bars should be added.
19. Section vi: "Testing at this low-field strength is considered a critical challenge to the sensor concept, since **matching of the Rx** and Tx coils is difficult" ← isn't the matching of the Rx coil equally challenging the NA method?
20. Section vi: Why is the term "thermal polarization" used? Isn't the polarization physics the same for the "normal" B0 polarization and the prepolarization?
21. Figure 8: Averaging and/or denoising is needed here. The fidelity of these measurements is not good. There is not a compelling trend in b and c.
22. Section vii: "resulting in an averaged frequency drift of Δf=835Hz". A drift of 835Hz every 29s seems extreme. Is there significant heating from the polarization coil that is causing this?
23. Section vii: "Due to the very short T_2^*, the resolution of the B_0 mapping is limited in this unshimmed case". Specify what is meant by "resolution" here.

24. Section vii: There is a comparison to Hall sensors here. I assumed different sensors have different dynamic ranges and resolutions. For example you could compare to some commercial off-the-shelf options (e.g. from Metrolabs).
25. Figure 9a: This data is not robust and it is displayed in a way that is hard to read/intepret. It seems odd that the x-axis is in kHz instead of MHz. There is no way to tell see the drift in these plots because the x-axes are very compressed. Perhaps overlaying the measurements in 3 plots would be more compelling? In general, I think higher fidelity measurements could be made a basic NMR experiment using a broadband Tx coil and frequency swepted pulses.
26. Figure 9b: unclear what you are trying to show here.
27. Figure 9 caption: "*'Expect'* shows the expected Larmor frequency **due to the set of the additional coil."** What does this mean?
28. Figure 9: Comment on the Hall sensor offset. It seems consistently large. Is this a calibration issue?

Discussion:

29. "Substantial cooling system" ← what is this referring to?
30. "an accurate matching of the Rx coil is not required, as it is only necessary for noise matching with the first LNA (not for noise free signal amplification by tuning)." ← unclear.
31. "the drift was exceptionally high, which likely prohibits any MR-imaging." ← how is it different from other Halbach-based scanners that have demonstrated imaging?
32. No mention of future method for fieldmapping. Can the probe be moved around with a fieldmapping robot?
33. "However, this would be possible with the identical sensor hardware using its hybrid mode" ← doesn't the hybrid mode still require an equally long polarization time?
34. "the coils were mechanically designed so that the sample can be removed from the polarization coil and refilled. " ← The receive coil appears to be wrapped around the sample in a spherical shape. How is the sample removed from that configuration?
35. "the same order of magnitude as a voxel in LFMRI." ← what is the diameter of the sample? I'm not sure this is true. I believe a standard voxel size for low field scanner would be <0.1 ml of water.

Methods:

36. Rx coil simulation workflow is difficult to follow—clarify base geometries.

REVISED MANUSCRIPT: Overcoming the limitations of NMR Field Probes: A Novel Integrated Sensor Utilizing Pre-Polarization for (Ultra) Low Field MRI

Pavel Povolni et. al., pavel.povolni@tuebingen.mpg.de

Summary:
This manuscript presents the development and validation of a new NMR field probe for measuring magnetic fields at low field strengths. The main technical innovation of this work is the use of SiC-nFETs in intentional avalanche mode, enabling high voltages and rapid current ramps for a non-adiabatic prepolarization switch off.

In the revision and rebuttal documents, the authors address our revision comments earnestly. The authors improved the structure of the manuscript, making significant improvements to the introduction section by removing extensive reference to the UN's SDGs, and instead addressing limitations of currently available (Hall-Effect sensors, NMR probes at high-field, etc.) field measurement systems.

Further, the author's make significant improvements to the presentation of data, including the inclusion of fitted spectra (Fig. 5, Fig. 9), confidence bands, and in-depth discussion of the statistical analysis and fitting run on data in figure captions.

However, significant limitations remain in the manuscript. Though the author's do address comments regarding data quality and reliability of measurements, we find that their response is insufficient to convincingly support their conclusions. For example, Figure 9, detailing data collected with the NMR field probe at 45 mT, includes many 'missing' spectra which are not adequately explained, and which call into question the reliability and repeatability of field measurements acquired at the upper end of the proposed dynamic range of the field probe. This is additionally reflected in Figure 8, which details RF excitation at 1 mT, and Figures 6 & 7, which describe the gradient offset analysis.

Given these unresolved issues, the current dataset does not convincingly demonstrate the performance claimed across the full dynamic range of the field probe. Additional data acquisition, or a substantial re-evaluation of the claims and supporting analysis, is necessary before the conclusions can be considered adequately supported.

Major Issues Remaining:

1. **Writing and Structure:** Though the authors removed reference to SDGs to instead present current approaches to measuring magnetic fields at low field

strengths, the motivation for their approach is still underdeveloped. The structure by which they break current measurement methods into HFMRI and LFMRI categories is confusing, and explicit limitations (i.e. dynamic range, cost, accuracy, precision) are not mentioned for existing approaches, which weakens the motivation for their novel approach.

2. **Data Quality and Convincingness of Conclusions:** Though the authors address our concerns in their rebuttal document, we find that their revisions were insufficient to assuage our concerns. The authors did a good job explaining why repeated measurements are difficult to achieve in their systems due to significant field drift, and in turn included confidence intervals and fits to demonstrate the ability of their probe to detect resonant peaks, which strengthens the presentation of their data. However, the quality of their data is unconvincing. Specifically, data presented in Figures 6, 7, 8, and 9 are noisy, inconsistent, and don't strongly follow fitted trends.

   Figure 6, while greatly improved by the inclusion of confidence intervals, still demonstrates weak adherence to linear and exponential trends without sufficient explanation. Figure 7, which details the gradient offset analysis, is generally confusing. The use of the NMR probe for measurement of gradient fields is not strongly motivated in the manuscript, and the authors choice to move the field probe for different field strength measurements is confusing, as the reliability of the system (i.e., does the probe correctly localize gradient offset across different field strengths?) isn't demonstrated.

   Figure 8, which investigates use of RF excitation at 1 mT to motivate the operating range of the scanner, ultimately includes very messy data, in which spectra cannot be extracted at many prepolarization field strengths. Further, the FWHM and Amplitude graphs to do not demonstrate convincing trends. While we appreciate that the authors include this data to represent NMR probe operation at the 'worse-case', the data is ultimately unconvincing and weakens their assertion that their probe can operate within the dynamic range from 1 mT – 45 mT. This is further reflected in Figure 9, which includes generally very noisy data. Though figure presentation is greatly improved, the data quality is still unconvincing, particularly when 'missing spectra' appear. A significant amount of this data has no detectable spectral peak, and other data sets are fit so austerely that its unconvincing. Ultimately, an expanded analysis which examines the performance of their probe at more intermediate fields would benefit the authors. The inclusion of data that is primarily acquired at the limits of the proposed dynamic range

should be accompanied by data on intermediate fields that may better demonstrate the SNR, reliability, and accuracy of their measurements.

Specific Comments:

1. **<u>Introduction:</u>**
    1.1. Don't need to include "consistent with various studies, including more recent publications", as this implies that the findings in 6 are outdated.
    1.2. Be careful with the idea that low-field scanners are "significantly less expensive", as considerable attention has been paid to the cost of commercially available low-field systems. Consider changing to "can be significantly less expensive".
    1.3. The wording here is awkward: "require minimal infrastructure because, e.g., cryogenic cooling is not necessary". I understand that you are trying to provide an example of why operational cost is reduced, but the use of e.g. is clunky without saving that much space.
    1.4. Change to "recent developments have demonstrated the feasibility of in-vivo imaging with permanent-magnet-based MRI systems…."
    1.5. The discussion of permanent magnet-based designs vs. electromagnets seems out of place. As this paper focuses on implementation of the field probe, the discussion of trade-offs between electromagnet and permanent magnet systems seems unnecessary. Further, perhaps the most significant limitation of electromagnets has to do with system power and cooling requirements, which are more problematic in low-resource settings.
    1.6. You state "a fundamental problem for the entire LFMRI research is the lack of highly precise magnetic field sensors:…", but following the colon you go onto describe why sensors are needed in general, not the lack of highly precise sensors.
    1.7. The comparison between HFMRI and LFMRI field mapping seems like an unnecessary distinction. Further, reference 31 is using a self-built array of NMR sensors for a LFMRI system, not a HFMRI system. Additionally, the idea that access to commercial systems is limited due to "enormous high cost" that "may be more than the cost of LFMRI alone" is not well supported/referenced. Additionally, you did not address the limitations of the self-built systems (30,31) referenced, so this conclusion (inaccessible due to cost) is not applicable to all HFMRI field measurement systems.
    1.8. In general, it seems like the most apt comparison for your system are commercially available 3-axis Hall Effect gaussmeters. I would limit the scope of your discussion to these devices to improve clarity.
    1.9. Overall, the claim that "a fundamental problem for the entire LFMRI research is the lack of highly precise magnetic field sensors" is a substantial claim that isn't

well supported or motivated by the additional background you have included. Though you state that NMR probes are the most accurate, you haven't necessarily motivated that Hall effect sensors are not "highly precise" or that their precision is completely unsuitable for use in measuring LFMRI magnets, and the idea that other systems are extremely expensive isn't well motivated either.

1.10. You state that a large challenge is the large dynamic range needed for measurement of the field during construction, but you don't compare the dynamic range of existing field measurement sensors. For example, Hall effect gaussmeters often have very high dynamic range, so a priori knowledge of the field isn't required for these sensors either, and they are commonly used during construction to catch errors (i.e., mapping of individual rings during construction).

1.11. Ppm is typically used in the context of describing field variation, not relative/remaining field strength. The idea that only 280 ppm of the signal remains at 50 mT is non-standard and confusing.

1.12. Good inclusion of reference to geophysical applications and the specific innovation of your sensor (3x faster ramp).

2. **<u>Results:</u>**

2.1. Non-Adiabatic Switch Off the Pre-Polarization Field<u>:</u>

2.1.1. Again, the inclusion of Eq. 2.1, though you state that a more detailed analysis is provided in the supplementary information, is confusing and unnecessary. This equation does not convincingly and clearly demonstrate the point that signal (U) is maximized with a large Bp and a short ramp time. Perhaps a different equation, or a more qualitative discussion, can better highlight the need for faster ramp times to maintain the NMR signal, and thus motivate your use of the SiC-nFET in intentional avalanche breakdown.

2.1.2. Nice, clear discussion of the novelty of using the SiC-nFET in intentional avalanche break down. Also, nice mention of the aging tests.

2.1.3. I would still like additional clarification about the operating range of this sensor. Under 5 mT, you state that the adiabatic condition is significantly violated for tested Bp under 70 mT. However, most low-field systems used in-vivo are above 5 mT, closer to 50 mT and above. Although you state that a weak transverse magnetization remains visible in simulation at 50 mT if the "sensor concept is designed accordingly", I don't find this particularly convincing when trying to demonstrate the feasibility/appropriateness of this NMR sensor over a traditional hall-effect sensor at low-field. In your rebuttal, you mention that you have tested the systems extremes, but again, an

operating point above 50 mT isn't really an extreme, and is rather the norm, for most in-vivo low-field imaging systems.

2.2. Design of the sample, polarization coil, and receive coil for maximum sensitivity:

2.2.1. Although you have removed the sentence "on the same order of magnitude as a voxel in LFMRI", the volume of the sample (1.2-1.5 mL) still seems very large and inherently limiting for the spatial resolution of this sensor. If you want high spatial resolution in your field map (which you generally do), then a volume of 1.5 ml represents a 15x15x15 mm voxel, which seems too large. Please additionally clarify or justify this.

2.2.2. You still use the term "thermal polarization"

2.2.3. I would still like a more explicit explanation of the different operating modes and their use cases for this sensor. The novelty of this sensor is the rapid NA switch-off facilitated by the intentional avalanche breakdown of the SiC-nFET, but the NA switch-off is only advantageous up to 26 mT. Beyond that, conventional RF excitation generates higher SNR, and you need to use a different Rx coil set up. Although you mention in your rebuttal that the receive coil still operates well +/- 24% B0 variation, this doesn't really address the fact that the proposed sensor only seems the most advantageous for ULFMRI, which isn't particularly common for in-vivo imaging in low-resource settings.

2.3. NMR Measurements in Ultra low field for B0< 5 mT:

2.3.1. Figure 6: Even though the authors responded and clarified why they have chosen to perform only a single measurement due to the drift in the main B0 field, without averages the fits look relatively unconvincing still. Its hard to appreciate the stability of the system from this data.

2.3.2. I am still confused by this analysis. The clarification from the author in the rebuttal helped to demonstrate the purpose of the experiment (to determine the positional offsets of the gradients relative to each other), but I don't think this is well communicated in the revised manuscript itself. Further, I am confused about the evaluation over 3 different field strengths. Did you deliberately move the probe each time (1,3,5 mT), and for what purpose? It would be useful to also explicitly NOT move the probe and calculate/fit the offsets at different strengths to show the accuracy of the sensor across this range of field strengths.

2.4. Hybrid measurement with RF excitation in Ultra-low field:

2.4.1. Figure 8: I appreciate what the author's included in their rebuttal about wanting to show the acceptable performance of the hybrid excitation even at 1 mT B0. However, I am still confused by the lack of trend in (b) and (c). If the amplitude, for example, doesn't increase monotonically with increasing

Bp, then is this performance suitable? Ultimately, I am confused as to why the authors are demonstrating the adiabatic hybrid performance at 1 mT when the performance of the NA switch off is far superior.

2.5. NMR Measurements in low-field at B0 = 45 mT:

2.5.1. *"Due to the significant frequency drift, averaging the spectra is not recommended as it broadens the measured peak and consequently reduce SNR. Moreover, averaging is not necessary, as SNR is sufficient for peak detection and a robust spectral fit in a single measurement already."*

1) Please clarify what is meant by "frequency drift" in this context. Figure 9 includes an intentionally applied frequency offset; is this offset intended to represent the drift referred to here?

2) The statement that the SNR is sufficient for robust fitting from a single measurement does not appear to be supported by the data shown in Figure 9, where many spectra appear to have low SNR and poorly distinguishable peaks.

2.5.2. Figure 9:

1) Without averaging, the data at 45 mT remain unconvincing. In many instances, the peak corresponding to the B0 field appears to be at or near the noise floor. Furthermore, fitted spectra are not shown for many measurements. The authors suggest that the missing spectra could be due to switching of the prepolarization generator; however, given the large number of measurements in which no peak is detectable, this raises concerns regarding the reliability and repeatability of the system at the upper end of its proposed operating range.
2) Several displayed fits appear difficult to justify based on the presented data (for example, S4 of repetition 2). Please clarify the fitting procedure, including any fitting constraints and criteria used to identify valid peaks.
3) The tolerance band of the Hall sensor also appears quite large. Please provide additional explanation regarding the origin of this tolerance band and how it should be interpreted relative to the NMR-based measurements.
4) Overall, the 45 mT results, which the authors describe as representing the upper limit of operation, do not convincingly demonstrate reliable probe performance at this field strength. Given that the manuscript motivates the work in part through applications in low-field MRI, where operating fields are often on the order of 45 mT or higher, this limitation should either be addressed with additional evidence or more clearly reflected in the claims.

2.5.3. Figure 9 caption states: "The vertical lines correspond to the measured value from the embedded Hall sensor (with the tolerances of the used sensor added), to the mean Larmor frequency of the three measurements,

and to the expected Larmor frequency due to the additional offset coil (see Figure 4e)." However, three distinct vertical lines are not visible in all subplots. In addition, the legend indicates dotted lines, whereas the caption refers to vertical lines without clearly identifying their styles. It is also unclear whether the legend applies to both panels or only to Figure 9b, where dotted lines are present.

3. **Discussion:**

3.1. While the authors have improved the discussion, I remain unconvinced regarding the utility of the proposed system across the full claimed operating range of 1–50 mT. At the low end of this range (1 mT), the hybrid approach does not appear particularly robust, while at the high end (45–50 mT), the performance of the NA prepolarization approach appears limited (see comments regarding Figure 9). Ultimately, the challenges observed at the limits of the proposed dynamic range should be discussed in the context of existing field measurement technologies, such as Hall-effect sensors, rather than solely in comparison with other NMR-based sensors that employ adiabatic switch-off at higher field strengths.
