# Supplementary material for: An integrated NMR field probe using rapid non-adiabatic field switch-off for (ultra) low field MRI
Source: Commun Eng. 2026 Oct 28;5:183. doi: 10.1038/s44172-026-00815-0 (PMC13720019; doi:10.1038/s44172-026-00815-0)
Supplement: Supplementary file 2 [file 44172_2026_815_MOESM2_ESM.pdf]

An Integrated NMR Field Probe Using Rapid Non-Adiabatic Field Switch-Off for (Ultra) Low Field MRI

Pavel Povolni[1*°], Dominique Goerner[1,2°], Praveen Iyyappan Valsala[1,3], Lukas Gebert[2], Georgiy Solomakha[1], Nicolas Kempf[1], Felix Glang[1,4], Judith Samlow[1,5], Ruben Schnitzler[2], Ingmar Kallfass[2], Kai Buckenmaier[1*], Klaus Scheffler[1,3]

[1] High-Field Magnetic Resonance Center, Max Planck Institute for Biological Cybernetics, 72076 Tübingen, Germany
[2] Institute of Robust Power Semiconductor Systems ILH, University of Stuttgart, 70569 Stuttgart, Germany
[3] Department for Biomedical Magnetic Resonance, University of Tübingen, 72076 Tübingen, Germany
[4] Institute of Biomedical Imaging, Graz University of Technology, 8010 Graz, Austria
[5] Hospital Diospi Suyana, 03100 Curahuasi, Apurímac, Peru

° these authors contributed equally

* Corresponding authors: pavel.povolni@tuebingen.mpg.de, kai.buckenmaier@tuebingen.mpg.de

---

Content

1 Calculation of transverse magnetization after switch-off process 2
2 Calculation of the signal after switch-off 5
3 Analysis of the Adiabatic Condition 6
4 Simulated Noise for SNR Estimation 8
5 Data Processing of Measured Data (Figure 5, 6, 7, 8) 9
6 Calculation of the normalized slope (Figure 2e) 11
7 Analysis of Gradient Measurements 12
8 Further Measured Data in Hybrid Mode (Figure 7) 14
9 Long-term investigation of Aging of the Silicon Carbide Field-Effect Transistors (SiC-nFET) 15
10 Analysis of measurement variability at B0 = 45 mT 17
11 Investigation of Power Source Instability for the Ultra Low Field magnet 24
12 Accuracy of frequency measurement with the NMR field probe 25
13 Ultra Low Field Magnet 26
14 References 28

1 Calculation of transverse magnetization after switch-off process

The sample is pre-polarized by a strong polarization field B_p, which is then abruptly switched off. Because of the circuit design, the switch-off ramp exhibits a quasi-linear characteristic. In order to proceed with the design of the receive circuit, it is necessary to estimate the remaining transverse magnetization after the successful switch-off. Simultaneously, it is possible to adjust the pre-polarization circuit and coil such that a good trade-off between the B_p field strength, the switch-off duration t_p and the available, measurable magnetization is made. The calculation of the available transverse magnetization is derived in the following sections.

The time varying total spatial magnetic fields are:

$$B_x(t) = \begin{cases} B_p\left(1 - {}^{t}/_{t_p}\right) & \middle|\, 0 \le t \le t_p \\ 0 & \middle|\, t_p \le t \end{cases} \tag{S1.1}$$

$$B_y = 0\ \forall t \tag{S1.2}$$

$$B_z = B_0\ \forall t \tag{S1.3}$$

This results in the differential equation according to Felix Bloch [1]:

$$\begin{pmatrix} \dot{M}_x(\mathrm{t}) \\ \dot{M}_y(t) \\ \dot{M}_z(t) \end{pmatrix} = \gamma \begin{pmatrix} 0 & B_z & 0 \\ -B_z & 0 & B_x(t) \\ 0 & -B_x(t) & 0 \end{pmatrix} \begin{pmatrix} M_x(t) \\ M_y(t) \\ M_z(t) \end{pmatrix} \tag{S1.4}$$

Due to the short switch-off time, longitudinal and transverse relaxations are neglected.

This results in a double accelerated coordinate system for the macroscopic spin vector: On the one hand, the effective magnetic field to which the spins are oriented rotates around the y-axis of the laboratory system with time-varying amplitude B_{eff}, angle φ, and angular velocity ω_{eff}:

$$B_{eff}(\mathrm{t}) = \sqrt{B_x(t)^2 + {B_0}^2} \tag{S1.5}$$

$$\varphi(\mathrm{t}) = \mathrm{atan}\left({}^{B_x(t)}/_{B_0}\right) \tag{S1.6}$$

$$\omega_{eff} = \frac{d\varphi(t)}{dt} = \frac{-B_0 B_p t_p}{{B_p}^2 t^2 - 2{B_p}^2 t_p t + \left({B_0}^2 + {B_p}^2\right){t_p}^2} \tag{S1.7}$$

Additionally, the magnetization precesses around the effective magnetic field B_{eff} (Larmor precession). This precession slows down due to the time-varying magnetic field strength, resulting in the following relationship between precession frequency ω_L and magnetic field strength:

$$\omega_L(t) = \gamma_{1H} B_{eff}(\mathrm{t}) \tag{S1.8}$$

An analytical solution to the differential equation S1.4 cannot be found. One potential approach involves the use of the Magnus expansion, though it should be noted that convergence of the series is not guaranteed (our approximation up to the 5th order was not sufficiently accurate). Therefore, the necessity arises for a numerical solution to the differential equation. The process of solving the differential equation can be time-consuming, particularly when one attempts to sweep B_0, B_p, and t_p in three dimensions simultaneously. In the present study, a high-performance computer node (512 GB RAM, 2x64-core AMD EPYC 7452 2.35 GHz) was utilized for a duration of several hours to complete the calculation of the magnetization trajectory. However, for the design of the Rx circuit, only the remaining transverse magnetization after the switched off pre-polarization at $t = t_p$ needs to be

known. Simplified approximations can therefore be employed to rapidly test a large number of designs, thus facilitating the process of design iteration. Based on numerical solutions, in the range (which should be interesting and practical for (ultra) low field MRI):

$$1mT \ \leq B_0 \leq 60mT$$
$$5mT \ \leq B_p \leq 100\ mT$$
$$200\ ns \ \leq t_p \leq 4800\ ns$$

a quadratic approximation was calculated.
The basis is the following dependence of the transverse magnetization $M_\perp$remaining at the Rx-start:

$$M_\perp(t_p) = m_{rel}(B_0, B_p, t_p) M_{\perp 0}(B_0, B_p) \tag{S1.9}$$

With transverse magnetization $M_{\perp 0}$ at time $t = 0s$ (steady state) (equation 6.10 [2]):

$$M_{\perp 0}(B_0, B_p) = \rho_0 \frac{\gamma^2 \hbar^2}{4kT} B_{eff}(t = 0) \tag{S1.10}$$

with the spin density per unit volume $\rho_0 = 6.68 \cdot 10^{28}$ for water (proton), the gyromagnetic ratio $\gamma = 2.675 \cdot 10^8 s^{-1} T^{-1}$ for water (proton), the reduced Planck constant $\hbar$, the temperature T in Kelvin and the Boltzmann constant k.
The following empirical formula (B_0 in mT, B_p in mT, t_p in µs) is employed to calculate the magnetization ratio:

$$\begin{aligned} m_{rel}(B_0, B_p, t_p) = p_1 {B_0}^2 + p_2 {B_p}^2 + p_3 {t_p}^2 \\ + p_4 B_0 B_p + p_5 B_0 t_p + p_6 B_p t_p \\ + p_7 B_0 + p_8 B_p + p_9 t_p + p_{10} \end{aligned} \tag{S1.11}$$

where the averaged time period of the Larmor precession is used to separate 3 individual ranges for higher accuracy:

$$t_L = \left(\gamma \sqrt{(0.5 B_p)^2 + {B_0}^2} \right)^{-1} \tag{S1.12}$$

with the gyromagnetic ratio $\gamma = 42.577\ MHzT^{-1}$ for water (proton),
The calculated parameters are:

	Unit	$t_L \leq 0.5t_p$	$0.5 < t_L \leq 1.5t_p$	else
p1	[1/mT²]	+ 4.41e-04	+ 1.27e-04	+ 1.12e-04
p2	[1/mT²]	- 3.59e-06	- 1.83e-05	+ 3.97e-06
p3	[1/µs²]	+ 0.0134	+ 0.0141	- 9.02e-03
p4	[1/mT²]	+ 7.20e-05	+ 5.79e-05	- 4.28e-06
p5	[1/µs/mT]	- 9.56e-04	- 1.38e-02	+ 0.0464
p6	[1/µs/mT]	- 3.28e-04	- 2.15e-04	+ 1.43e-03
p7	[1/mT]	+ 0.0397	- 0.0103	- 8.57e-03
p8	[1/mT]	- 5.51e-03	+ 7.01e-03	+ 9.70e-04
p9	[1/µs]	+ 0.137	- 0.0971	- 0.0548
p10	[1]	- 0.990	+ 0.612	+ 1.02

The mean discrepancy between the numerical solution and the approximation is 5%, with deviations of up to 30% possible in individual extreme cases (in the case of extremely unusual combinations such as long t_P with weak B_p). If needed, this calculated level of accuracy should be adequate for the design of new Rx electronics or Rx coils. The comparison of both calculations is demonstrated in Figure S1.

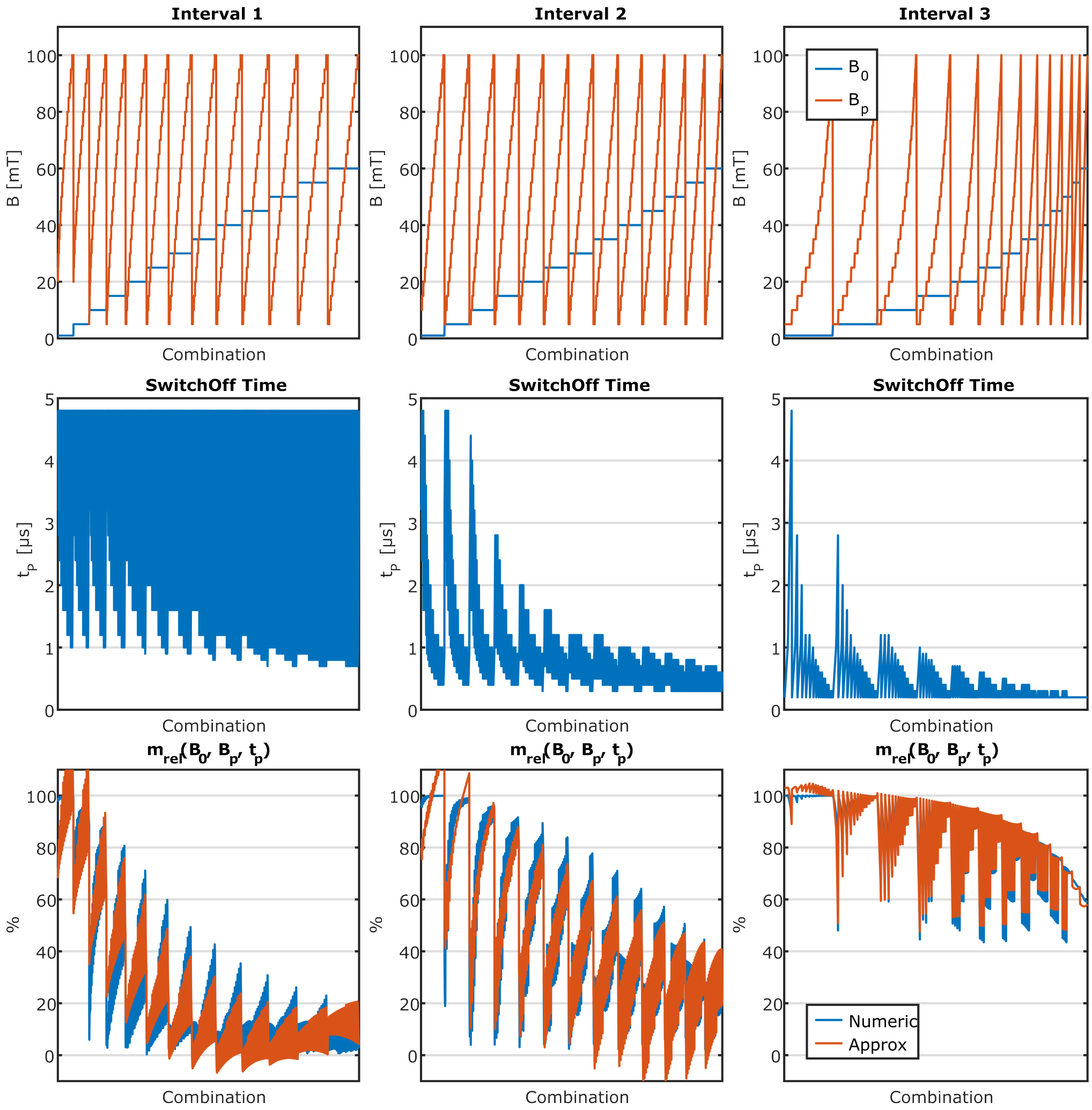

Figure S1: Comparison of the approximated and numerically calculated solutions for m_{rel} depending on the various combinations of B_0, B_p, and t_p.

2 Calculation of the signal after switch-off

Subsequent to the described switch-off process, the values of B_x and B_y are 0 and B_Z=B_0. Consequently, the measurable transverse magnetization will rotate around the z-axis of the laboratory system. The Rx coil is oriented along the y-axis. To calculate the induced voltage the reciprocity principle can be used [3]. For the purposes of simplicity, the following relationship is assumed for the transverse magnetization $M_\perp$ for $t > t_p$ (with parameterization $t' = t - t_p$ and a start phase ϑ):

$$M_\perp(t') = M_\perp(t_p)\exp\left(-{t'}/{T_2^*}\right)\sin(\omega_L(B_0)t' + \vartheta) \quad \text{(S2.1)}$$

where transverse relaxation was replaced by T_2* due to the expected inhomogeneities coming from the switching field and its disturbances. This results in the induced voltage in a receiving coil being [3], [4]:

$$U(t') = \int_V \omega_L B_\perp(dV) M_\perp(t')\, dV \quad \text{(S2.2)}$$

where the spatial sensitivity of the Rx coil is considered in the parameter $B_\perp(dV)$.
In the ideal experiment, $B_x(t = 0) \gg B_z$ and $t_p \rightarrow 0$ applies (extremely fast and strong switchoff) so that m_{rel} becomes $m_{rel} \approx 1$. Then the remaining transverse magnetization after the switchoff is:

$$M_\perp(t_p) \approx M_{\perp 0}(B_0, B_p) = \rho_0 \frac{\gamma^2\hbar^2}{4kT} B_p \quad \text{(S2.3)}$$

And thus, the induced voltage becomes (without relaxation processes):

$$U(t') = \gamma B_0 M_\perp(t_p)\exp\left(-{t'}/{T_2^*}\right)\sin(\omega_L(B_0)t' + \vartheta)\int_V B_\perp(dV)\, dV \sim B_0 B_p \quad \text{(S2.4)}$$

3 Analysis of the Adiabatic Condition

If the pre-polarization field $B_p(t)$ is switched off slowly, the sample magnetization realigns with B_0 after the finished switch-off. The following formula can be used to estimate this adiabatic case [5]:

$$\gamma\left|\vec{B}(t)\right| \gg \left|\frac{d}{dt}\angle\left(\vec{B}(t),\overrightarrow{B_0}\right)\right| \mid \forall t \tag{S3.1}$$

with $\vec{B}(t) = \overrightarrow{B_p}(t) + \overrightarrow{B_0}$ and the γ gyromagnetic ratio. Note that the operator $\angle$ denotes the angle between $\vec{B}$ and $\overrightarrow{B_0}$.

Based on this equation, a factor R can be defined to quantify the adiabaticity:

$$R = \min_{\forall t} \frac{\gamma\left|\vec{B}(t)\right|}{\left|\frac{d}{dt}\angle\left(\vec{B}(t),\overrightarrow{B_0}\right)\right|} \gg 1 \tag{S3.2}$$

The left-hand side of equation S3.1 describes the time-varying Larmor precession of the sample. The right-hand side describes the angular velocity at which the time-varying magnetic field $B(t)$ rotates around B_0.

In our case, B_p is aligned along the x-axis and B_0 along the z-axis. Therefore, the angle on the right-hand side of equation S3.1 is:

$$\angle\left(\vec{B}(t),\overrightarrow{B_0}\right) = \operatorname{atan}\frac{B_p(t)}{B_0} \tag{S3.3}$$

For simplicity, the pre-polarization field is switched off linearly (see equation S1.1). Figure S2 shows equation S3.1 over time for different magnetic field of $1mT \leq B_0 \leq 50mT$. It is assumed that the initial $B_p(t=0) = 70mT$ is switched off in a timespan $t_p = 1\mu s$. Figure S2a shows the time domain for three exemplary B_0. For $B_0 = 1mT$ and $B_0 = 10mT$, the adiabatic condition is clearly violated at $t = 909ns$ and $t = 864ns$, respectively. These switchoff processes are therefore clearly non-adiabatic resulting in a transverse magnetization after the finished switchoff. For $B_0 = 30mT$, the left side of equation S3.1 is at least a factor of $R = 3.4$ greater than the right side at any given time. Figure S2b shows the intersection point for different B_0 values. From $B_0 \geq 16$ onwards, there is no longer an intersection point between the two sides of the equation S3.1. For these B_0, Figure S2c shows the ratio R of both equation sides. At $B_0 = 45mT$ a ratio of $R = 7.7$ is calculated. However, since an successful NMR experiment was possible at this field strength, it can be assumed that this factor is still insufficient to comply with $R \gg 1$ (see equation S3.2.).

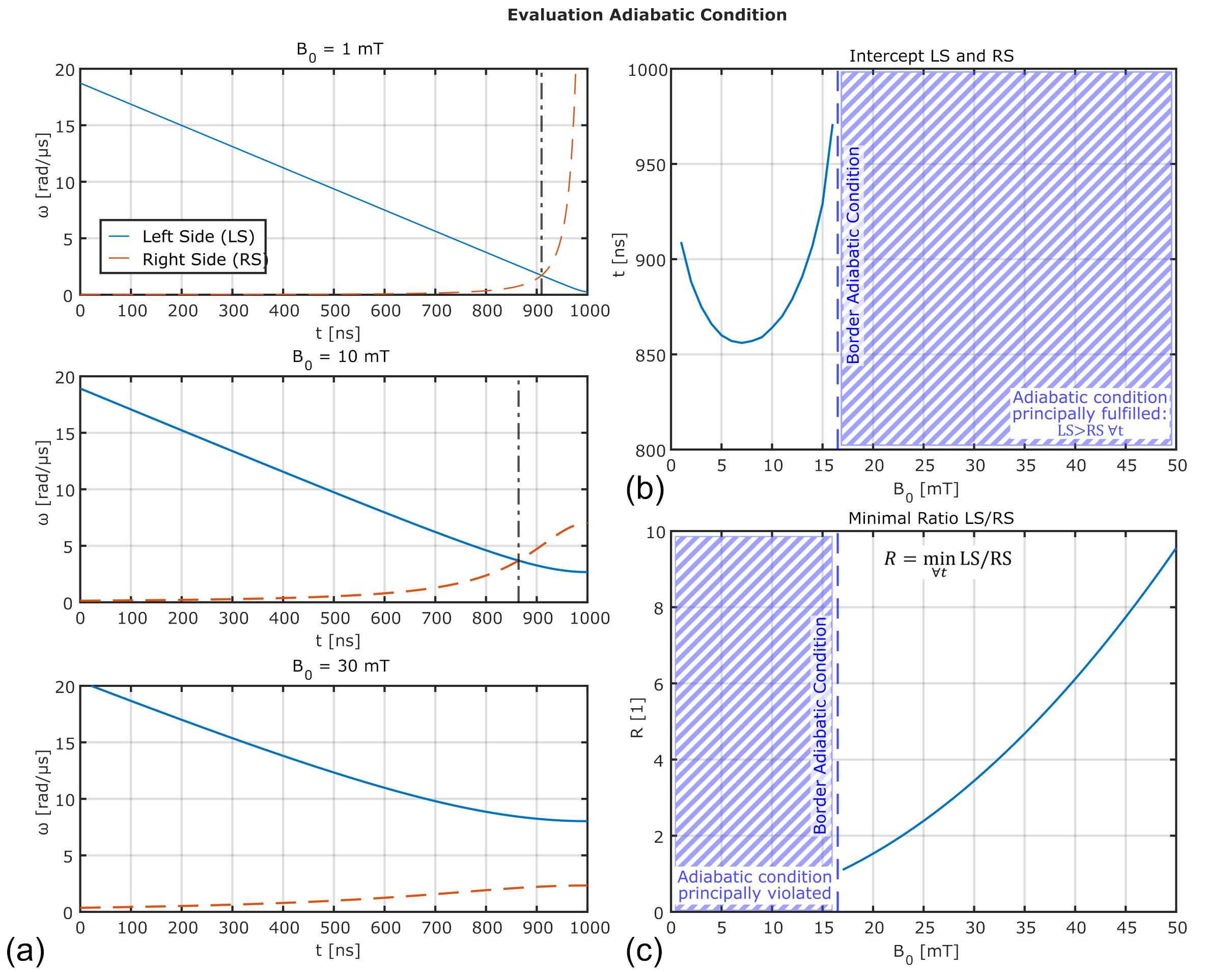

$$\text{Adiabatic Condition}\quad \underbrace{\gamma|\vec{B}(t)|}_{\substack{LS \\ (Left\ Side)}} \gg \underbrace{\left|\frac{d}{dt}\angle(\vec{B}(t),\vec{B_0})\right|}_{\substack{RS \\ (Right\ Side)}} \mid \forall t$$

Figure S2: Further details on the adiabatic condition according to Tannús et al [5]. (a) Overview of the time course of the left and right sides of the known formula for the adiabatic condition, calculated for the magnetic fields $B_0 = 1mT$, $B_0 = 10mT$, and $B_0 = 30mT$. (b) Intersection between the left and right sides of the formula for different B_0. From $B_0 > 16mT$, the adiabatic condition is principally fulfilled. (c) Calculation of the ratio between the left side and the right side in the adiabatic case.

4 Simulated Noise for SNR Estimation

As the only noise source, the thermal noise of the ohmic resistance R of the Rx-coil was used in simulation. Therefore, the frequency dependent Skin- and Proximity effect were considered, which significantly increase the ohmic DC-resistance R_{DC} depending on the chosen coil geometry and measured frequency of the signal f.

For the skin-effect a frequency dependent factor $\Delta_{sk}(f) > 1$ [6] and for the Proximity-effect a second frequency dependent factor $\Delta_{px}(f) > 1$ [7] is calculated, which results in an effective ohmic resistance of $R(f) = \Delta_{sk}(f)\Delta_{px}(f)R_{DC}$.

The thermal noise U_{noise} of this resistance is calculated using the Johnson-Nyquist formula:

$$U_{noise} = \sqrt{4\, k_B\, T\, R(f)\, BW} \tag{S4.1}$$

with the Boltzmann constant k_B, the temperature $T = 300K$, the ohmic resistance $R(f)$ of the Rx-coil and the bandwidth BW of the Rx electronic.

For $B_0 \leq 5mT$ the sampling frequency was set to $f_S = 2MSps$ and therefore the sampling bandwidth is $BW = f_s/2 = 1MHz$, resp. for $B_0 = 45mT$ the sampling frequency was set to $f_S = 10MSps$ and therefore the sampling bandwidth is $BW = f_s/2 = 5MHz$. The cut-off-frequency of the anti-aliasing-filter was held constant at $f_{-3dB} = 3MHz$, which therefore results in aliasing for $B_0 \leq 5mT$ and increased effective bandwidth to $BW = 1.95MHz$ and therefore increased noise. For $B_0 = 45mT$ the anti-aliasing-filter limits the effective bandwidth to $BW \approx 1.006 \cdot 3MHz$ (since a 6th order Butterworth filter is used).

The SNR was then calculated using the effective value of the simulated signal $u_{sig} = \hat{u}_{sig}/\sqrt{2}$:

$$SNR = 20\, log_{10}\, u_{sig}/\, U_{noise} \tag{S4.2}$$

5 Data Processing of Measured Data (Figure 5, 6, 7, 8)

The following section illustrates data processing using a measurement at $B_0 = 3mT$ (see the spectrum in the center of Figure 5) as an example. The corresponding signals are shown in Figure S3.

1) Sampling of the measurement data: Depending on the required accuracy, multiple measurements can be performed sequentially and then averaged. Overshoots may still be present at the beginning of the sampled data, caused by the slight inductive coupling between the Rx coil and the pre-polarization coil and the rapid current ramps.
2) Signal Trimming: The first samples are removed. Alternatively, sampling can be delayed until the fluctuations have decayed.
3) Bandpass filtering: The filter cutoffs correspond to the measured magnetic field and the maximum tolerances of the embedded Hall sensor.
4) Apodization Filtering: (see the 'Methods' section in the main manuscript for the equation).
5) Fourier Transform: For standalone sensor operation, it is sufficient to evaluate the absolute spectrum and detect the maximum. If necessary, multiple spectra can be averaged (see step 8 here).

From this step onwards, the data is processed in more detail for the analysis and characterization of the build hardware in this publication. However, this not necessary for stand-alone sensor operation.

6) Zero-order Phase Correction: Here, the complex spectrum S_0 is multiplied by a static phase ρ_0 so that absorption and dispersion are clearly separated.

The new spectrum S_1 is thus given by:

$$S_1 = S_0 \exp\,(i\rho_0) \tag{S5.1}$$

This step can be performed automatically. This correction is applied to the spectra shown in the manuscript. Higher orders are not corrected for the illustration, but used for the following fit for robustness of the algorithm.

7) First-order Phase Correction: The signal S_1 is multiplied by a first-order frequency-dependent phase $\rho_1(f)$:

$$S_2 = S_1 \exp\big(i\rho_1(f')\big) \tag{S5.2}$$

with the frequency axis related to the central frequency f_{NMR} (maximum of the absolute NMR spectra):

$$f' = f - f_{NMR} \tag{S5.3}$$

8) Averaging: Could be also performed earlier.
9) Lorentzian Fit: Based on the real part of the spectrum, an ideal NMR peak is fitted as a Lorentzian curve S_{fit} with parameters $a_1, \dots a_5 \in \mathbb{R}$:

$$S_{fit}(f) = \frac{a_1}{\pi} \frac{a_3}{a_3^2 + (f - a_2)^2} + a_4 \tag{S5.4}$$

To achieve a more robust result, the fit was performed in the integrated form[8]:

$$S_{CD}(f) = \int S_{fit}(f) df = \frac{a_1}{\pi} \operatorname{atan}\left(\frac{f - a_2}{a_3}\right) + a_4(f - a_2) + a_5 \tag{S5.5}$$

The least square algorithm was used to optimize the parameters $a_1, \dots a_5$.

Since, based on our experience, this fit performs extremely well in its integral form and converges very quickly, we assume that it would also be possible to be integrated on a powerful embedded SoC chip, or ARM based CPU for a standalone sensor operation.

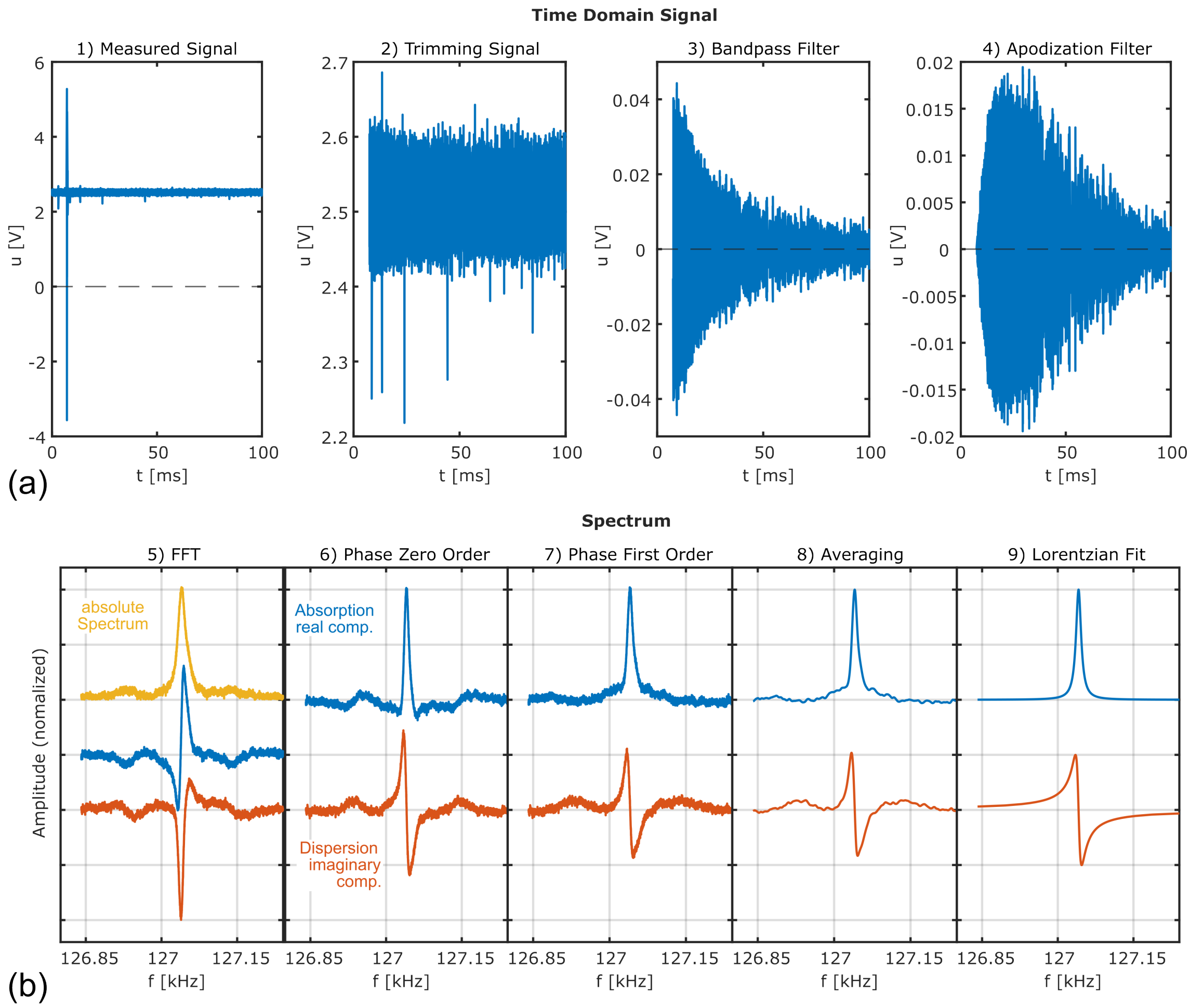

Figure S3: Illustration of the data processing of the measured signal. (a) Measured time signal and time-domain filtering. (b) Spectrum analysis, as well as further corrections and fitting of the signal. For clarity, the amplitudes have been normalized and shifted relative to one another on the vertical axis.

6 Calculation of the normalized slope (Figure 2e)

We compared the evolution of the maximum amplitude in the NMR spectrum û, or the SNR, across varying B_p field strengths ($t_p = 6s$ fixed). The measured $\hat{u}(B_p)$, resp. $SNR(B_p)$, is shown in Figure 6a. The simulated values are shown in Figure 2d. Due to slight variations in the Rx coil geometries, a direct qualitative comparison between the two measurements is not feasible (the signal amplitude is significantly smaller in the measurement due to less windings).

Because of the random winding pattern of the real Rx coil, modeling it accurately in simulation is not possible.

To ensure a quantitative comparison, the values were normalized to the maximum value before a linear fit (both for the measured and simulated data):

$$\hat{u}_{norm}(B_p) = \hat{u}(B_p) / \max_{\forall B_p} \hat{u}(B_p) \tag{S6.1}$$

$$SNR_{norm}(B_p) = SNR(B_p) / \max_{\forall B_p} SNR(B_p) \tag{S6.2}$$

Note that SNR is used here in its absolute, linear form (**not** logarithmic in dB). These normalized data are then linearly fitted (least-square fit method), resulting in the following relationship:

$$\tilde{u}(B_p) = a_{\hat{u}} B_p + OS_{\hat{u}} \tag{S6.3}$$

$$\widetilde{SNR}(B_p) = a_{SNR} B_p + OS_{SNR} \tag{S6.4}$$

with the slopes $a_{\hat{u}}$, resp. a_{SNR}, (unit 1/mT) and a dimensionless offset $OS_{\hat{u}}$, resp. OS_{SNR}. Both slopes are shown in Figure 2e of the manuscript.

The tolerance of the fitted slope for the evaluated datasets (measured and simulated) is calculated from the calculated residuals. For the simulated SNR this becomes very small with error bars almost not visible in the scaling of Figure 2e.

7 Analysis of Gradient Measurements

The integrated magnetic field sensor is sufficiently sensitive to detect the weak magnetic field variations generated by the installed gradient coils. For the characterization shown in Figure S6, the sensor was positioned as close as possible to the isocenter of the magnet with an unknown positioning tolerance between the individual field setups.

The frontend remained stationary throughout each measurement series and the X-, Y-, and Z-gradients were swept sequentially. Although the sensor position was not varied by known spatial offsets, it is enough to determine the relative sensor position to the center of gradients (e.g. to analyze the position of the sensor in an application like a field camera, where the sensor position relative to the MRI scanner is needed).

The gradient coils were driven individually with an adjustable DC current I_G ($-250mA \leq I_G \leq 250mA$). Each gradient was activated individually (only one switched-on at a time). The simulated efficiency of the gradient system ($\tilde{G}_X = \tilde{G}_Y = 1594nT/m/A$, $\tilde{G}_Z = 4833nT/m/A$) can be used to calculate the applied gradient $G_{X,Y,Z} = \tilde{G}_{X,Y,Z} I_G$ (shown as the x-axis of the plot) by multiplying it by the measured current I_G.

The frequency drift $\Delta f(G_{X,Y,Z})$ relative to the zero point $f_0 = f_{X,Y,Z}(G_{X,Y,Z} = 0nT/cm) = \gamma B_0$ (all gradients switched off) was calculated from the acquired NMR spectra:

$$\Delta f_{X,Y,Z}(G_{X,Y,Z}) = f_{X,Y,Z}(G_{X,Y,Z}) - f_0 \tag{S7.1}$$

This frequency shift was linearly fitted:

$$\widetilde{\Delta f}_{X,Y,Z}(G_{X,Y,Z}) = aG_{X,Y,Z} + OS \tag{S7.2}$$

with the slope *a* and an offset *OS*.

This linear fit can be used to determine the distance $d_{X,Y,Z}$ to the isocenter of the individual gradient:

$$d_{X,Y,Z} = \frac{\widetilde{\Delta f}_{X,Y,Z}(G_{X,Y,Z})}{\gamma G_{X,Y,Z}} \tag{S7.3}$$

The calculated results are also shown in Figure S6.

The resulting frequency shifts are presented in Figure S6a. The observed frequency offsets indicate a significant positional deviation, most likely caused by imperfect gradient alignment. For each measurement point, the gradients were first switched off to determine the intrinsic magnetic field before the selected gradient current was applied. The unexpected maximum observed at $B_0 = 5mT$ with all gradients switched off is likely caused by temporal magnetic field drift (see SI chapter 10). Figure S6b shows the corresponding changes in the full width at half maximum (FWHM) as a function of the applied gradient at $B_0 = 5mT$, demonstrating that the sensor is sufficiently sensitive to enable first-order shimming.

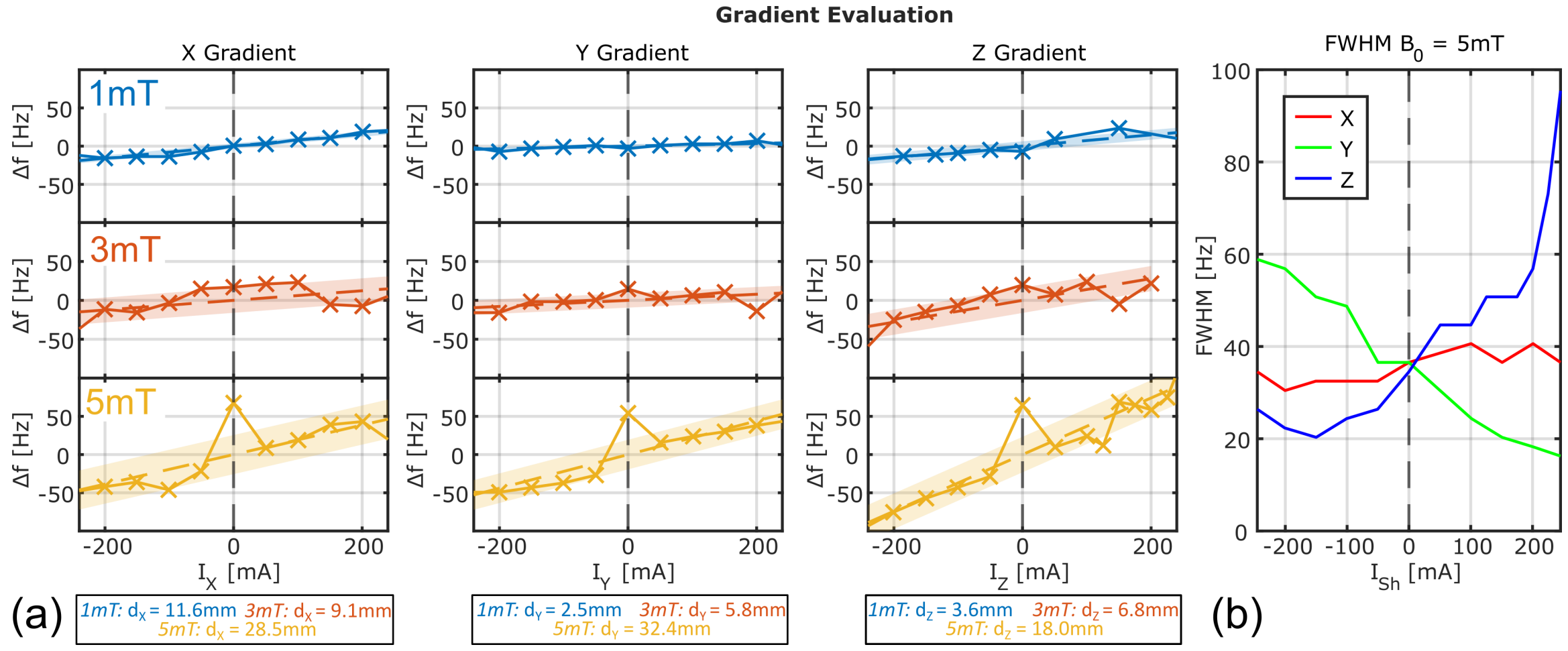

Figure S4: Investigation of the performance of the ULFMRI gradients. **(a)** Measured frequency shift with an varied X/Y/Z gradient in ULF (in all cases B_p=61mT, t_p=6s), with slight differences in the positioning accuracy of the frontend for the different B_0. Only one measurement was performed (no averages, so simulating a fast acquiring mode for dynamic mapping). The gradients were set in a range $\Delta I_{X,Y,Z}$=±250mA (11 equal steps). The acquired frequency offset was linearly fitted (the shown uncertainty represents the standard deviation of the fit). The calculated position of the frontend for the different B_0 ($d_{X,Y,Z}$) are shown in the textboxes (see SI chapter 7 for calculation details). **(b)** Measured FWHM for experiments at B_0=5mT for swept gradient currents. Only one gradient was switched on at a time. One spectrum was acquired (no averaging). The strongest influence on the linewidth (indicating inhomogeneity inside the sample) is caused by the Y and Z gradients (which indicates stronger field inhomogeneity in these directions).

8 Further Measured Data in Hybrid Mode (Figure 7)

The measured data for hybrid excitation with varying prepolarization fields (switched off adiabatically) at $B_0 = 1mT$ are shown in **Figure S5**. Details of the measurements can be found in the methods section (see "vi. Hybrid excitation").

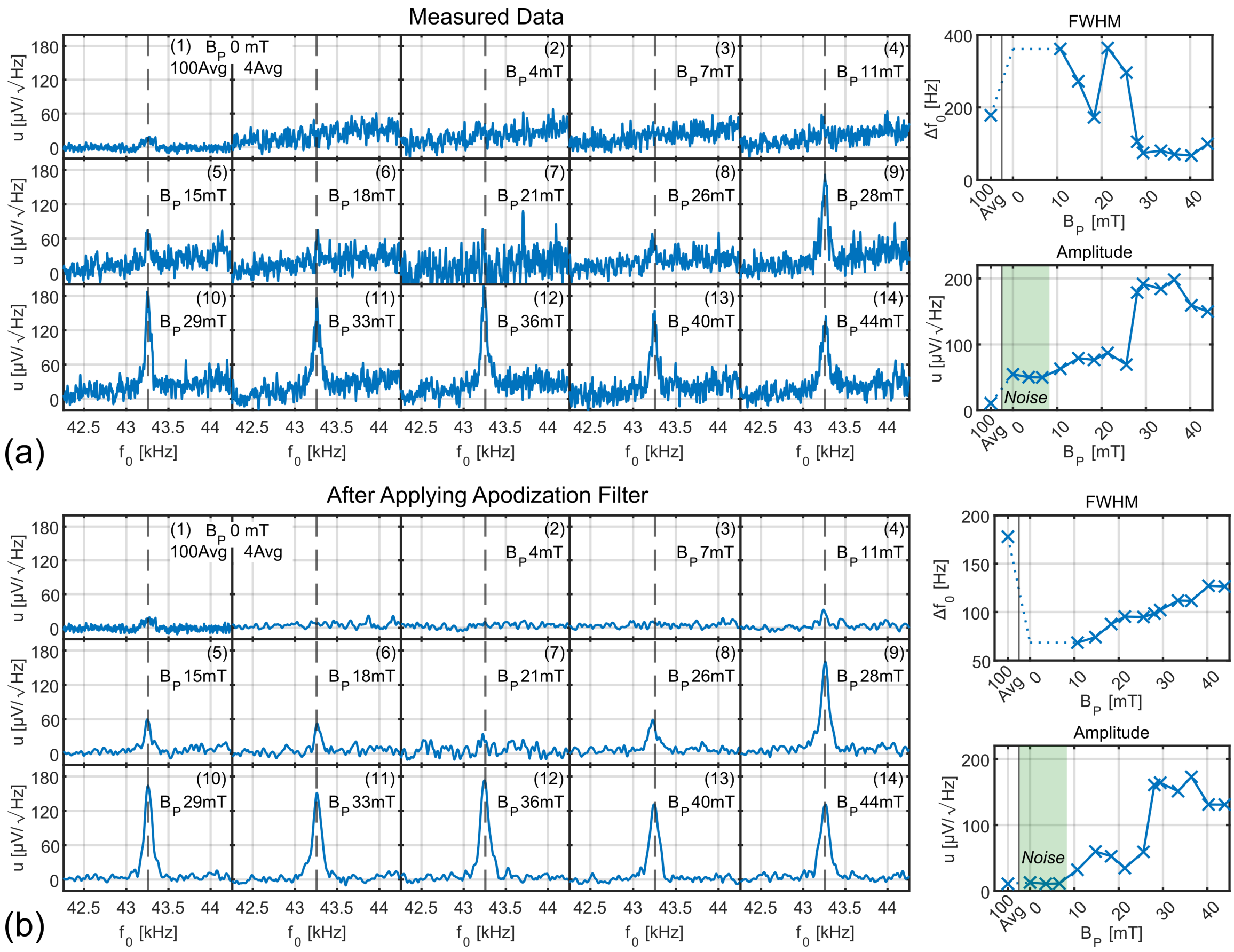

Figure S5: Measured and filtered data. (a) Measured data. (b) measured data but filtered using an apodization filter.

9 Long-term investigation of Aging of the Silicon Carbide Field-Effect Transistors (SiC-nFET)

In the presented operating mode of the SiC-nFETs, rapid switch-off of the polarization coil is enabled via a triggered avalanche of the source-drain barrier of the FET. This involves applying high power to the SiC crystal for short periods of time, which can cause long-term damage to the FET.

The aging of the SiC-nFETs was characterized using a separate setup. The adaption of the polarization circuit involved the integration of a single SiC-nFET, with the configuration set to $R_S = 1k\Omega$ and $R_D = 225\Omega$, thereby ensuring the reliable avalanche-event (modified switchnode capacitances due to lower number of installed SiC-nFETs). The original polarization coil ($L = 80\mu H$) was taken as the inductive load. The SiC-nFET is electrically connected with a screw connection. The SiC-nFET was cooled via an aluminum heat sink with active air flow (cooler temperature in steady state: approx. $T = 35°C$). The coil was passively cooled in a water basin containing approx. $V = 5l$ of water at room temperature. A microcontroller-based sequencer (ATmega 328, Arduino Nano) switches the polarization generator on for $t = 1s$ and then off for $t = 10s$ (avalanche triggering and cooling phase). The current was set to $I = 13.44A$.

Following a predetermined number of breakdowns, the SiC-nFET was removed from the polarization circuit. The on-resistance R_{DSon}, threshold voltage V_{th}, reverse current I_{DSS}, and capacitances C_{iss}, C_{oss} and C_{rss} were then determined using a curve tracer (Keysight B1505A). The steps selected for analysis were 0, 100, 350, 750, 1,500, 2,500, 3,500, and 10,000. The results are shown in Figure S6. With the exception of a minor discrepancy due to measurement tolerance, no signs of aging or damage can be observed. In the standard measurement sequence employed for the field mapping (6s pre-polarization, 1s sampling, and neglecting the cooling of the sample), the result of 10,000 avalanches would be a continuous measurement time of almost 20 hours, or 60 hours when considering the three installed transistors. In principle, after this period, the SiC-nFETs could be replaced as a preventative measure, or an additional measuring circuit could be employed to measure the reverse current and indicate any degradation.

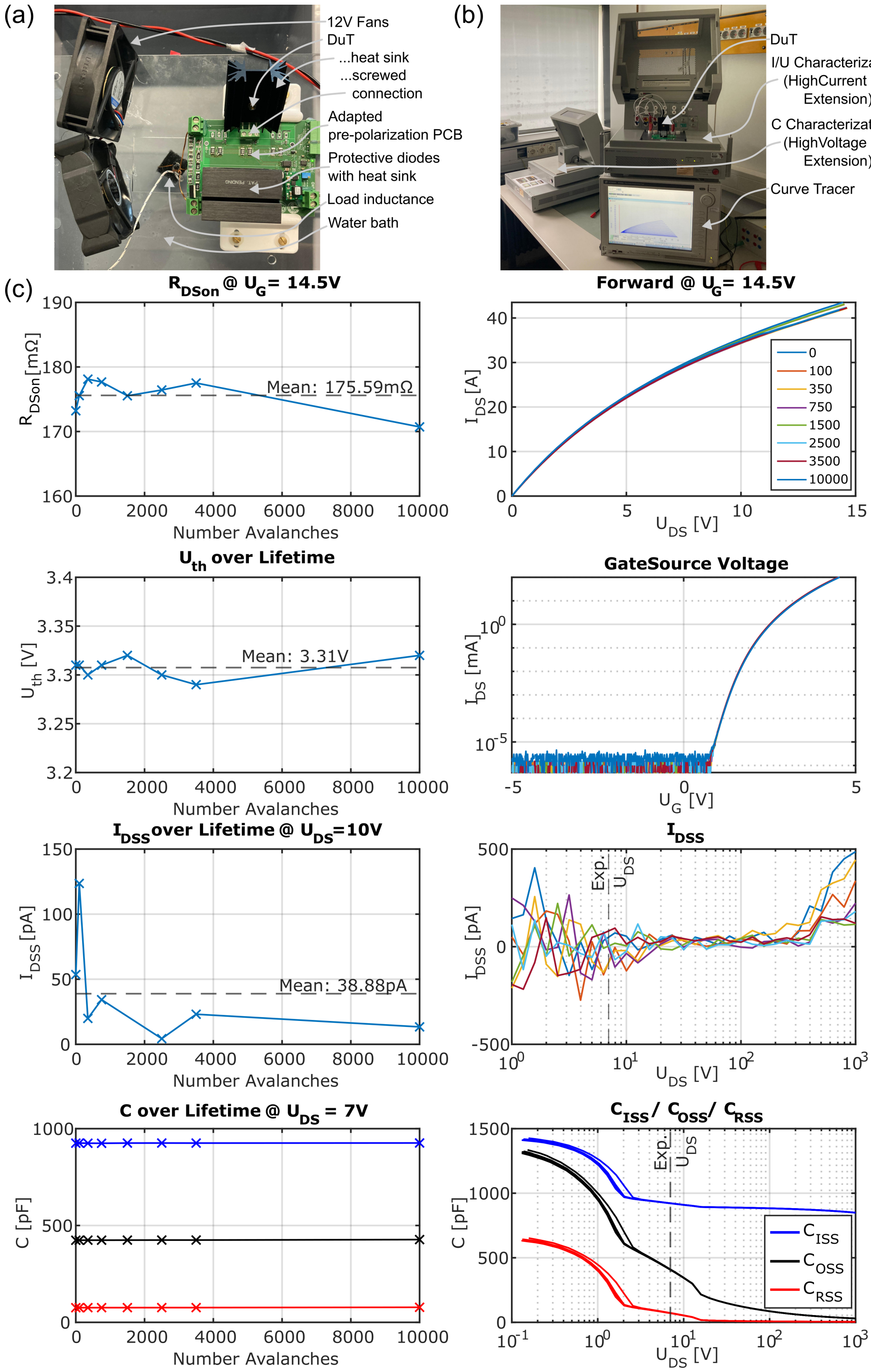

Figure S6: Aging test of the SiC nFET used. **(a)** Test setup during consecutive breakdowns (analogous to the sensor concept). **(b)** Measurement of the dissembled SiC-nFET with a curve tracer. **(c)** Measurement results with the measured traces (right side) and the derived parameters (left side).

10 Analysis of measurement variability at B0 = 45 mT

10.1 Measured NMR spectra

In Figure S7, the measured NMR spectra of measurements inside an unshimmed Halbach magnet at $B_0 \approx 45mT$ are shown. Three measurements were taken for each operating point ranging from $\Delta B_0 = -1228\mu T$ to $\Delta B_0 = 0\mu T$. The expected f_0 is estimated from the known offset coil constant.

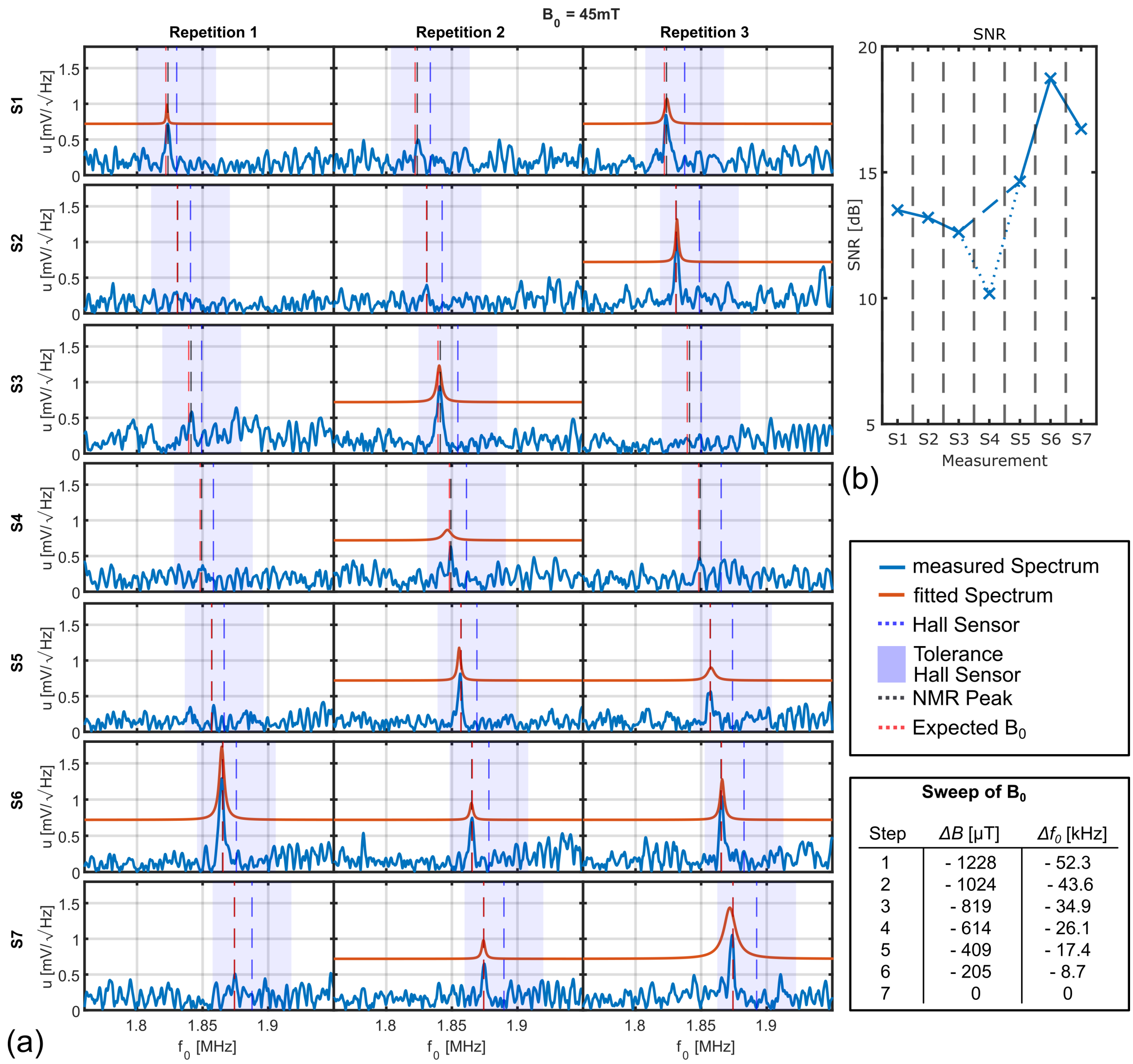

Figure S7: Measured NMR spectra for an unshimmed Halbach magnet at B_0=45mT. The field probe was tuned to f=1.852MHz and pre-polarization set to B_p=70.3mT for t_p=6s. **(a)** Exemplary selection of acquired NMR spectra (absolute value of the complex spectra is shown) in a varying offset field of -1.228mT<ΔB_0<0mT in seven equal distant steps. Absolute value of the complex spectra is shown in blue. The fitted spectra are shown in orange with a slight offset for better clarity (see SI chapter 5 for details). The three horizontal measurements are consecutively measured taken immediately after each other. Data was not averaged. The vertical lines correspond to the measured value from the embedded Hall sensor (with the tolerances of the used sensor added), to the mean Larmor frequency of the three measurements, and to the expected Larmor frequency due to the set of the additional offset coil (see Figure 4e). The measurements were taken at approximately 30-second intervals. **(b)** Frequency drift Δf (relative to the first successful measurement at each setting) derived from (a) at the same operating points (missing points are non-detectable NMR peaks). **(c)** SNR of the most successful measurement (strongest amplitude) derived from (a).

10.2 Detailed simulation analysis of the excitation in the limiting case of adiabatic excitation

As demonstrated in the analysis of the adiabatic condition (SI Section 3), the developed sensor operates in the investigated Halbach magnet predominantly in the adiabatic regime ($B_0 = 45mT, B_P = 70mT, t_{pR} = 1054ns$). Nevertheless, the simulated SNR (Fig. 2d, right panel) indicates a measurable signal. However, the achievable transverse magnetization is highly sensitive to small variations in the excitation waveform at this operating point, making the precise adjustment of the pre-polarization electronics essential.

The rapid discharge of the pre-polarization coil is realized by intentionally operating SiC MOSFETs in avalanche mode. In the present system, three nominally identical SiC MOSFETs are connected in parallel to reduce conduction losses and provide redundancy while avalanche operation is intentionally exploited to rapidly discharge the inductive load.

The avalanche process is inherently stochastic and several physical mechanisms can modify its dynamic behavior[10], [11]. A first contributor to the apparent breakdown-voltage variation on nanosecond timescales is the interaction of fast current transients with parasitic inductances (like the PCB traces), which generate voltage overshoots during high-speed switching. These overshoots are superimposed on the intrinsic avalanche plateau, thereby modifying the effective clamp voltage and shifting the end-of-avalanche timing. Furthermore, repeated avalanche operation can alter semiconductor trap populations and gate-oxide properties[11]. Since SiC MOSFETs exhibit comparatively high interface trap densities, these effects are particularly relevant and may gradually alter which of the installed transistor enters avalanche first and how the avalanche energy is distributed among the paralleled devices after thousands of switching events, even without catastrophic device failure. Additionally repetitive avalanches result in temperature gradients inside the junction and therefore influence the resulting avalanche voltage [10], [12]. Summed up, these effects vary the resulting avalanche voltage and therefore the switch-off dynamics significantly.

Additional operating-point variations arise from the excitation circuit itself. Temperature-dependent changes of the MOSFET on-resistance R_{DSon}, heating of the pre-polarization coil, and small current fluctuations of the deliberately selected elderly current source all contribute to slight variations of the pre-polarization current. The current source was intentionally chosen instead of a modern high-frequency switched-mode supply to avoid interference with the NMR measurement, accepting a small increase in current variability.

The initial simulations assumed an ideal linear current ramp corresponding to a perfectly rectangular avalanche voltage. In contrast, the measured current waveform exhibits a flattened tail and a slight undershoot after avalanche termination. Consequently, several effects influence the excitation profile, resp. the trajectory of the rotating magnetization during the switch off phase:

- variations of the avalanche voltage directly modify the discharge duration and a slightly varying flattened end,
- the stochastic nature of avalanche changes both the avalanche initiation and the time of collapse,
- temperature-dependent variations of the snubber and damping networks slightly alter the transient and damping response,

- together, these effects introduce small variations in both the amplitude and temporal evolution of $B_P(t)$, thereby changing the remaining transverse magnetization after the finished pre-polarization pulse.

These effects were incorporated into an extended Bloch simulation.

The experimentally measured current waveform for ($I_P = 23.9A$) is shown in Figure S8a. Using the known inductance of the pre-polarization coil, the corresponding avalanche voltage was calculated from the time derivative of the measured current and moderate low-pass filtered. To obtain a compact simulation model, the measured current waveform was approximated by a piecewise linear function. Unlike the previous simulations assuming a perfectly linear discharge, the present model additionally accounts for the decay of the avalanche voltage, resulting in a characteristic corner in the current ramp.

Three parameters were identified as having the strongest influence on the resulting transverse magnetization:

- duration of the corner time t_C (Variation of $-400ns \leq \Delta t_C \leq 100ns$),
- zero-crossing time t_{pR} (Variation of $-150ns \leq \Delta t_{pR} \leq 250ns$),
- initial pre-polarization current I_P
  (Variation of $-4mT \leq \Delta B_p \leq 4mT$, resp. $-1.4A \leq \Delta I_P \leq 1.4A$)

A two-dimensional sweep of t_C and t_{pR} is presented in Figure S8b and c, while Figure S8d and e show the corresponding sweep of I_P and t_{pR}. The experimentally determined operating point (denoted as S0) is indicated in all parameter maps and serves as the normalization reference (therefore $\Delta M_T(\text{S0}) = 0\%$).

The simulations reveal an extreme sensitivity to the timing of the corner point t_C. Deviations of only a few nanoseconds result in a total collapse of the available transverse magnetization, preventing the detection of any NMR signal (since the measured signal u is $u \sim M_T$, see SI chapter 2) . This behavior directly results from the highly dynamic magnetization trajectory once the adiabatic condition is nearly fulfilled (see SI chapter 1 and 3).

Variations of the initial current, which also modify the stored magnetic energy and indirectly affect the avalanche duration, mainly shift t_{pR}. Although these variations also explain experimentally observed signal fluctuations, they are generally insufficient to account for a complete disappearance of the NMR signal.

Overall, the simulations demonstrate that the detailed avalanche dynamics (including avalanche termination, temporal evolution of avalanche voltage, the interaction with the snubber and damping networks) strongly influence the magnetization dynamics in this operating regime and therefore the reproducibility of the NMR experiment.

A more comprehensive characterization of the avalanche process, particularly for the parallel operation of the three SiC MOSFETs, would require direct measurement of the avalanche voltage. An initial attempt using a passive high-voltage oscilloscope probe was unsuccessful because galvanic coupling through the oscilloscope introduced large disturbance into the low-voltage gate-drive circuit to noticeably alter the transistor operating point.

An active differential high-voltage probe would be preferable but would require a high technical

demand on specifications, including transient voltage capability well above $U > 2\ kV$, bandwidth exceeding $BW > 500MHz$, and negligible parasitic loading since it would again change the operating point. Such instrumentation was not available for the present study.

A detailed experimental characterization of the avalanche process therefore remains the subject of future work. Beyond optimizing the avalanche discharge itself, future system developments will also aim to operate further outside the adiabatic regime (see Eq. 2.1 in the main manuscript), for example by increasing B_p without increasing the coil inductance, such as through a reduced measurement volume (and therefore smaller coil, resp. smaller L).

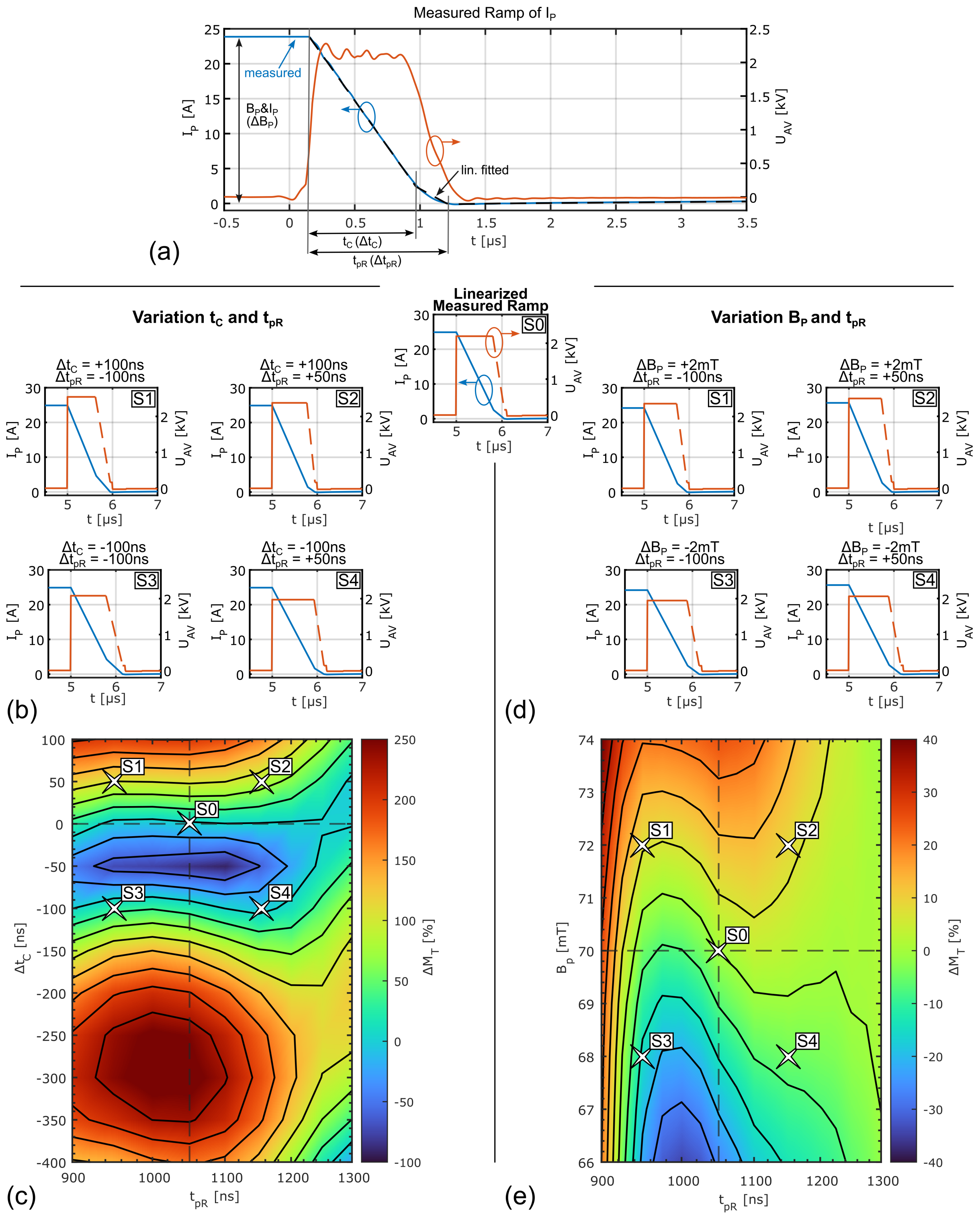

Figure S8: Result of advanced Bloch simulations at B_0=45mT, considering the measured current ramp. (a) Measured current ramp (in blue) for a steady-state current of I_p=23.9A. The current curve was measured using the pickup coil (image in Figure 1f). The measured curve is linearized in individual intervals (in black). The plotted voltage is calculated from the current curve (U=-Ldi/dt) and low-pass filtered (in red). (b) Variation of the corner point position t_C and the length of the turn-off ramp t_{pR}. The curves are set to I_p=25A (used in the NMR experiments in the Halbach magnet). Four representative I/U curves are shown. The operating point S0, which is the linearized characteristic curve from subplot (a), serves as a reference. (c) 2D plot of ΔM_T achieved after the non-adiabatic turn-off process ($M_T(t=t_{pR})$). ΔM_T was referenced to the calculated value at operating point S0 M_T(S0) ($\Delta M_T=M_T/\ M_T$(S0)-1 in percent). (d) Equivalent to (b), but B_p and t_{pR} were varied. (e) 2D plot of M_T achieved after the non-adiabatic shutdown process shown in (d).

10.3 Investigation of electronic induced time delays after completion of pre-polarization

In consideration of the extremely short FID in the Halbach magnet ($T_2^* \ll 1ms$), it is essential for maximum sensitivity that sampling begins immediately after the non-adiabatic switch-off has ended. However, the receive path must be enabled as latest as possible in order to protect it from strong induced transients due to the switch-off. In this setup, it is necessary to consider the reasons for possible delays on the receive side and the pre-polarization side separately in order to minimize them.

i. Receive Electronics

The input of the first LNA is short-circuited via a photocoupler (TLP3431, Toshiba Electronic, Tokyo, Japan) during the pre-polarization phase to protect the receive path. The active HIGH state indicates that the photocoupler is conducting. Therefore, the trigger must fall so that the LNA can receive a signal. The remaining electronics (VGA, filter, ADC) are activated at all times. The photocoupler control line is filtered via an RC low-pass filter and a voltage shifter, positioned directly after the sequencer and directly before the photocoupler. Additionally, the photocoupler has a specified design-related turn-off time.

This total delay was measured experimentally by feeding a sine wave signal ($f = 1.7MHz$) of a frequency generator via a coaxial connection into the frontend input instead of the Rx coil. The RF signal was captured directly after the photocoupler using an installed coaxial connector and an oscilloscope. Furthermore, the trigger was recorded with the oscilloscope connected directly after the sequencer. This results in a total delay of $t_d \approx 1.1ms$ between the falling trigger edge and beginning of the signal measurement (see Figure S9a). This delay is consistent across various experiments and can be accommodated by adjusting the sequence (trigger is switched off slightly earlier than it should).

ii. Pre-Polarization Circuit

The polarization circuit is controlled by a trigger from the sequencer. Active HIGH indicates that the pre-polarization is active (DC current I_p is flowing). When the trigger is deactivated, the non-adiabatic switch-off process is initiated, with a slope according to the installed setup on the PCB. The trigger is filtered using a second-order RC low-pass filter and a voltage shifter. The ACPL-P343 (Broadcom Inc, San José, USA) gate driver ensures galvanic isolation via an integrated optocoupler.

All three SiC nFETs are controlled via a single $R_G = 1\Omega$ each. The gate driver provides a $U_G = +15V$ when it is switched on and a $U_G = 0V$ when it is switched off. Together with the gate capacitance of the SiC-nFETs, this results in a total delay between the falling trigger edge and the transistor actually being switched off. To measure the delay, the signal was captured directly at the gate/source pads of a SiC-nFET using a twisted wire and fed out to a BNC connector (with serial resistors $R = 330k\Omega$ (Source) und $R = 1M\Omega$ (Gate) to minimize capacitive loading). The galvanic isolation process was carried out using a low-budget differential probe (model DP750-100, Micsig, Shenzen, China). The measurement results in a total delay of $t_d \approx 8.8ms$ (see Figure S9b).

iii. Adapted Sequence for Low Field Measurement at B_0 = 45mT

It is evident that these delays pose significant challenges to automated measurement of FIDs, particularly in (inhomogeneous) Halbach magnets. Therefore, the sequence was adjusted, resp. calibrated for the possible delays, so that the triggers are switched off before their actual determined time point to overcome the measured delays. Subsequently, in 20 consecutive experiments, the resulting delay between the switch-off off of the pre-polarization circuit and the switch off of the

photocoupler (LNA starts amplification) was measured (see Figure S9c). Further tolerances were identified, with an overall delay ranging from $2\ \mu s\ < t_d < 26\mu s$ (see Figure S9d).
In order to characterize the sensor in low field with short T_2*, the gate/source voltage was also sampled. The oscilloscope's data acquisition was initiated by the falling edge of the receive trigger. In post-processing, the measured delay of the falling edge of the gate/source voltage was then evaluated. An offset of $\Delta = 1\mu s$ was then added to this time point (where the avalanche-induced collapse of B_p occurs), and data analysis was initiated from subsequent measurement points onwards.

iv. Sequence for Ultra Low Field Measurement at B_0 = 1 to 5 mT

Due to the long FID with $T_2^* > 50ms$, the original sequence was used without further adjustments or consideration of existing delays. During the course of all measurements at all fields, no damage to the LNA was detected.
In post-processing, only data for $t \geq 7.5ms$ resulting from the measured delays ($t_d \approx 8.8ms - 1.1ms$) is evaluated. The additional apodization filter ensures that fluctuations in measurement points at the start of evaluation have minimal impact. In a subsequent development, however, a hardware delay should also be installed for this operating case at ULF.

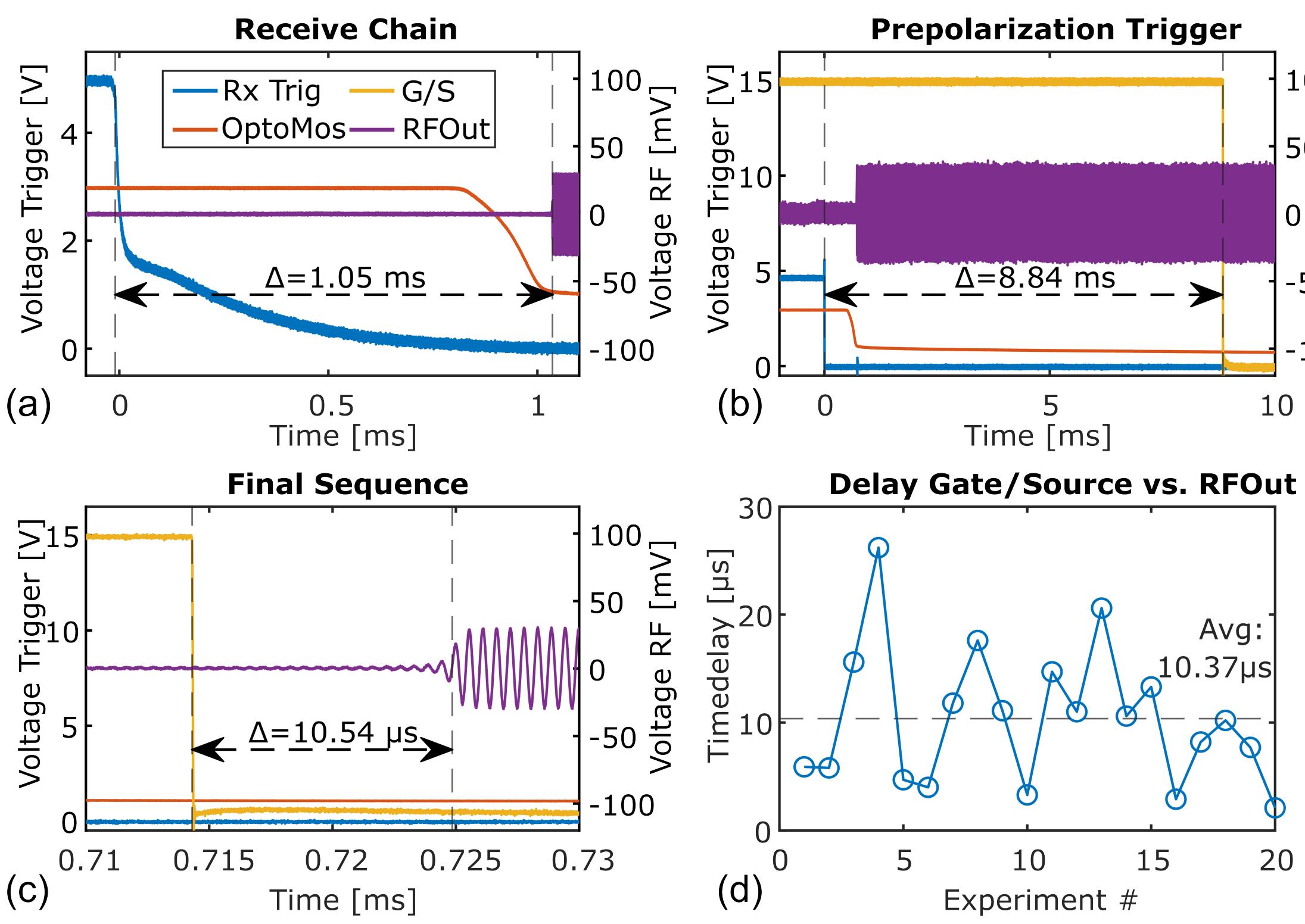

Figure S9: Measured delays in the assembled setup. “Rx Trig” stands for the trigger output of the sequencer that controls the photocoupler (On=Input LNA short-circuited). “OptoMos” is the control input directly at the photocoupler (i.e., the filtered “Rx Trig” signal). “G/S” is the gate/source voltage directly at the SiC-nFET pins. “RF Out” is the RF signal line between the photocoupler and the LNA. If the voltage here is ~0mV, the LNA is effectively protected. **(a)** Investigation of receive electronics. **(b)** Investigation of the pre-polarization circuit. **(c)** Investigation of the final sequence for the LF setup (Halbach measurement). **(d)** Measured variation in delay between different experiments for the LF setup.

11 Investigation of Power Source Instability for the Ultra Low Field magnet

In addition to the inhomogeneity of the magnetic field, the current drift caused by suboptimal control of the current source has a significant impact on the measurement results of the sensor presented. In order to verify the current drift over a longer period of time, the used BCS5/75 current source (75V/5A, HighFinesse GmbH, Tübingen, Germany) was connected to a Helmholtz-like configuration with four coils, and the NMR peak of a water sample was measured with a SQUID sensor [13]. The field was set first to $B_0 = 0.5mT$ and then to $B_0 = 1mT$. The low-pass behaviour of the SQUID electronics and the cooling capacity of the B_0 coil preclude the possibility of higher fields. The SQUID, being operated within a cryostat at a temperature of $4.2K$, is not directly applicable to the build ULF solenoid coil (see SI chapter 10). Consequently, the determined current drift is scaled to the solenoid coil used and should therefore be understood as an indication of the actual current drift (assuming that the different inductance has a negligible effect on the control of the current source). Each measurement point was recorded at a rate of $t_R = 12s$, with a total of 50 sequential measurements being acquired. Two measurement series were recorded at $B_0 = 0.5mT$ $(I = 0.762A)$ and $B_0 = 1mT$ $(I = 1.506A)$. This results in a field drift of $\Delta f = -13.173mHz/A/s$ for the measurement (which means a controller inaccuracy of $\Delta I = -0.47ppm/s$) (see Figure S10). When scaled to the solenoid coil, this results in $\Delta f = -42.775\, mHz/A/s$. For a standard sensor measurement of $t = 10s$ (2s Hall sensor, 6s prepolarization, sampling, and pause), the resultant drift per measurement is $0.2Hz$ (1mT), $0.6Hz$ (3mT) and $1Hz$ (5mT) respectively.

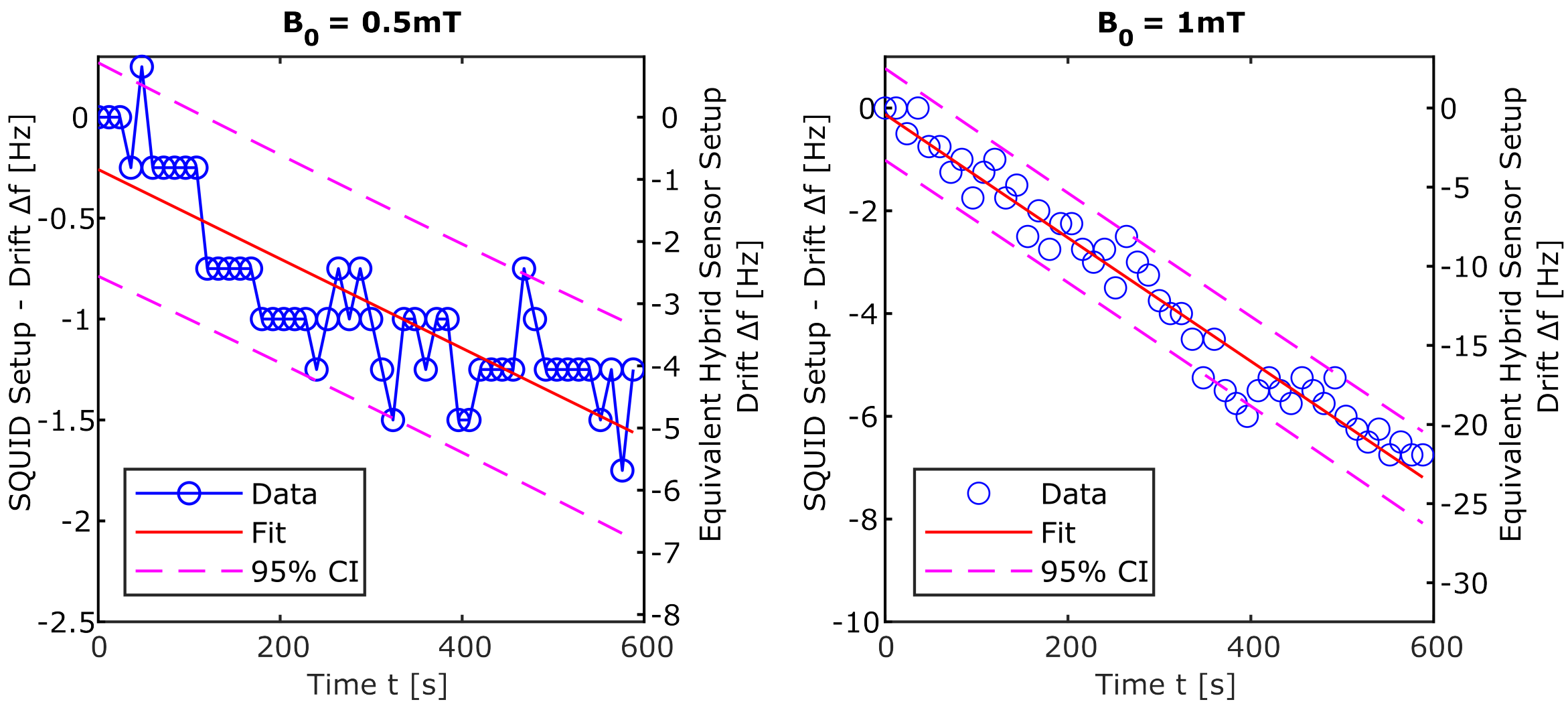

Figure S10: Drift of the current source measured with the SQUID setup for the field $B_0 = 0.5mT$ and $B_0 = 1mT$.

12 Accuracy of frequency measurement with the NMR field probe

To determine the accuracy of the sensor, we use the bin width of the discrete Fast Fourier Transform (FFT). Ideally, the measured NMR signal is an oscillating signal $u(t)$ with Larmor frequency f_0, that is amplitude-modulated by an exponentially decaying function with time constant τ:

$$u(t) = \hat{u} sin(2\pi f_0 t)\exp(-t/\tau) \qquad \text{(S12.1)}$$

To estimate the accuracy of the sensor, we assume that the signal cannot be detected after a time $t_0 = 3\tau$ ($u(t_0)/\hat{u} \approx 5\%$, signals with a $SNR > 27dB$ should be significantly above the noise floor and detectable therefore. The following upper limit is calculated for the bin width of the FFT:

$$Bin = \frac{1}{3\tau} \qquad \text{(S12.2)}$$

and thus, the maximum resolution of the frequency analysis is:

$$\Delta f = \frac{1Bin}{f_0} \qquad \text{(S12.3)}$$

If the SNR is significantly worse (as in the case of the measurement at $B_0 = 45mT$), t_0 should be reduced to $t_0 = 2\tau$ ($SNR > 18dB$).
For the evaluation of the measurements, $\tau = T_2^*$ was set and the measured relaxation times T_2^* were used. This results in an estimated maximum accuracy of Δf:

Table S1: Calculated accuracy of the sensor for the different magnetic fields and the measured relaxation times.

Measurement	B_0	Δf
Figure 5	1 mT	235 ppm
Figure 5	3 mT	26 ppm
Figure 5	5 mT	16 ppm
Figure 8	45 mT	1051 ppm (0.1%)

13 Ultra Low Field Magnet

It was determined that the approach used to develop the Ultra-Low Field MRI (ULF MRI) for this work, facilitates the construction of an easy to replicate magnet and gradient system for ULF MRI, which is both cost-effective and functional (see detailed images in Figure S11). This system has the potential to be particularly advantageous in the context of workshops and for the training of students. It is therefore the intention of this chapter to provide more detailed information on the subject, with reference to the CAD files, which are available in the GitHub repository.

The central B_0 magnet was optimized analytically using Biot-Savart's law (see scripts provided in [14]) and can be calculated on a standard office PC in a few seconds. In the case of the solenoid diameter being substantially larger than the volume to be examined, and the solenoid length being significantly longer than the diameter, a highly homogeneous magnetic field can be assumed (in the present case, $12\ ppm$ for a spherical FOV with a diameter of $20\ mm$). Manufacturing tolerances resulting from the manual winding of the magnet are largely negligible. It is to be expected that minor variations in the number of wound turns will occur due to the tolerance of the copper wire diameter. It is recommended that the optimization of the second layer should be done only subsequent to the completion of the first layer. Additional multi-stage shimming steps, as is the case with Halbach-based systems, are not needed. Any type of cylinder can be utilized as a winding body. In this instance, a Plexiglas cylinder was utilized. However, good results have also been achieved with off-the-shelf drain pipes. Due to electrical power loss, the field is usually limited to $B < 10mT$, although the polarization can be significantly increased by using (not particularly homogeneous) pre-polarization coils (e.g., 70mT is possible in our sensor).

The gradients are straightforward to produce due to their planar structure.

The gradients in the X and Y directions (which exhibit an identical structure, but are rotated by 90° relative to each other) are readily producible. Essentially, these gradients comprise a set of four coils, with a rectangular base area in each spatial direction. The eight corner points of a coil were calculated using a Biot-Savart-based optimization so that the gradient in the FOV (in this case, a spherical FOV with a diameter of $100mm$) is as linear as possible. These eight points were then transferred to a Plexiglas disc ($10mm$ thick). An M10 thread was cut at this point, and a nylon screw was screwed in, leaving approx. $5mm$ of the thread exposed. The enameled copper wire was then simply wound around these corner points. The z-gradient has been designed as a Maxwell coil, which, in principle, geometrically corresponds to a Helmholtz coil with a slightly increased distance between the coils of $d = \sqrt{3}R$ (with the goal of as linear a gradient as possible). In contradistinction to the Helmholtz coil, the two coils are wound in opposite directions with copper wire. In this instance, the winding body comprised Plexiglas rings, with two covers affixed by screws to a central core. The whole gradient system has been adapted from a ready-to-use ULF MRI setup [13]. In order to ensure similar results to the simulation, the individual coils of the gradients must be connected in a way that there is only one connection wire for the power sources. When laying the wires, care must be taken to ensure that two wires with opposite currents (forward and return) are always run in parallel so that the magnetic fields of the current conductors cancel each other out macroscopically.

Established open-source power sources ($I \leq 8A$) can be utilized as power sources for the gradients:
opensourceimaging.org/project/current-driver-for-local-b0-shim-coils/.
The power source for the B_0 coil must have as little drift as possible, whereby the large inductive load contributes to the stability (series resistors may be necessary to stabilize the current regulator of the source). The open-source power source, which has been published for some time now, ($I \leq 5A$):
opensourceimaging.org/project/eez-h24005-bench-power-supply/
is suitable here.
If a pre-polarization coil is installed, it can be operated with any available power supply (no requirements for stability and accuracy).

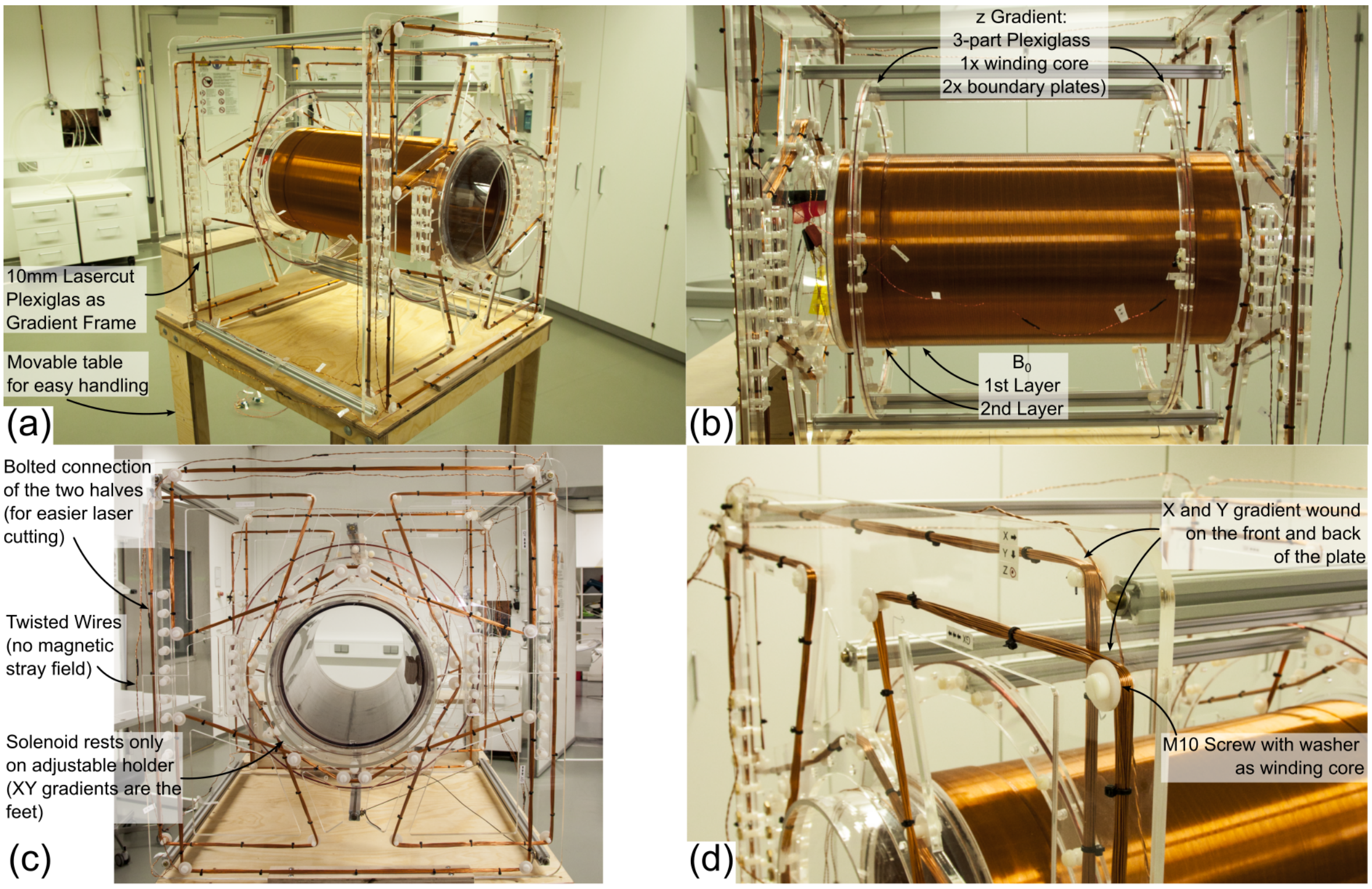

Figure S11: Detailed views of the assembled ULF setup. (a) Perspective overview with easily movable table. (b) Side view of B0 magnet with 2 coil layers. (c) Front view of magnet. (d) Detailed view of XY gradient winding.

14 References

[1] F. Bloch, “Nuclear Induction,” *Phys. Rev.*, vol. 70, no. 7–8, pp. 460–474, Oct. 1946, doi: 10.1103/PhysRev.70.460.

[2] E. M. Haacke, R. W. Brown, M. R. Thompson, and Y.-C. N. Cheng, *Magnetic resonance imaging: physical principles and sequence design*, 1st ed. New York: John Wiley & Sons, Inc, 1999.

[3] D. I. Hoult and R. E. Richards, “The signal-to-noise ratio of the nuclear magnetic resonance experiment,” *J. Magn. Reson.*, vol. 24, no. 1, pp. 71–85, Oct. 1976, doi: 10.1016/0022-2364(76)90233-X.

[4] D. I. Hoult, “The principle of reciprocity in signal strength calculations?A mathematical guide,” *Concepts Magn. Reson.*, vol. 12, no. 4, pp. 173–187, 2000, doi: 10.1002/1099-0534(2000)12:4<173::AID-CMR1>3.0.CO;2-Q.

[5] A. Tannús and M. Garwood, “Adiabatic pulses,” *NMR Biomed.*, vol. 10, no. 8, pp. 423–434, Dec. 1997, doi: 10.1002/(SICI)1099-1492(199712)10:8<423::AID-NBM488>3.0.CO;2-X.

[6] H. A. Wheeler, “Inductance formulas for circular and square coils,” *Proc. IEEE*, vol. 70, no. 12, pp. 1449–1450, 1982, doi: 10.1109/PROC.1982.12504.

[7] P. L. Dowell, “Effects of eddy currents in transformer windings,” *Proceedings of the Institution of Electrical Engineers*, vol. 113, no. 8, pp. 1387–1394, 1966, doi: https://doi.org/10.1049/piee.1966.0236.

[8] C. Rischel, “Fundamentals of Peak Integration,” *Journal of Magnetic Resonance, Series A*, vol. 116, no. 2, pp. 255–258, Oct. 1995, doi: 10.1006/jmra.1995.0016.

[9] M. Veldmann *et al.*, “Improving MR axon radius estimation in human white matter using spiral acquisition and field monitoring,” *Magnetic Resonance in Med*, vol. 92, no. 5, pp. 1898–1912, Nov. 2024, doi: 10.1002/mrm.30180.

[10] F. Ceccarelli, G. Acconcia, A. Gulinatti, M. Ghioni, I. Rech, and R. Osellame, “Recent Advances and Future Perspectives of Single-Photon Avalanche Diodes for Quantum Photonics Applications,” *Adv Quantum Tech*, vol. 4, no. 2, p. 2000102, Feb. 2021, doi: 10.1002/qute.202000102.

[11] J. Wang and X. Jiang, “Review and analysis of SiC MOSFETs’ ruggedness and reliability,” *IET Power Electronics*, vol. 13, no. 3, pp. 445–455, Feb. 2020, doi: 10.1049/iet-pel.2019.0587.

[12] J. O. Gonzalez, R. Wu, S. Jahdi, and O. Alatise, “Performance and Reliability Review of 650 V and 900 V Silicon and SiC Devices: MOSFETs, Cascode JFETs and IGBTs,” *IEEE Trans. Ind. Electron.*, vol. 67, no. 9, pp. 7375–7385, Sep. 2020, doi: 10.1109/TIE.2019.2945299.

[13] K. Buckenmaier *et al.*, “Mutual benefit achieved by combining ultralow-field magnetic resonance and hyperpolarizing techniques,” *Rev. Sci. Instrum.*, vol. 89, no. 12, Dec. 2018, doi: 10.1063/1.5043369.

[14] P. Povolni *et al.*, “Easy scalable, low-cost open-source magnetic field detection system for evaluating low-field MRI magnets using a motion-tracked robot,” *Magnetic Resonance Materials in Physics, Biology and Medicine*, vol. 38, no. 4, pp. 695–714, Aug. 2025, doi: 10.1007/s10334-025-01239-1.
